# Selective solar wax refining with nanoscale zero-valent iron

**DOI:** 10.1038/s41467-026-71010-0

**Published:** 2026-03-30

**Authors:** Yifei Sun, Chengliang Mao, Yunjie Zou, Yuqing Hu, Di Yang, Junzhou Xu, Haopeng Pei, Yanbiao Shi, Zipeng Chen, Wendong Wei, Zhihui Ai, Lizhi Zhang

**Affiliations:** 1 State Key Laboratory of Green Pesticide, Engineering Research Center of Photoenergy Utilization for Pollution Control and Carbon Reduction, Ministry of Education, College of Chemistry, Central China Normal University, Wuhan, PR China; 2 State Key Laboratory of Green Papermaking and Resource Recycling, Shanghai Engineering Research Center of Solid Waste Treatment and Resource Recovery, School of Environmental Science and Engineering, National observation and Research Station of Erhai Lake Ecosystem in Yunnan, Yunnan Dali Research Institute, Shanghai Jiao Tong University, Shanghai, PR China; 3 State Key Laboratory for Pollution Control and Resource Reuse, College of Environmental Science and Engineering, Tongji University, Shanghai, PR China; 4 School of Energy and Materials, Shanghai Polytechnic University, Shanghai, PR China; 5 State Key Laboratory of Hydraulics and Mountain River Engineering, College of Architecture and Environment, Sichuan University, Chengdu, PR China

**Keywords:** Photocatalysis, Photocatalysis, Catalytic mechanisms

## Abstract

Wax refining transforms raw wax into high-grade product by increasing the uniformity of molecular carbon-chain length and removing impurities. Conventional thermochemical approaches face inherent limitations in effectively reducing carbon-chain dispersity (*Ð*) due to their non-selective C-C scission in feedstock waxes. This mechanistic constraint consequently necessitates energy-intensive downstream processing involving fractional distillation and purification. Here, we demonstrate a selective solar wax refining method that upgrades the raw polyethylene wax by significantly reducing its *Ð* from 2.5 to 2.0 in one step with a reaction selectivity beyond 80%, enabled by nanoscale zero-valent iron (nZVI) catalyst and sunlight. At the nZVI-wax interface, photons activate C-H bonds to provide hydrogen atoms, while localized hot spots mediate C-C bonds cleavage via hydrogen atom transfer initiated hydrocracking and concurrent evaporative desorption of fragmented wax product, thereby achieving precise control over carbon-chain dispersity. This work exemplifies the potential in precise and efficient solar refinery.

## Abstract (web summary)

Current thermal wax refining methods are energy intensive. Here, the authors report a photothermal process using a nanoscale zero-valent iron catalyst and sunlight to refine low-grade waxes into high-grade waxes with narrow distributions.

## Introduction

Waxes (C_n_H_m_, *n *≥ 18), majorly comprising alkyl C-C and C-H bonds, serve as industrial feedstocks for lubricants, candles, cosmetics, and pharmaceuticals. Wax can be derived from polyolefin cracking^1–4^, controlled olefin polymerization^5^, or petroleum extraction^6^, with a global production of 4.5 Mt annually. Furthermore, wax is envisioned to be produced in large quantity through waste plastics in future. Recent advances suggest that dominant chemical strategies such as pyrolysis and metathesis can produce wax via plastic recycling^2–4^. However, regardless of the origin, most raw waxes are of low value (<$600/ton) due to their broad molecular weight distributions (dispersity *Ð* = *M*_*w*_*/M*_*n*_ > *2.1*, where *M*_*w*_ and *M*_*n*_ represent weight-average and number-average molecular weights, respectively) and abundant impurities, which cannot meet stringent requirements for high-end (as high as $6300/ton) applications^7–9^. To improve their utility and value, raw waxes are refined physically or chemically, such as solvent extraction, thermochemical cracking and distillation under high temperature^1^. These strategies remove impurities through different solubility or boiling points, simultaneously breaking the long-chain wax molecules to obtain lower dispersity *Ð*. Unfortunately, physical methods suffer from low selectivity, requiring multiple repetition cycles and ultra-precise yet challenging temperature control, such as the cutting-edge large-scale distillation tower of 70 m in height. Thermochemical cracking, on the other hand, suffers from non-selective bond cleavage because the wax’s molecular backbone contains similar C-C/H bonds, generating low-value byproducts such as gaseous C_1_-C_4_ hydrocarbons (<$1,000/ton) and mixed-phase residues (<$600/ton) that requiring post physical separation.^10–13^ These facts underscore inherent limitations in selectivity, efficiency, and fossil-energy density of wax thermal processing.

Solar refinery has emerged as a sustainable alternative to conventional thermal refining process, enabling precise chemical processing under solar irradiation^14–21^. Unlike phonon-mediated thermal processes, solar refinery leverages photon-initiated highly mobile charge carriers over timescales ranging from femtoseconds to nanoseconds and subsequently generated phonons via non-radiative relaxation of charge carriers in the timescale of picoseconds and beyond^22^. Such photon–phonon synergy induces transient non-equilibrium molecular rotational–vibrational–electronic activations^23–25^. Since part of these transients occur much faster than nuclei can respond as stated in the Franck-Condon principle, solar refinery can enable highly localized physical or chemical processes that are not observed in conventional thermal refinery processes. Specifically, charging one of two identical chemical bonds/atoms (O=C=O, H-O-H, or H-H) via photon-induced charge carriers can result in their heterolysis assisted by highly localized photothermal heating^26–38^. Therefore, using the photo-associated mechanism is likely to generate an effective route to activate C-C bonds for selective wax conversion, but it remains unexplored. Furthermore, the implementation of the idea remains hindered by wax’s weak sunlight absorption and the high energy barrier for C-C bond scission (348 kJ/mol). Overcoming these limitations requires materials capable of efficient solar photon harvesting and photothermal conversion.

To this regard, nanoscale zero-valent iron (nZVI) presents unique advantages for solar-driven wax refining. As an earth-abundant ferromagnetic material containing Fe@Fe_2_O_3-*x*_ core-shell structures, nZVI exhibits broadband light absorption and plasmon-enhanced hot electron generation in its metallic core^39–41^. These electrons transfer to the iron oxide shell, where high electronic resistivity and short electron mean free paths promote rapid heat generation^42,43^. This photo(thermal) chemistry positions nZVI as an untapped yet promising catalyst for solar-driven wax refining. Herein, we demonstrate single-step solar refining of raw wax (*M*_*w*_ = 988 g/mol, *Ð* = 2.5) into high-grade product (*M*_*w*_ = 564 g/mol, *Ð* = 2.0) using nZVI. Through wavelength- and intensity-dependent activity tests, in-situ diffuse reflectance infrared Fourier transform spectroscopy (DRIFTS), electron paramagnetic resonance (EPR) spectroscopy, and finite-difference time-domain method (FDTD) simulations, we elucidate the critical role of photon and localized heating in enabling selective C-H/C-C bond cleavage. This work establishes a sunlight-driven platform for sustainable wax refining, circumventing the limitations of conventional thermochemistry.

## Results

**Fig. 1:**
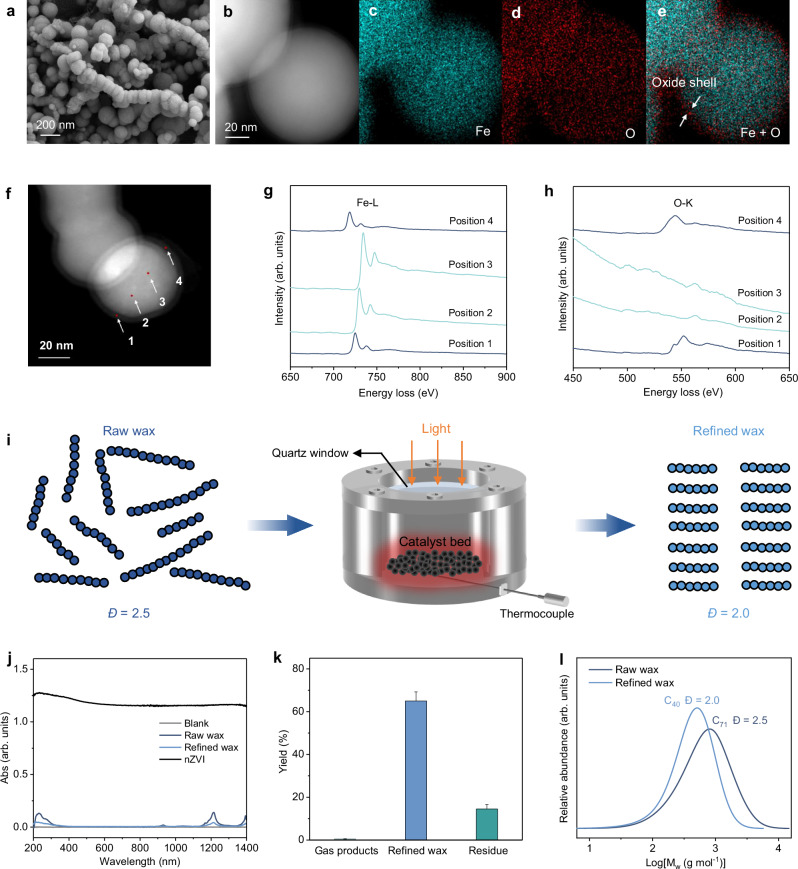
nZVI-enabled solar wax refining. **a** SEM image of nZVI. **b** HAADF-STEM images of nZVI and corresponding XEDS elemental mapping of **c** Fe (blue), **d** O (red) and **e** overlap. **f** HAADF-STEM micrograph of nZVI, and corresponding **g** O-K edge and **h** Fe-L edge EELS spectra at different positions. **i** Schematic illustration of the solar wax refining system. **j** DRS spectra of raw wax, refined wax, and nZVI. **k** Mass yield of the gas, refined wax and solid residue products. Error bars represent standard deviations of three independent measurements. **l** Molecular weight distribution of raw and refined wax according to HT-GPC measurements dissolved in 1,2-dichlorobenzene by heating at 145 °C.

The nZVI catalyst was synthesized through a wet chemical reduction, exhibiting a unique nano-necklace morphology composed of core-shell structured Fe@Fe_2_O_3-*x*_ nanospheres, as verified by scanning electron microscopy (SEM) and high-angle annular dark-field scanning transmission electron microscopy (HAADF-STEM) with elemental mapping (Fe, O) and electron energy-loss spectroscopy (EELS) analysis (Fig. 1a–h, Supplementary Fig. 1). For wax refining, 100 mg nZVI was homogenously blended with 100 mg raw PE-wax in a 50 mL Ar-purged batch reactor (Fig. 1i, Supplementary Fig. 2). Under simulated sunlight, the nZVI triggered photothermal conversion, generating localized thermal energy that selectively cleaved C-C/C-H bonds in the wax matrix (Fig. 1j). The resulting short-chain hydrocarbons subsequently vaporized from the heated nZVI surface and condensed on the cool interior walls of the reactor, achieving spatial separation from gaseous products and catalyst-bed residues (Supplementary Fig. 3).

Initial refinement trials (15 h irradiation) yielded 70 mass% premium wax product, 14% solid residues, and 0.5% gaseous products at the optimal reactant-to-catalyst mass ratio of 1:1 (Fig. 1k and Supplementary Fig. 4). High-temperature gel permeation chromatography (HT-GPC) analysis revealed significant molecular restructuring: the refined wax showed narrowed chain-length distribution (*Ð* = 2.0, *M*_*w*_ = 564 g/mol, C_40_ equivalent) compared to raw material (*Ð* = 2.5, *M*_*w*_ = 988 g/mol, C_71_ equivalent), indicating that the refining process selectively cleaved the long-chain (C_151_-C_315_) C-C bonds in the raw wax by 2 to 8 times, along with a few residual H and C atoms forming by-products (Fig. 1l). Catalyst-bed residues primarily comprised surface-adsorbed carbon species, while gas chromatography (GC) with flame ionization detector (FID) and thermal conductivity detector (TCD) identified gaseous products as C_1_–C_4_ hydrocarbons and H_2_ (Supplementary Fig. 5).

To evaluate the quality of the refined wax, we conducted rigorous assessment of its chemical purity, with particular attention on the content of aromatic impurities and <C_18_ hydrocarbons. The aromatic content below 3% serves as a mandatory metric for commercially acceptable high-grade wax due to safety considerations, while <C_18_ hydrocarbon mixtures are usually classified as non-wax fuel components. Fourier-transform infrared spectrum (FT-IR) of refined wax revealed characteristic bands exclusively corresponding to linear hydrocarbons: C-H stretching (2800–3000 cm^−1^), bending (1400–1500 cm^−1^), and CH_2_ rocking vibrations (700–750 cm^−1^), and no aromatic signatures, typically above 3000 cm^−1^ and within 1650–1450 cm^−1^, could be detected (Supplementary Fig. 6)^44^. The proton nuclear magnetic resonance (^1^H NMR) spectrum further provided detailed structural insights into the refined wax (Supplementary Fig. 7). A sharp singlet at δ 1.1–1.4 ppm, corresponding to -CH_2_- groups in linear chains, and a triplet at *δ* 0.8–1.0 ppm, assigned to terminal -CH_3_ groups, confirmed the dominance of unbranched alkanes. Minor signals at δ 2.4–2.7 ppm (allylic protons, C=C-CH_2_-) and δ 4.9–5.9 ppm (vinylic protons, -CH=CH-) revealed trace unsaturated bonds^16,45^. Quantitative analysis via peak area integration indicated that the refined wax contained above 99% alkyl and 0.72% olefinic linear hydrocarbons. The absence of peaks between δ 7.0 and 7.7 ppm verified that isomerized, cyclic, or aromatic components were not formed during the solar-driven C-C cleavage. Consistently, the π–π* transition absorption peak of aromatic rings, which typically located at 254 nm, was not detected via UV-Vis spectroscopy (Supplementary Fig. 8). It is known that HT-GPC, FT-IR, ^1^H-NMR and UV–Vis characterize hydrocarbons of different carbon chain lengths in wax as a whole, thus potentially neglecting the details in shorter chain hydrocarbons. To this regard, the shorter-chain hydrocarbons (<C_30_) were then analyzed by gas chromatography with mass detector (GC-MS) and GC-FID. The GC-MS spectrum of the refined wax displayed strong primary peaks of linear hydrocarbons ranging from C_12_ to C_28_. When the spectrum was zoomed in, each primary C_*n*_ peak could be resolved into a doublet, indicative of a linear olefin and a linear alkane of the same carbon number according to the mass pattern analysis, without detectable aromatics (Supplementary Fig. 9). Quantitative analysis by GC-FID suggested that the refined wax contained more than 99 wt% C_18_-C_30_ linear-chain waxy alkanes and olefins and less than 1% C_12_-C_17_ hydrocarbons (Supplementary Fig. 10). These results collectively demonstrated that the product was high-purity (>99 wt% linear C_18+_ alkanes), aromatics-free high-grade wax, suggesting that the solar wax refining process proceeded through selective C-C/C-H bond cleavages without isomerization or cyclization competitive reactions.

The refined product met premium wax specifications with *Ð* = 2.0 and >99% purity, commanding market values exceeding $1300/ton (Supplementary Table 1), outperforming conventional thermal methods by eliminating energy-intensive reactors, fossil-derived heat, and solvent inputs. In comparison, the wax refining capacity of control samples including commercial Fe powder, Fe_2_O_3_, and Fe_3_O_4_, or conventional thermochemical wax refining were significantly lower (<60% wax yield; Supplementary Fig. 11). This result highlights not only our highly efficient nZVI material but also the nZVI-enabled solar-driven strategy, which bodes well for a sustainable, high-efficiency wax upgrading (Supplementary Table 2).

As far as we are concerned, such a solar-driven strategy for wax refining was not reported previously. To understand its reaction fundamentals, the first task is to identify the respective contribution of light and heat to the yield of refined wax product. The challenge lies in the regulation of photon and heat in a highly controlled and independent manner during activity test. To amplify, it is ideal to retain reaction temperature while gradually tune photons to see the change in yields of waxes or vice visa, but heat is initiated by photon and thus temperature and photons typically change concurrently. Fortunately, photons can be tuned by either flux (also known as light intensity) or energy (light wavelength) due to its wave-particle dualism, and temperature can be controlled by the external electric heating power. Therefore, the combination of light-intensity and wavelength-dependent activity tests with control thermal test can help to resolve the dilemma.

**Fig. 2:**
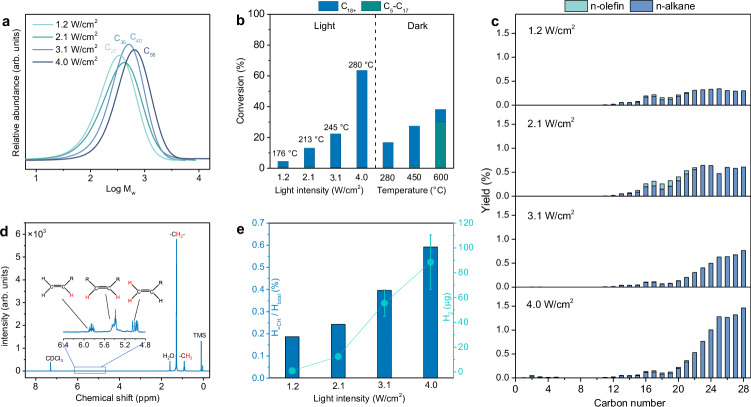
Solar wax refining under varied light intensities. **a** HT-GPC analyses of wax products obtained under different light intensities. **b** The C_5_-C_17_ and C_18+_ content of wax products obtained under different light intensities and dark heating. **c** The detailed carbon number distributions as quantified by GC-FID. **d** ^1^H NMR spectra of wax product obtained under 3.1 W/cm^2^. Solvent: CDCl_3_. The inset illustration is a partial enlarged view as indicated by the dotted box. **e** Proportion of olefinic protons in wax products obtained under different light intensities quantified by ^1^H NMR and corresponding H_2_ production during the refining process. Error bars represent standard deviations of three independent measurements.

The light intensity-dependent wax refining was tested under full-spectrum sunlight of 1.2, 2.1, 3.1, and 4.0 W/cm^2^. The HT-GPC results confirmed that nZVI selectively converted raw PE-wax (*Ð* = 2.5) with a long carbon chain length of C_71_ (*M*_*w*_ = 988 g/mol) into high-grade waxes with shorter carbon chain lengths of C_27_ (*Ð* = 2.6), C_36_ (*Ð* = 3.2), C_40_ (*Ð* = 2.0), and C_55_ (*Ð* = 1.9) under light intensities of 1.2, 2.1, 3.1, and 4.0 W/cm^2^, respectively (Fig. 2a). GC-FID analysis threw further insight into the selective formation of <C_30_ refined waxes. It is found that increasing light intensities can suppress the formation of unfavored <C_18_ non-wax hydrocarbons, with their relative content decreasing from 17%, 9%, 2%, to 1% under 1.2, 2.1, 3.1, and 4.0 W/cm^2^, respectively (Fig. 2b, c, and Supplementary Fig. 12). In contrast, thermal heating produced less refined wax (<30%) from nZVI than that under light, despite the use of higher catalyst bed temperatures (280–600 °C; Notably, industrial thermochemical wax production typically operates between 400–500 °C). Notably, quantitative ^1^H-NMR and GC-FID/TCD analyses also evidenced that increasing light intensity elevated the ratio of olefinic proton (H_C=C_/H_total_) from 0.19% to 0.59% in wax, paralleled by the increase of H_2_ yield from 1 to 88.5 μg during the wax refining process (Fig. 2d, e, Supplementary Fig. 13, Supplementary Table 3). This correlation suggests the higher the photon flux, the more C-H but less C-C bond cleavages in the raw wax.

**Fig. 3:**
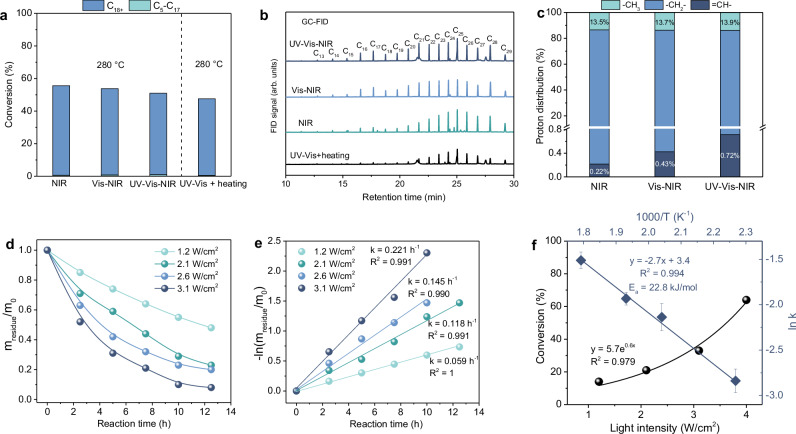
Wavelength-dependent solar wax refining and kinetic study. **a** Content of C_5_-C_17_, C_18+_ and **b** corresponding GC-FID spectra for wax product under UV–Vis–NIR, Vis–NIR, NIR irradiation at 280 °C, and UV–Vis irradiation with thermal heating at 280 °C. **c** Proton distributions of wax product under UV–Vis–NIR, Vis–NIR, NIR irradiation at 280 °C. **d** Conversion curves at different light intensities (1.2, 2.1, 2.6, and 3.1 W/cm^2^), and **e** corresponding quasi-first-order kinetic model fitting. **f** The Arrhenius plot and the exponential correlation between the wax conversion and the light intensity for solar-driven wax refining. Error bars represent standard deviations of three independent measurements.

Subsequently, light wavelength-dependent activity was tested under UV (300–420 nm), UV–Vis (300–800 nm), and UV-Vis–NIR (300–2500 nm) irradiation at a fixed temperature (280 °C), with thermal heating supplemented if needed. This experimental setting retains the reaction temperature while tuning the photon energy independently. The results evidenced that the relative content of short-chain C_5_-C_17_ non-wax hydrocarbons increased from 1.1% of NIR-driven, 1.5% Vis-NIR-driven, to 2.0% of UV–Vis–NIR-driven wax refining, respectively, as quantified by GC-FID (Fig. 3a, b). Under UV–Vis and external thermal heating condition, the wax refining process obtained less non-wax hydrocarbons (1.1%) compared to that (2.0%) under UV–Vis–NIR, but the yield of refined waxes was lower (47% vs 55%). Under sole light irradiation, the dispersity of obtained wax *Ð* decreased from 2.0 to 1.9 and 1.8 under UV-Vis-NIR-, Vis-NIR-, and NIR-driven wax refining, respectively (Supplementary Fig. 14). Furthermore, ^1^H NMR analysis demonstrated that the relative intensity of olefinic protons (H_=CH_/H_total_) increased from 0.22%, 0.43% to 0.72% in the refined wax, along with the increased H_2_ by-product from 142 to 206 μg during the activity test processes (Fig. 3c and Supplementary Fig. 15, 16, Supplementary Table 4).

Combining light intensity- and wavelength-dependent results, at a constant reaction temperature, the wax refining reaction saw an enhanced C-C/H bond cleavage through increasing the energy of photons, while simultaneously increasing photon energy and reaction temperature resulted in increased C-H but decreased C-C cleavage. The latter, which increased C-H but decreased C-C cleavages, can realize the selective formation of high-quality wax. This tunable selectivity enables targeted production of premium waxes with chain lengths optimized for specific applications: from high-melting lubricants to low-viscosity surfactants through simple adjustment of illumination parameters.

We subsequently conducted the kinetic study to understand the selective solar wax formation. First, the time course of the wax refining was recorded under 1.2–3.1 W/cm^2^ full-spectrum sunlight (Fig. 3d). As the reaction proceeded, the ratio of residual mass decreased exponentially in 10 h, which can be fitted to the pseudo-first-order kinetics with a derived reaction constant *k* of 0.221 h^−1^ under 3.1 W/cm^2^. Decreasing the light intensity from 3.1 to 1.2 W/cm^2^ did not change the reaction kinetic functional, but decreased the reaction constant *k* from 0.221 to 0.145, 0.118, and 0.059 h^−1^ under 3.1, 2.6, 2.1, and 1.2 W/cm^2^, respectively (Fig. 3e). Furthermore, the wax conversion increased exponentially with the light intensity under 1.2–3.1 W/cm^2^ (Fig. 3f), which contrasts with the linear relationship between activity and light intensity typically observed for pure photochemical process^46^. When logarithms of the reaction constant *k* under 1.2–3.1 W/cm^2^ were plotted with the reciprocal of temperature (photothermal heating-induced bulk temperature of the catalyst bed), a good linear correlation was identified, a trend analogous to the temperature-dependent behavior commonly observed in conventional thermochemical reactions. The apparent activation energy *E*_*a*_ derived was 22.8 kJ/mol using the Arrhenius equation. These results conspire a unique light-assisted thermochemistry of which the reaction rate is dominated by thermochemistry, while the reaction selectivity is significantly higher than that of pure thermochemistry.

**Fig. 4:**
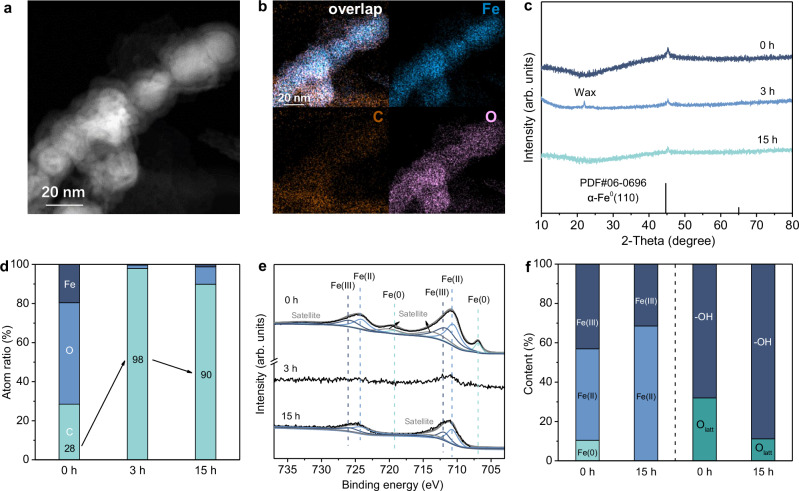
Changes of nZVI during solar wax refining. **a** HAADF-STEM image and corresponding elemental mapping (**b**) of the catalyst after 15 h reaction (Fe-blue, O-pink, C-orange and their overlapping images). **c** XRD patterns, **d** Fe, O, and C atomic ratio, and **e** Fe *2p* XPS spectra of nZVI after 0, 3 and 15 h reaction. **f** The content of Fe and O species quantified by Fe *2p* and O *1s* XPS spectra. Due to the buildup of unreacted wax residues, the Fe *2p* spectral signal intensity of nZVI diminished significantly after 3 h of reaction, preventing quantitative Fe(II)/Fe(III) analysis.

To further elucidate the mechanism for efficient and selective wax formation via light-assisted thermochemistry, the surface chemistry is studied in detail. HAADF-STEM imaging and XEDS mapping were conducted for nZVI before and after wax refining reaction, revealing that the core-shell structure of nZVI remained unchanged, with significant enhanced carbon signals on the surface of reacted nZVI (Fig. 4a, b). Notably, such carbon signals were induced by the accumulation of melt wax and corresponding fragments, instead of the carbon contaminant from surroundings, as pristine nZVI exhibited negligible carbon signals (Supplementary Fig. 17). This point was further supported by X-ray diffraction (XRD) measurements, where the α-Fe (110) peak (2*θ* = 45°) of nZVI remained unaltered throughout the reaction, and the wax peak (2*θ* = 22°) emerged after 3 h of reaction while disappeared after 15 h of reaction (Fig. 4c). Furthermore, X-ray absorption near edge structure and fine structure (XAFS) spectra also evidenced the reduction of nZVI during the reaction, with the bulk material maintained near Fe(0) (Supplementary Fig. 18). The STEM, XRD and XAS results not only confirmed the structural integrity of nZVI during the wax refining reaction, but also indicated the melted wax on the nZVI surface in the first 3 h, followed by its reduction from 3 h to 15 h, as indicated in our time-dependent activity test. X-ray photoelectron spectroscopy (XPS) was then applied to probe the surface chemical environment of nZVI catalyst before and after the wax-refining reaction. Aligning with HAADF-STEM and XRD results, C *1s* XPS spectra confirmed the accumulation of carbon species on the reacted nZVI, with the surface carbon content increased from 28% to 98% from 0 to 3 h, followed by a gradual decline to 90% after 15 h (Fig. 4d and Supplementary Table 5). Furthermore, the high-resolution Fe *2p* XPS spectrum of nZVI contained peaks at 706.9, 710.6, and 711.9 eV, corresponding to Fe(0), Fe(II) and Fe(III), respectively, with the peak area ratio of 10.6%: 46.5%: 43.1% (Fig. 4e and Supplementary Table 6). After 15 h reaction, the peak assigned to metallic Fe(0) vanished completely, the portion of Fe(III) peak area decreased from 43.1% to 31.5%, and that of Fe(II) increased from 46.5% to 68.5%. These changes indicated a disproportionation reaction, likely via Eq. 1, and a partial reduction on the nZVI surface (Eq. 2). This interpretation was further solidified by a concurrent decrease of surface lattice oxygen (O_latt_) content in nZVI as revealed by O *1s* XPS spectra, which dropped from 32% before reaction to 11% after reaction (Fig. 4f, Supplementary Fig. 19). Given the core Fe(0) is encapsulated by iron oxide shell within nZVI, its oxidation involves a solid-solid interfacial reaction with Fe(III) in the oxide shell, which is likely facilitated by atomic iron/oxygen migration and electron transfer under high temperature. The reduction of surface iron oxide is very easy in reductive atmosphere, as the case in the refining process involving hydrocarbon wax and its fragments such as molecular and atomic hydrogen. Thus, under the reaction condition, the active surface of the catalyst is a mixed-valence Fe(II)/Fe(III) iron oxide.

1 $$2{{\rm{Fe}}}({{\rm{III}}})+{{\rm{Fe}}}(0)=3{{\rm{Fe}}}({{\rm{II}}})$$

2 $${{{\rm{Fe}}}({{\rm{III}}})}_{2}{{{\rm{O}}}}_{3}+2{{{\rm{e}}}}^{-}=2{{\rm{Fe}}}({{\rm{II}}}){{\rm{O}}}+{{{\rm{O}}}}^{2-}$$

**Fig. 5:**
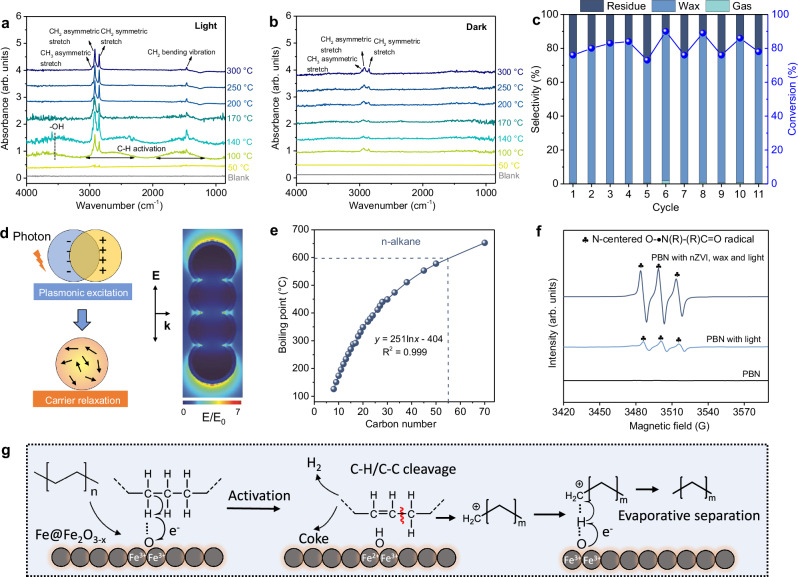
Proposed surface reaction mechanism. In-situ DRIFTS spectra of wax refining driven under light (**a**) and dark (**b**). **c** Reusability of the nZVI catalyst for wax refining under light. **d** Plasmonic excitation and subsequent relaxation processes of nZVI with FDTD simulation, *λ* = 1400 nm. **e** Boiling point vs. the carbon number of n-alkane, and deduced nanometric temperature under irradiation according to the carbon number of the evaporatively separated wax product. **f** EPR spectra of PBN, PBN with light and PBN with wax refining reaction. **g** Proposed mechanism of solar wax refining.

To understand the wax refining on the mixed-valence Fe(II)/Fe(III) iron oxide surface at molecular level, we conducted in-situ DRIFTS measurements under dark and light conditions (Fig. 5a, b). Characteristic wax vibrations emerged as the reaction temperature was above 50 °C, with intensities of these peaks increased from room temperature to 170 °C and then stabilized throughout 170–300 °C, including CH_3_ asymmetric stretching at 2960 cm^−1^, CH_2_ asymmetric stretching at 2920 cm^−1^, symmetric CH_2_ stretching at 2850 cm^−1^ and C-H bending at 1460 cm^−1^ ^44,47^. The onset temperature for wax C-H dissociation was deduced as 100 °C, where a transient hydroxyl stretching band (>3200 cm^−1^) emerged, suggesting hydrogen atom transfer from wax to the lattice oxygen of the iron oxide shell after cleavages of C-H bonds. Interestingly, the hydroxyl stretching band emerged along with the broadening of C-H bonds around 2300–3200 and 1200–2000 cm^−1^ at 100–140 °C. Given the position of a DRIFTS peak is associated with the length of certain chemical bond, such broadening suggested the simultaneous elongation and shortening of C-H bonds in the wax, i.e., C-H bond activation. Under higher temperature that no hydroxyl stretches could be observed, such broadening disappeared as well. Such a C-H bands broadening could not be observed in the absence of light under same temperature. These results suggested that the photon was crucial for C-H activation, and C-H dissociation was followed by C-H activation under relatively low temperature (100–140 °C). C-C cleavage in raw wax can be evidenced by the reduction of wax’s shoulder -CH_3_ stretches at 2960 cm^−1^. Compared to the relatively mild-condition C-H cleavage, the C-C cleavage occurred at temperature higher than 200 °C under light irradiation, and smaller product/intermediate peak intensities could be observed under dark condition within 300 °C. Notably, the -CH_3_/-CH_2_ intensity ratios under light and dark conditions were 0.16 and 0.64, respectively. Given the -CH_3_ is the terminal moiety of an alkyl fragment, this result indicated that thermal heating drives more C-C bonds scission in wax and photothermal drives the controllable scission C-C bonds (Supplementary Fig. 20). All these results correlated wax refining performance with preferential C-H activation prior to C-C cleavage, highlighting the importance of light-assisted thermochemistry for the selective formation of high-quality wax.

The distinct reactivities of C-H versus C-C cleavage in wax refining were quantitatively demonstrated through the time-resolved H/C ratios of catalyst bed residues. Raw wax with dominant -CH_2_- units possessed a H/C ratio of 2.0, which underwent a rapid hydrogen abstraction under 15 h solar-driven refining, yielding shorter-chain olefinic wax, hydrogen gas, and a carbonaceous residue (H/C < 0.8) identified as coke (Supplementary Fig. 21). This demonstrates that the C-H activation preferentially generates olefinic intermediates, hydrogen, and coke precursors, which subsequently accelerates C-C cleavage to produce refined wax fractions. Raman spectroscopy of reacted nZVI catalysts confirmed surface coke formation through temporal evolution of vibrational bands: Fresh catalyst-wax mixtures exhibited characteristic -CH_2_ stretching modes at 2849 and 2882 cm^−1^ within 3 h (Supplementary Fig. 22)^48^, while post-reaction spectra showed complete disappearance of hydrocarbon signatures with emergence of graphitic (*E*_2g_, 1580 cm^−1^) and disordered carbon (*A*_1g_, 1300 cm^−1^) features^49^. Acid digestion of the residue in the catalyst bed isolated solid coke (Supplementary Fig. 23), enabling a quantification of the reaction stoichiometry for the solar-driven wax refining (Eq. 3).

3 $${{{\rm{C}}}}_{71}{{{\rm{H}}}}_{144}=	 1.3{{{\rm{C}}}}_{40}{{{\rm{H}}}}_{82}+0.7{{{\rm{H}}}}_{2}+0.004{{{\rm{CH}}}}_{4}+0.01{{{\rm{C}}}}_{2}{{{\rm{H}}}}_{6}+0.008{{{\rm{C}}}}_{2}{{{\rm{H}}}}_{4} \\ 	+0.003{{{\rm{C}}}}_{3}{{{\rm{H}}}}_{8}+0.002{{{\rm{C}}}}_{3}{{{\rm{H}}}}_{6}+11.3{{\rm{C}}}+residue_{mass\; loss}$$

Notably, the formation of coke on the nZVI surface did not produce detectable iron carbide or carbon phases (such as Fe_3_C, Fe_5_C_2_, C) according to XRD analysis. Catalytic longevity test revealed sustained activity of reacted catalyst throughout 11 successive runs, indicating that coke did not decrease the reactivity of the catalyst (Fig. 5c). Such a catalytic stability with coke under light differs with conventional thermocatalytic systems where coke induces to sharp deactivation of catalyst. This photostability aligns with recent photo-induced anti-coking effects^50,51^. Based on above stoichiometric analysis, the energy efficiency was calculated to be 0.1% under 4 W/cm^2^ irradiation (Supplementary Fig. 24, Supplementary Tables 7–15).

We then investigated the photothermal effect to understand the unique catalytic behavior under light. It is known that the light irradiation-induced heat is localized at micro-nanoscale, creating localized photothermal heating. Such local heating is especially high in plasmonic materials, as the case for nZVI containing Fe@Fe_2_O_3-*x*_ core-shell nanospheres, which showed a 7-fold electric field enhancement as revealed by the FDTD simulation (Fig. 5d). Referring to the boiling point of the refined wax (C_55_), the local temperature of nZVI under light was deduced as ~600 °C (Fig. 5e), significantly exceeding bulk temperature (280 °C) of the catalyst bed measured by the thermocouple. Replacing the local high temperature field with a bulk thermo-heating, the wax product reduced by 8–26% even at same (280 °C) or higher (450 and 600 °C) catalyst-bed temperature (Fig. 2b, Supplementary Fig. 25–29). Furthermore, sole sunlight photons promote wax’s C-H bonds activation and sole thermal heating promotes C-C cleavage, while their coupling results in increased C-H but decreased C-C cleavages, as revealed by light/dark activity test and DRIFTS study. Upon C-C/H cleavages to form olefinic wax fragment, coke and hydrogen, the hydrogen atom can be transferred to the iron oxide surface to reduce Fe^3+^ to Fe^2+^ and form hydroxyl (-OH) groups, which are reused for the hydrogenation of shorter-chain wax. The shorter-chain wax evaporation at photothermal hotspots (~600 °C) followed by condensation on cooler reactor walls (<100 °C), effectively prevents C-C over-cleavage and thus minimizes the formation of gas/liquid byproducts (Supplementary Figs. 30, 31).

Within such a reaction process, the remaining puzzle is the carbon-centered intermediate and associated molecular-level reaction mechanism. Having systematically reviewed the fundamentals for the deconstruction of waxy hydrocarbons, all possible reaction intermediates include 1) free radicals, 2) carbanions, and 3) carbocations, corresponding to three different reaction mechanism^52^. Since no carbon-centered radicals (•R) were detected by EPR using the spin trap of N-tertiary-butyl nitrone (PBN), we can safely exclude the random C-C thermolysis via free radical mechanism (Fig. 5f)^53^. Carbanion mechanism was also excluded due to the absence of strong bases, reducing agents, or highly polarized chemical environment in our system^54^. Therefore, the carbocation-mediated C-C scission mechanism is proposed for the nZVI-mediated wax refining. Theoretically, carbocation triggered by nZVI’s surface oxides (Fe_2_O_3-*x*_) is reasonable as the Lewis acid, Fe^2+^/Fe^3+^, dissociates C-C/H bond and accepts electron, while the neighboring lattice oxygen (O^2−^), a Lewis base, can bind and stabilize the resulting carbocation^55^. The remaining question is whether C-C scission occurs via an α- or β-scission pathway, distinguished by whether the bond cleaves adjacent to or two atoms away from the carbocation. Experimentally, the difference can be distinguished via the final gaseous product, as the α-scission resulted in majorly CH_4_ products while β-scission resulted in C_2+_ hydrocarbons. As seen from the activity test, conventional thermochemistry produced dominantly gaseous CH_4_, while our light-assisted localized heating process generated mainly non-CH_4_ gases, suggesting a carbocation-mediated β-scission mechanism over irradiated nZVI (Fig. 5g).

**Fig. 6:**
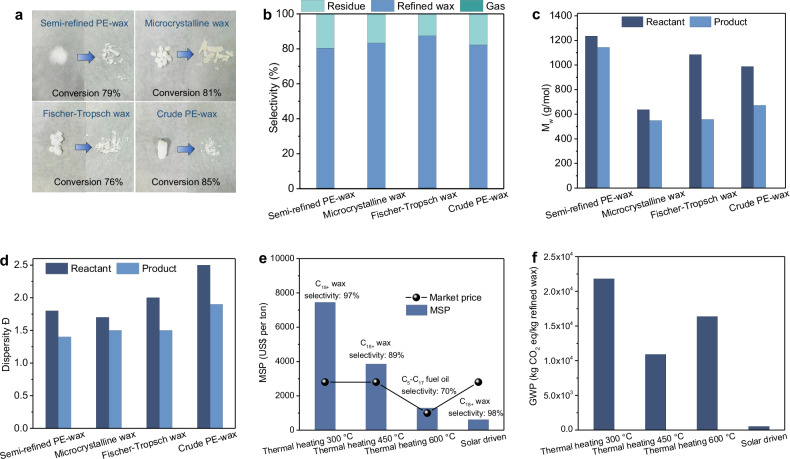
Universality of selective solar refining across different wax types and corresponding TEA and LCA. **a** Digital photos of semi-refined PE-wax, microcrystalline wax, Fischer-Tropsch wax and crude PE-wax and corresponding wax products after solar-driven refining. **b** Products selectivity after 5 h reaction under 4 W/cm^2^ sunlight irradiation. **c** Molecular weight distribution and **d** dispersity of semi-refined PE-wax, microcrystalline wax, Fischer-Tropsch wax, crude PE-wax and their refined wax products based on HT-GPC measurements. **e** MSP of main products (thermal heating 300 °C, 450 °C and solar driven: C_18+_ wax with 97%, 89% and 98% selectivity, respectively; thermal heating 600 °C: C_5_-C_17_ fuel oil with 70% selectivity) obtained from wax refining, in comparison with their average market price. **f** GWP of the thermal heating 300 °C, 450 °C, 600 °C and solar-driven wax refining strategies.

Technology validation across major wax classes (semi-refined PE-wax, microcrystalline wax, Fischer-Tropsch wax, crude PE-wax) demonstrated universal applicability (Fig. 6a). At 4.0 W/cm^2^ of illumination, this solar wax refining strategy achieved >75% conversion of all raw waxes and >80% selectivity for high-grade narrowly distributed refined wax (*Ð* < 1.5) within 5 h, with the *M*_*w*_ of refined wax slightly lower than that of raw wax (500–1300 g/mol; Fig. 6b–d, Supplementary Fig. 32 and Supplementary Table 16, 17). Structural analyses via FT-IR and GC-MS confirmed the retention of linear *n*-alkane frameworks in all refined products, without any detectable isomerization, cyclization, or aromatization (Supplementary Fig. 33-35). For the refined microcrystalline wax and Fischer-Tropsch, the olefinic proton ratios were 0.44% and 0.55% respectively, indicating that the purity of the refined wax products achieved 99% (Supplementary Fig. 36). In 10 cycles, the conversion for both Fischer-Tropsch wax and microcrystalline wax were above 70%, suggesting the stability of the nZVI material (Supplementary Fig. 37). Furthermore, the dispersity of all refined waxes was much smaller than that obtained from HDPE pyrolysis waxes (*Ð* < 9.9) via conventional thermochemistry industry^8^, highlighting the wide applicability and transformative potential for the solar-driven wax refining.

Following the successful laboratory test, we upscaled an outdoor reactor working under sunlight to verify the scalability of our solar wax refining. 5 g raw wax (*Ð* = 2.5, *M*_*w*_ = 988 g/mol, C_71_ equivalent) and 1 g nZVI were mixed and loaded into a 300 mL reactor, equipped with a 0.5*0.5 m^2^ Fresnel lens. The light intensity reached to the reactor was 3.6–4.3 W/cm^2^ in Shanghai between 13:35 and 15:35 on July 9, 2025 (Supplementary Fig. 38). After 2 h of reaction, the mass yields were: gas products (C_1_-C_4_ and H_2_) ~ 0.43%, liquid compound (C_5_–C_17_) ~ 0.7%, target solid wax (C_18+_) ~ 62%, and solid residue ~20% (Supplementary Fig. 39). The selectivity towards the valuable wax fraction reached ~98%. These results unequivocally demonstrate that our solar wax refining strategy is not only efficient but also readily deployable when powered by sunlight. The elimination of complex separation units and solvent recovery systems, coupled with the earth-abundant catalyst, offers significant scaling and cost advantages over conventional, electricity-intensive thermochemical processes.

To evaluate the industrial viability and environmental footprint of the solar-driven catalytic wax refining process, we conducted a rigorous techno-economic analysis (TEA) and life cycle assessment (LCA) benchmarked against conventional thermal pathways operated under 300, 450, and 600 °C over nZVI catalyst (Supplementary Fig. 40). Our TEA models a continuous plant with an annual capacity of 10,000 tonnes of raw wax, employing a discounted cash flow method under standard financial assumptions (30-year lifetime, 10% internal rate of return). The analysis incorporates robust operational parameters, including a catalyst lifetime of 100 cycles (based on experimental stability tests) and a thermal efficiency of 60% for the solar thermal system. The LCA was performed using SimaPro with the ReCiPe 2016 (H) method and the Ecoinvent 3.0 database, ensuring a comprehensive cradle-to-gate inventory. The TEA results are conclusive: the minimum selling price (MSP) for refined wax produced via the solar pathway is US$612 per tonne. This is 3- to 12-fold lower than the MSPs required for the thermal pathways (US$1285–7450 per tonne), underscoring a decisive economic advantage (Fig. 6e, Supplementary Fig. 41-45, Supplementary Table 18-21). This cost competitiveness is primarily attributable to the elimination of fuel costs and the effective amortization of catalyst expenses through prolonged recycling.

Environmentally, the nZVI-enabled solar pathway demonstrates a profound reduction in carbon emissions compared to existing thermochemical methods. Its global warming potential (GWP) is merely 516 kg CO_2_-eq per tonne of refined wax, which is a significant overestimate due to the LCA adopted an enlarged otherwise non-calculatable carbon emission associated with the precursor chemical NaBH_4_. Nevertheless, the GWP of solar wax refining is only 2–5% that for the nZVI-enabled conventional thermochemical pathways (16,358–21,815 kg CO_2_-eq per tonne; Fig. 6f). This translates into a net reduction of 10–21 tonnes of CO_2_-eq per tonne of product for our solar wax refinery. Sensitivity analysis confirms that the excellent performance of the solar pathway remains robust across variations in key parameters like capital investment and catalyst lifetime (Supplementary Table 20).

## Discussion

We have demonstrated a solar-wax-refining strategy enabled by an earth-abundant Fe@Fe_2_O_3-*x*_ catalyst that selectively refines low-grade commercial waxes (microcrystalline, Fischer-Tropsch, crude and semi-refined PE waxes) into high-grade waxes with a narrow distribution of carbon chain length, driven by solar energy. The refining process achieved more than 80% selectivity and 0.10% solar-to-chemical energy efficiency under optimized conditions. During the process, photon absorption by Fe@Fe_2_O_3-*x*_ results in the plasmonic effect that weakens and dissociates C-H bonds into olefinic wax and hydrogen atoms via a non-radical pathway, and then photothermal heating directs energy toward hydrogen-assisted C-C bond cleavage and rapid desorption of resultant shorter-chain wax from the nZVI surface, halting secondary C-C cleavage and suppressing gas/liquid by-product formation. This work exemplifies a solar refinery that break similar C-C bonds in hydrocarbons selectively and efficiently. Furthermore, our integrated TEA-LCA assessments unequivocally indicate the solar-driven wax refining as a sustainable and efficient pathway than current thermochemical methods.

## Methods

### The preparation of nZVI catalyst

NaBH_4_ solution (200 mL, concentration: 0.04 mol/L) was dropwise added to the FeCl_3_•6H_2_O solution (500 mL, concentration: 0.01 mol/L). The solution was then aged for 2 hours to obtain a clear solution with floating black precipitates. The black precipitates were separated and washed with deionized water several times, followed by drying under Ar and infrared light irradiation, and finally collected and stored in anaerobic conditions, termed nZVI.

### Solar-driven wax refining tests

The reaction was carried out in a 50 mL batch reactor filled with Argon under atmospheric pressure. In the optimized experimental procedure, 100 mg of catalyst powder and 100 mg of raw wax powder were physically mixed by shaking and then the mixture was loaded into the batch reactor. Because of nZVI’s black color, the mixed powder appears black as shown in Supplementary Fig. 3a. The reaction proceeds without external heating. Reaction timing started when the 300 W xenon lamp equipped with focus lens (Microsolar 300, Beijing Perfectlight Technology) was turned on. The raw wax rapidly melted after the light on induced high temperature, forming a solid-liquid mixed phase. Driven by the localized high temperature (>600 °C) of nZVI, the product wax vaporizes and separates from the catalyst. Subsequent wax condensation occurred on the cool reactor interior surfaces (<100 °C), realizing wax refining and separation simultaneously. As shown in Supplementary Fig. 3c, some waxes condensed on the window of the reactor, forming a “O-ring” pattern and leaving the center where the light penetrate through unaffected. Furthermore, some waxes condensed on the interior wall and the bottom of the reactor (outside the catalyst bed). These condensed waxes were mainly collected via stainless-steel spatula, with residues collected by solvent (n-hexane) washing followed by wax recovery via vaporizing the n-hexane solvent. The catalyst bed after the reaction contains a small portion of wax residue and dominantly spent catalyst, which we did not separate them. The mass of wax residue in the catalyst bed can be calculated by the mass difference before and after the reaction. Gas products were sampled with GC syringe, and analyzed by GC-FID and GC-TCD (Tet Instrument GC-2030). The yield, selectivity and content of certain components in the products were calculated by following equations (Eqs. 4–7).

4 $${{\rm{C}}}{{\rm{on}}}{{\rm{version}}}=\frac{{m}_{\,{{\rm{raw\; wax}}}}-{m}_{\,{{\rm{residue}}}}}{{m}_{\,{{\rm{raw\; wax}}}}}\times 100\%$$

5 $${{\rm{Yield}}}=\frac{{m}_{{{\rm{product}}}}}{{m}_{\,{{\rm{raw\; wax}}}}}\times 100\%$$

6 $${{\rm{Wax\; s}}}{{\rm{ele}}}{{\rm{ctivity}}}=\frac{{m}_{{{\rm{wax}}}}}{{m}_{{{\rm{residue}}}}+{m}_{{{\rm{wax}}}}\,{+{m}}_{{{\rm{gas}}}}}\times 100\%$$

7 $${C}_{18+}{{\rm{content}}}=\frac{{m}_{C18+}}{{m}_{C5-C17}+{m}_{C18+}\,}\times 100\%$$

where *m*_*raw wax*_*, m*_*residue*_*, m*_*refined wax*_*, m*_*gas*_ refer to masses of raw wax, residue, refined wax, and gas product, respectively. *m*_*C18+*_ and *m*_*C5-C17*_ are masses of C_18+_ and C_5_-C_17_ hydrocarbons in refined wax, respectively.

### Kinetics and activation energy calculations

The reaction rate and rate constant were calculated by the following equations (Eqs. 8, 9).

8 $${r}_{p}=-\frac{{d}^{{m}_{{residue}}}/{m}_{0}}{{dt}}$$

9 $${{\mathrm{ln}}}\frac{{m}_{{{\rm{residue}}}}}{{m}_{0}}=-{kt}$$

where *r*_*p*_ is the intrinsic reaction rate, *m*_*residue*_ and *m*_0_ represent the residual and initial mass of the raw wax in the catalyst bed, respectively, while *k* represents the intrinsic rate constant. The activation energy was calculated by the following equations (Eq. 10).

10 $${{\mathrm{ln}}}k={{\mathrm{ln}}}A-\frac{{E}_{a}}{{RT}}$$

where *R, T, E*_*a*_*, A* are gas constant (8.314 J mol^−1^ K^−1^), temperature (K), activation energy (kJ/mol), and pre-exponential factor, respectively.

### Catalyst characterization

The SEM and STEM measurements were carried out on TESCAN MIRA LMS and Titan G2 60-300 electron microscopes, respectively. Crystalline phases of the nZVI catalyst and waxes were detected by powder XRD recorded on a Bruker D8 Advance X-ray diffractometer with Cu Kα radiation (*λ* = 1.54178 Å) at 40 kV and 40 mA. Chemical environments of nZVI catalyst were analyzed by X-ray photo–electron spectroscopy (XPS, Thermo Scientific K-Alpha). All reported binding energies were calibrated using the C *1s* peak (284.8 eV). UV–Vis diffuse reflectance spectroscopy (DRS) measurement were conducted on a Shimadzu UV-2600 spectrometer. The resolution was 1.0 nm over the range 200–1400 nm and the slit width was 5 nm, with the background spectrum collected based on an optical-grade BaSO_4_ specimen. The XAFS spectra of Fe K-edge were collected in transmission mode on a commercial Laboratory-Based XAFS spectrometer (Table XAFS-500A, Specreation Instruments Co., Ltd). An X-ray tube was used to generate X-rays, and the voltage and current were set to 20 kV and 20 mA. The Ge (620) spherically bent crystal analyzers with a radius of curvature of 500 mm and the R250 mm Rowland circle were used to provide a monochromatized X-ray beam.

### Analyses of wax refining products

GC-FID/TCD: Hydrocarbons (C_1_–C_4_) and H_2_ in the gas products were qualitative and quantitative on a Tet Instrument GC-2030 gas chromatograph with an Al_2_O_3_ capillary column (30 m × 0.53 mm × 20 µm) and a 5 A packed column, equipped with a flame ionization detector and a thermal conductivity detector. The injector temperature was 220 °C, and the flame ionization detector and the thermal conductivity detector temperatures were 200 and 70 °C, respectively. The oven temperature was 70 °C and the TCD current was 70 mA.

GC-MS: Hydrocarbons (C_5_-C_28_) in the wax were identified on a Thermo Scientific Trace ISQ 1300 gas chromatograph equipped with a TG-5MS capillary column (30 m × 0.25 mm × 0.25 µm) and a mass spectrometer. The inlet and ion source temperatures were 200 and 270 °C, respectively. The oven temperature program was: 50 °C holding for 2 min, ramping to 280 °C at a rate of 10 °C/min and holding for 5 min. The carrier gas flow rate was 1.0 mL/min (He) with spitless method. The mass (m/z) range was 40–450, and the solvent delay was 3.5 min.

GC-FID: Hydrocarbons (C_5_-C_28_) in the wax were quantified on a Thermo Scientific Trace 1300 gas chromatograph equipped with a TG-5SILMS capillary column (30 m × 0.25 mm × 0.25 µm) and a flame ionization detector. The inlet temperature was 270 °C. The oven temperature program was: 50 °C (hold 2 min), ramping to 280 °C at 10 °C/min (hold 5 min). The peak positions and response factors of linear alkanes were obtained using standard solutions of n-alkanes (C_8_-C_18_) with the 1,3,5-tri-tertbutyl benzene (concentration: 25 mg/L) as an internal standard. All the relative carbon response factors were assumed to be 1.0 in GC-FID analyses.

HT-GPC: Molecular weight distributions of waxes were analyzed on EcoSEC HLC-8321 gel permeation chromatograph. Samples were dissolved in 1,2-dichlorobenzene by heating at 145 °C for at least 1 h. The molecular weight response was calibrated by the polystyrene standards (500–548,0000 g/mol).

^1^H NMR: The ^1^H NMR spectra were recorded in the Varian NMR Systems 600 MHz spectrometer. Reported chemical shifts (δ, ppm) were calibrated using tetramethylsilane (TMS). NMR specimen was prepared by dissolving wax product in 1 mL of CDCl_3_ (D 99.8%, TMS 0.003%) followed by filtering to remove insolubles. Protons of different chemical environments can be identified within the wax molecule. Furthermore, the integration of proton peak areas and identification of corresponding chemical structures enable detailed quantification as Eq. 11.

11 $${{\rm{Proton\; content}}}=\,\frac{{A}_{i}}{\sum {A}_{i}}$$

where *A*_*i*_ represents the peak area of protons in a certain chemical environment, which includes the protons of -CH_3_, -CH_2_ and -HC = CH_1-2_.

Ex-situ FTIR: FTIR spectra of wax were recorded on a Tensor 27 spectrometer. Samples (2 mg) were ground with a mortar and pestle, mixed with KBr (200 mg), and then patterned into 15 mm-in-diameter pellets under a pressure of 4 tons.

XPS: Chemical environments of wax and nZVI were analyzed by a Thermo Scientific X-ray photo-electron spectroscopy. All reported binding energies were calibrated using the C *1s* peak (284.8 eV).

DRS: Light absorbance of all samples was measured on a Shimadzu UV-2600 spectrometer. The resolution was 1.0 nm over the range 200–1400 nm and the slit width was 5 nm, with the background spectrum collected based on an optical-grade BaSO_4_ specimen.

TEA and LCA: Techno-economic feasibilities of catalytic wax refining were conducted using the Discounted Cash Flow (DCF) model, which focuses on the financial objective of “determining the Minimum Selling Price (MSP) of the main product to achieve an NPV = 0 and an IRR = 10%”. First, the basic project settings (such as general parameters including a 30-year project lifecycle, 90% operating rate, 21% tax rate, and a total investment of USD 5.25 million) were defined. Second, investment costs (fixed investment + working capital) and operating costs (variable costs include raw materials, catalysts, and energy, while fixed costs include labor and maintenance) were analyzed. Third, the long-term cash flow statement was used to iteratively solve for the MSP. Finally, risk points were identified through sensitivity analysis and comparison with different technical routes.

## Supplementary information

Supplementary Information

Transparent Peer Review file

## Source data

Source data

## Data Availability

All data that support the findings of this study are present in the paper and the Supplementary Information, and are available from the corresponding author upon request. Source data are deposited in the Figshare database [10.6084/m9.figshare.31315522]^56^. Source data are provided with this paper.
