# Supplementary material for: Selective solar wax refining with nanoscale zero-valent iron
Source: Nat Commun. 2026 Mar 30;17:7555. doi: 10.1038/s41467-026-71010-0 (PMC13415555; doi:10.1038/s41467-026-71010-0)
Supplement: Supplementary file 1 — Supplementary Information [file 41467_2026_71010_MOESM1_ESM.pdf]

Supplementary Information for

Selective Solar Wax Refining with Nanoscale Zero-Valent Iron

Yifei Sun[1], Chengliang Mao[2*], Yunjie Zou[3], Yuqing Hu[1], Di Yang[4], Junzhou Xu[2], Haopeng Pei[2], Yanbiao Shi[5], Zipeng Chen[1], Wendong Wei[2], Zhihui Ai[1*], Lizhi Zhang[2*]

[1]State Key Laboratory of Green Pesticide, Engineering Research Center of Photoenergy Utilization for Pollution Control and Carbon Reduction, Ministry of Education, College of Chemistry, Central China Normal University, Wuhan, P. R. China.

[2]State Key Laboratory of Green Papermaking and Resource Recycling, Shanghai Engineering Research Center of Solid Waste Treatment and Resource Recovery, School of Environmental Science and Engineering, National observation and Research Station of Erhai Lake Ecosystem in Yunnan, Yunnan Dali Research Institute, Shanghai Jiao Tong University, Shanghai, P. R. China.

[3]State Key Laboratory for Pollution Control and Resource Reuse, College of Environmental Science and Engineering, Tongji University, Shanghai, P. R. China.

[4]School of Energy and Materials, Shanghai Polytechnic University, Shanghai, P. R. China.

[5]State Key Laboratory of Hydraulics and Mountain River Engineering, College of Architecture and Environment, Sichuan University, Chengdu, P. R. China.

*Correspondence to: chengliang.mao@sjtu.edu.cn; jennifer.ai@ccnu.edu.cn; zhanglizhi@sjtu.edu.cn

Energy efficiency calculation

Solar wax refining involves simultaneous C(sp^3)-C(sp^3) and C(sp^3)-H bond cleavage and rearrangement. Due to this molecular-level mechanism, conventional quantum efficiency calculations become inadequate for reliable energy assessment. We therefore employ comprehensive energy efficiency (η) as a more robust performance metric, defined as:

$$\eta = \frac{E_{\mathrm{re}}}{E_{\mathrm{input}}} \tag{S1}$$

where E_{re} represents the total energy required for the reaction. The E_{input} denotes the photon energy absorbed by the catalyst, which is estimated by the DRS spectrum through the following equation:

$$E_{\mathrm{input}} = \int_{200\,nm}^{1400\,nm} W_i \times Abs \times S \times Time \tag{S2}$$

where the W_i is the optical power density which obtained from the optical power meter, *Abs* is the absorbance of catalyst at a certain wavelength, *S* is the irradiation area and *Time* is the irradiation time.

$$E_{\mathrm{re}} = Q_c + Q_w + \Delta_r H \tag{S3}$$

where Q_c and Q_w correspond to the thermal energy absorbed by nZVI nanoparticles and raw wax, respectively, during heating from ambient temperature (T_1) to the nanoscale surface reaction temperature (T_3), while $\Delta_r H$ represents the reaction enthalpy at T_3.

For heat capacity calculations, we employed:

$$Q_c = n_{Fe} \int_{T_1}^{T_3} C_{p-Fe}\ dT \tag{S4}$$

where C_p (cal · mol^{-1} · K^{-1}) and *n* (mol) are the pressure-constant molar heat capacity and the amount mole of components[1]. C_p is a function of temperature:

$$C_{p-Fe}(cal \cdot mol^{-1} \cdot K^{-1}) = 4.13 + 0.00638T \quad \text{(S5)}$$

The heat absorbed by PE-wax includes solid wax heat absorption from T_1 to melting point (T_m), phase change enthalpy ($\Delta_{\text{fusion}}H$) and liquid wax heat absorption from T_m to T_3. The T_m of raw wax (about 379 K, n-$C_{71}H_{144}$) was estimated by fitting the linear-alkane melting point-carbon number distribution diagram (Supplementary Fig. 46). The pressure-constant molar heat capacity of solid wax (C_{p-s}) and liquid wax (C_{p-l}) is estimated via Martin G. Broadhurst's method[2]. Gaseous hydrocarbon products (C_{p-g}) is estimated via Sidney W. Benson's group increment theory[3].

$$Q_w = n_{\text{wax}} \int_{T_1}^{T_m} C_{p-s}\, dT + n_{\text{wax}} \int_{T_m}^{T_3} C_{p-l}\, dT + n_{\text{wax}} \Delta H_{\text{fusion}} \quad \text{(S6)}$$

$$C_{p-l}(cal \cdot mol^{-1} \cdot K^{-1}) = 5 + 0.00818T \quad \text{(S7)}$$

$$C_{p-s}(cal \cdot mol^{-1} \cdot K^{-1}) = 0.0139T + 0.916 + \frac{223080}{T^2} e^{-3.32172*(414.3-T)/T} \quad \text{(S8)}$$

$$\Delta_{fusion}H(cal \cdot mol^{-1} \cdot K^{-1}) = -0.00186T^2 + 4.84T - 39 - 162.1e^{-3.32(414.3-T)/T} \text{(S9)}$$

The reaction enthalpy ($\Delta_r H$) was calculated by this designed thermodynamic paths (Supplementary Fig. 24), incorporating standard formation enthalpies ($\Delta_f H$) of all species and the enthalpy of molar formation. The standard molar enthalpy of formation for H_2 is 0.

$$\Delta_r H^{\Theta} = \sum n_{\text{products}} \Delta_f\, H_{\text{products}} - \sum n_{\text{substrate}} \Delta_f\, H_{\text{substrate}} \quad \text{(S10)}$$

$$\Delta_r H = q_1 + \Delta_{\text{vap}}H_1 + q_2 + \Delta_r H^{\Theta} + q_3 + q_4 + q_5 + \Delta_{\text{vap}}H_1 + q_6 - \Delta_{\text{vap}}H_2 - \Delta_{\text{fusion}}H_2 \quad \text{(S11)}$$

In this designed thermodynamic process, liquid raw wax absorbs heat q_1 as it is heated from T_3 to its boiling point T_b (939 K, n-$C_{71}H_{144}$). The corresponding vaporization enthalpy $\Delta_{\text{vap}}H_1$ at T_b was determined by fitting the linear alkane boiling point-carbon number correlation. As the resulting gaseous raw wax cools from T_b to T_1,

it releases heat ($-q_2$). The reaction enthalpy $\Delta_r H^\Theta$ was derived from standard formation enthalpies ($\Delta_f H$). Meanwhile, coke absorbs heat q_3 when heated from T_1 to T_3, while H_2 requires heat q_4 to reach the reaction temperature (T_2) from T_1. The process further involves gaseous refined wax absorbing heat q_5 as it is heated from T_1 to its boiling point T_{b2} (873 K, n-$C_{55}H_{112}$), where $\Delta_{vap}H_2$ represents its vaporization enthalpy. Finally, liquid refined wax absorbs heat q_6 when cooled from T_{b2} to T_1, with its melting enthalpy $\Delta_{fusion}H_2$ at T_2 estimated using Martin G. Broadhurst's method[2].

Phase transitions at characteristic melting and boiling points were treated as $\Delta G = 0$ processes. The corresponding vaporization enthalpies ($\Delta_{vap}H_1$ and $\Delta_{vap}H_2$) were calculated through:

$$\Delta H = T\Delta S \tag{S12}$$

The entropies of the raw and refined wax in the gaseous state are estimated by the Benson group increments method. The entropy of the raw and refined wax of the liquid state (S_l) was estimated by Martin G. Broadhurst's method. The entropy change of refined wax solidification is directly calculated by Martin G. Broadhurst's method[2]. Other endothermic or exothermic processes ($q_{1\text{-}6}$) without phase transitions are estimated by corresponding heat capacities[1].

$$q_{1-6} = n \int C_p \; dT \tag{S13}$$

$$C_{P-H2}(cal \cdot mol^{-1} \cdot K^{-1}) = 6.62 + 0.00081T \tag{S14}$$

$$S_l(cal \cdot mol^{-1} \cdot K^{-1}) = 0.00818T + 5lnT - 23.26 \tag{S15}$$

Where, the heat capacity of coke ($C_{p\text{-}coke}$) is 0.359 cal · K^{-1} · g^{-1} at temperature ranging from 294 K to 1073 K. The molar entropy at different temperatures is calculated by the following formula[1].

$$S_g = S_g^{\Theta} + \int C_p T \ dT \tag{S16}$$

$$\Delta S_{l-g} = S_g - S_l \tag{S17}$$

Supplementary Figures

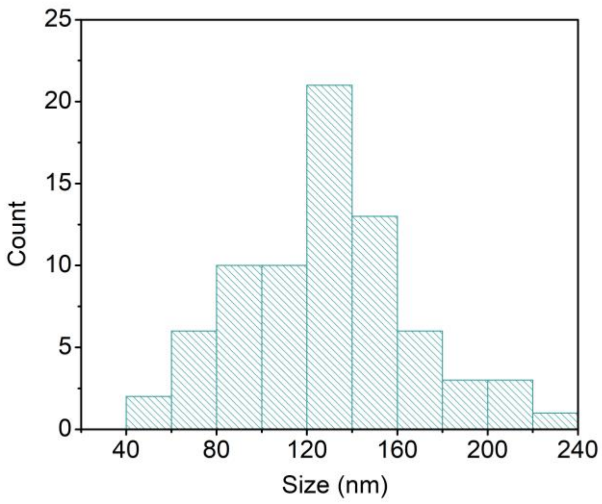

Supplementary Fig. 1 Size distribution of nZVI nanospheres.

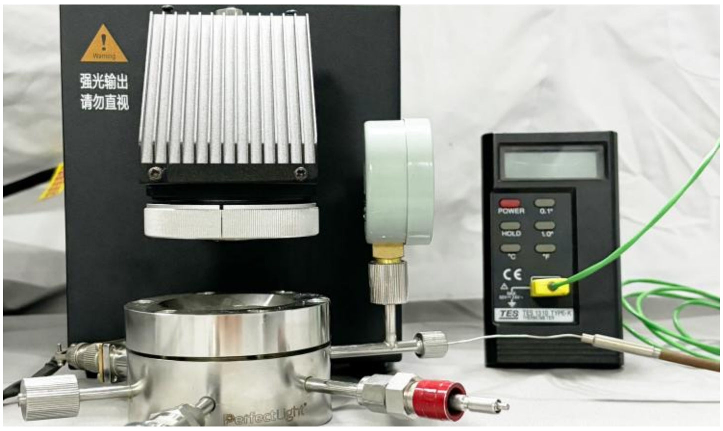

Supplementary Fig. 2 Digital photo of solar wax refining reactor.

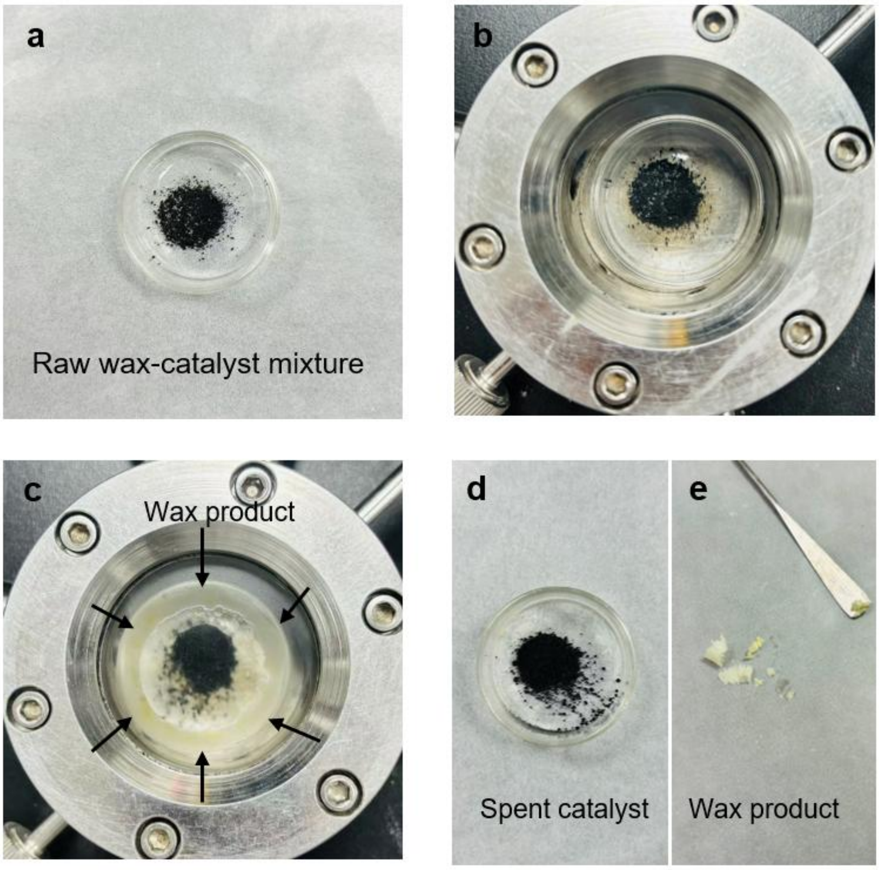

Supplementary Fig. 3 Digital photos showing the procedure of catalyst-wax seperation. (a) Raw wax mixed with catalyst, and (b) the reactor loaded with catalsyt and raw wax before reaction. (c) Wax products condensed on a quartz window after solar wax refining. (d) Spent catalyst, and (e) wax products collected from the window and walls using a stainless-steel spatula.

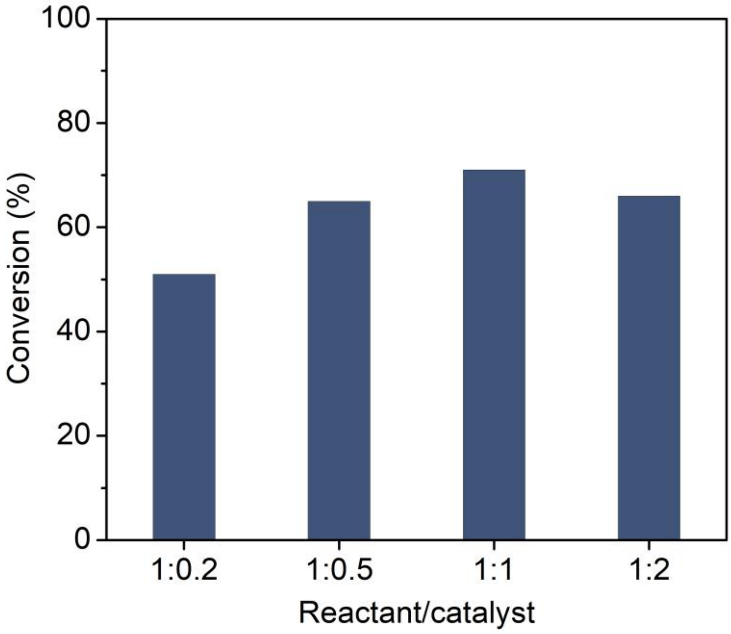

Supplementary Fig. 4 The influence of wax/catalyst ratio to the refining performance. Wax conversions obtained with reactant-to-catalyst ratios of 1 : 0.2, 1 : 0.5, 1 : 1 and 1 : 2 under 4.0 W/cm^2. Reactant: raw wax 100 mg; Catalyst: nZVI 20, 50, 100, and 200 mg.

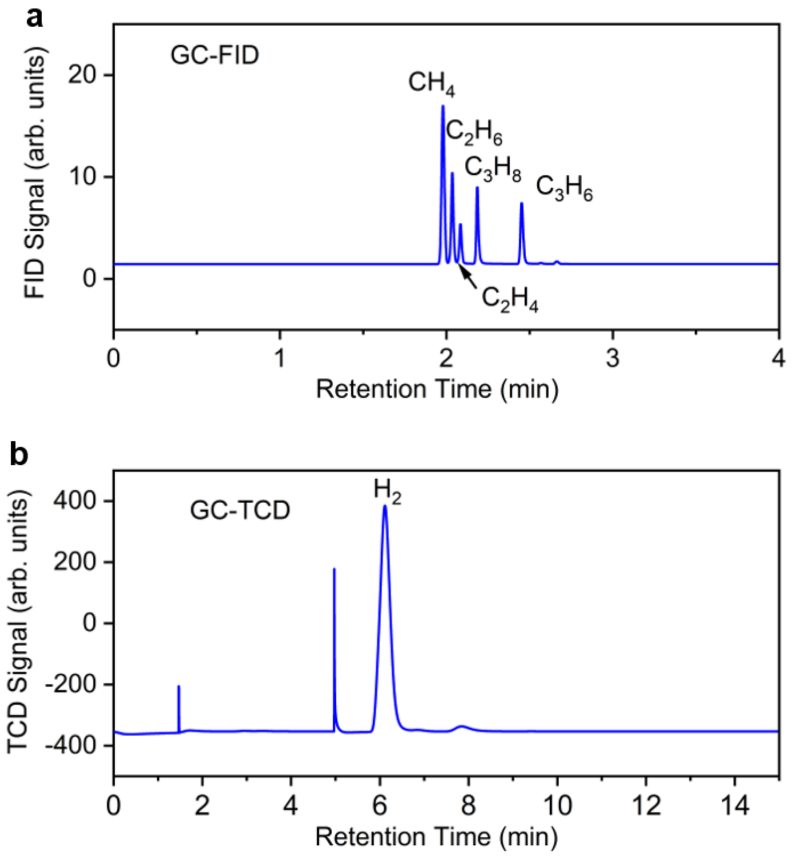

Supplementary Fig. 5 GC detection of gaseous products in the solar wax refining.

(a) GC-FID, and (b) GC-TCD spectra of C_1-C_4 and H_2 products after 15 h reaction under light intensity of 3.1 W/cm^2 during solar wax refining.

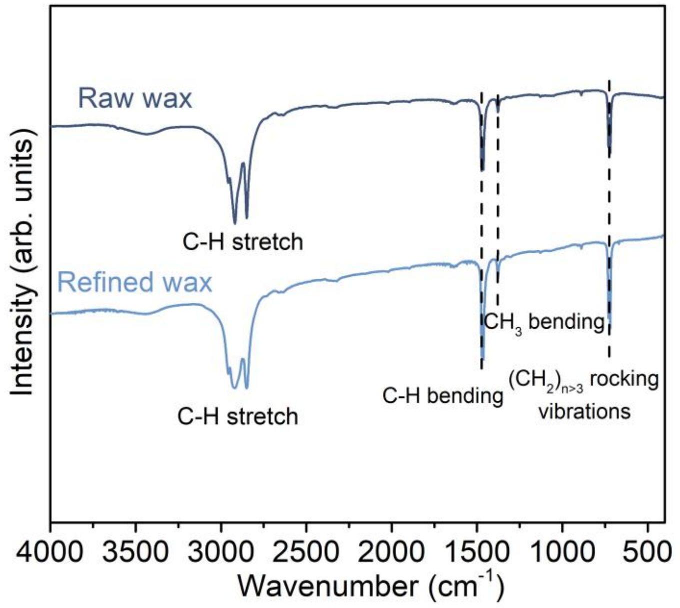

Supplementary Fig. 6 FTIR spectra of raw and refined waxes.

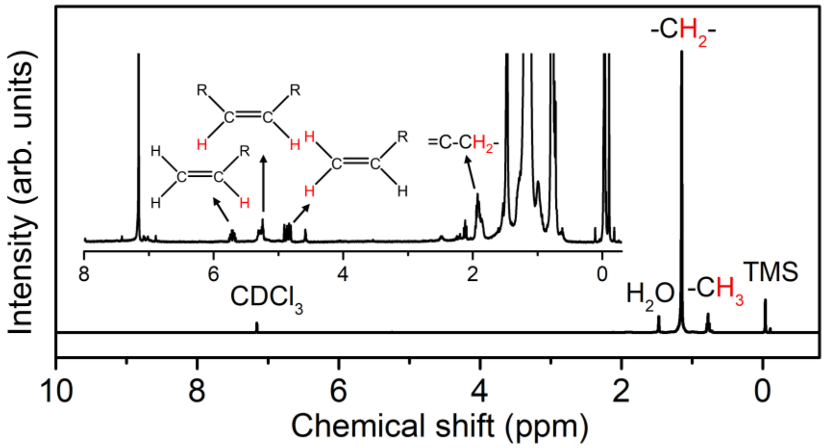

Supplementary Fig. 7 ^{1}H NMR spectrum of the wax product obtained after 15 h reaction under 3.1 W/cm^2 light intensity. Solvent: $CDCl_3$.

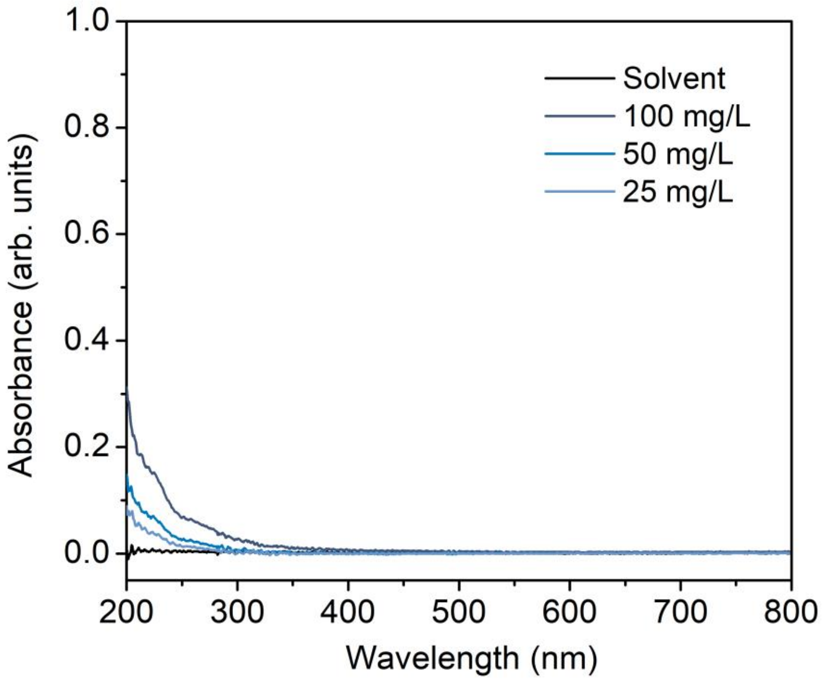

Supplementary Fig. 8 UV-Vis spectra of wax products dissolved in n-hexane.

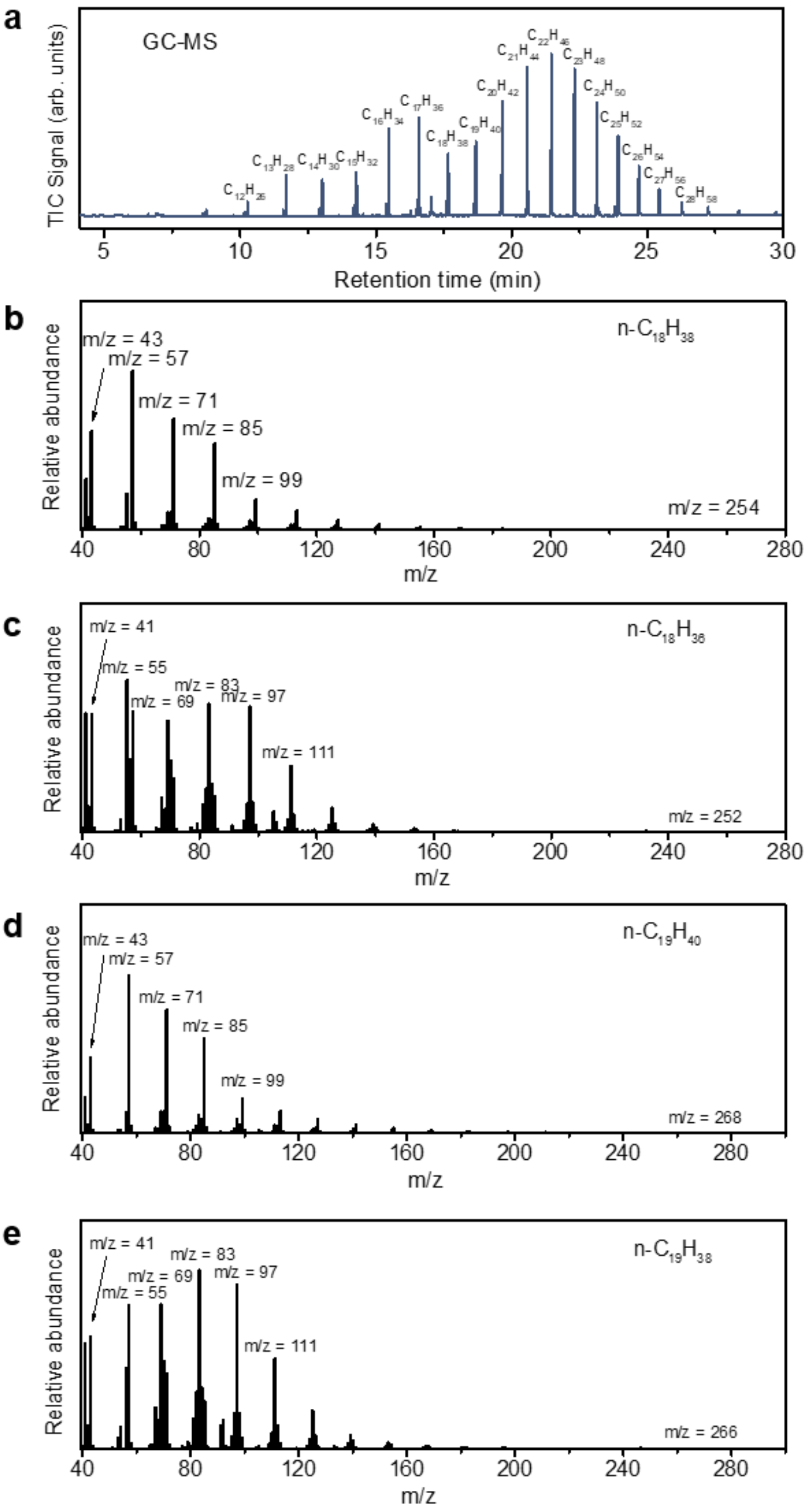

Supplementary Fig. 9 GC-MS spectrum of the refined wax. (a) GC-MS spectrum of the wax product dissolved in n-hexane. (b-e) Selected MS mass patterns for n-$C_{18}H_{38}$, n-$C_{18}H_{36}$, n-$C_{19}H_{40}$, and n-$C_{19}H_{38}$.

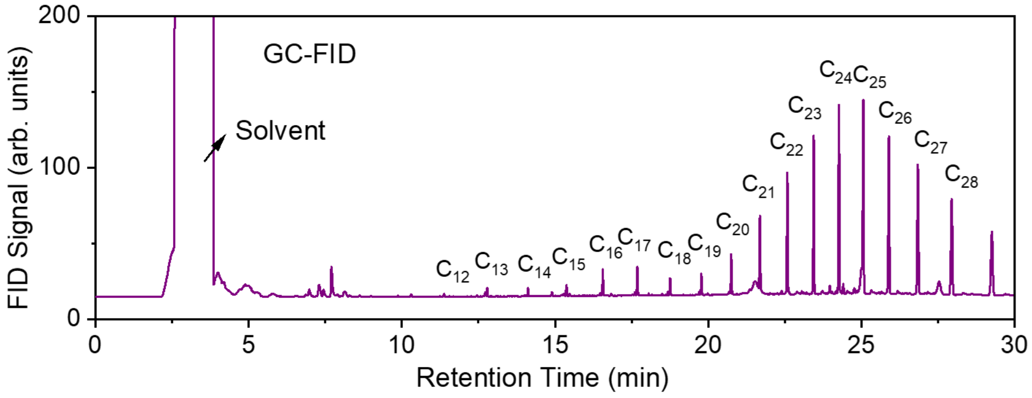

Supplementary Fig. 10 GC-FID spectrum of the wax product dissolved in n-hexane.

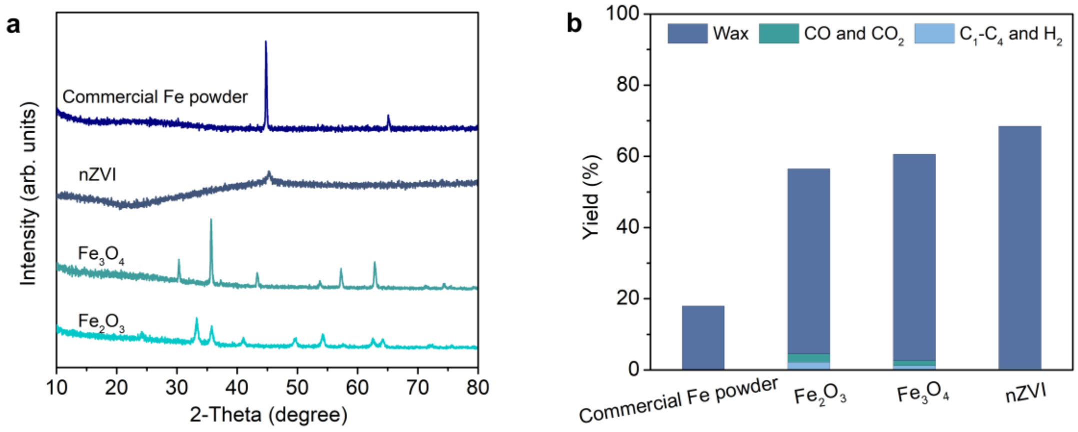

Supplementary Fig. 11 Optimization of the catalyst for solar wax refining. (a) XRD patterns of commercial Fe powder, Fe_2O_3, Fe_3O_4 and nZVI. (b) The yield of products after 3 h reaction over commercial Fe powder, Fe_2O_3, Fe_3O_4 and nZVI under 4 W/cm^2.

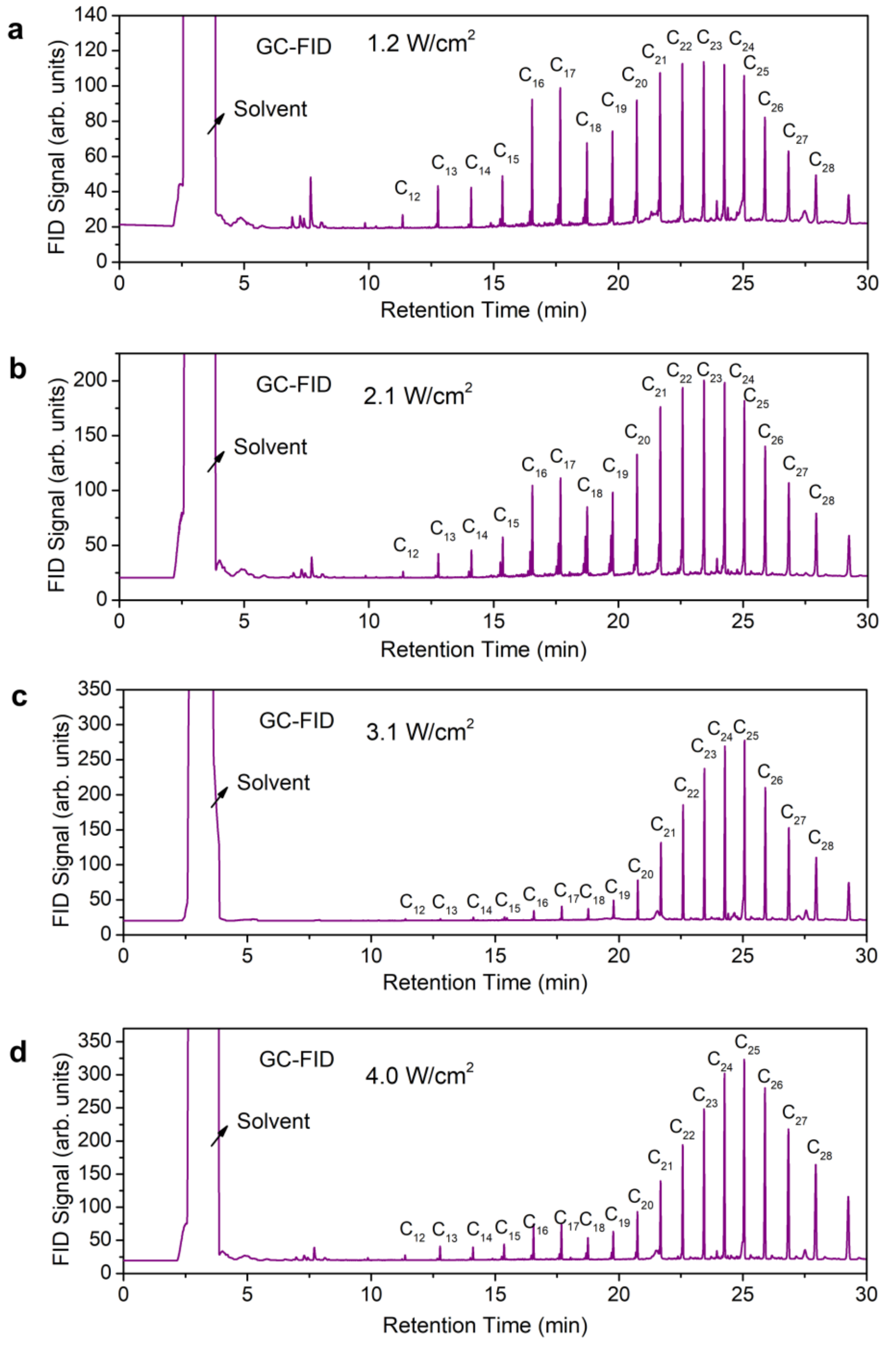

Supplementary Fig. 12 GC-FID spectra of wax products obtained under different light intensities. (a) 1.2, (b) 2.1, (c) 3.1, and (d) 4.0 W/cm^2.

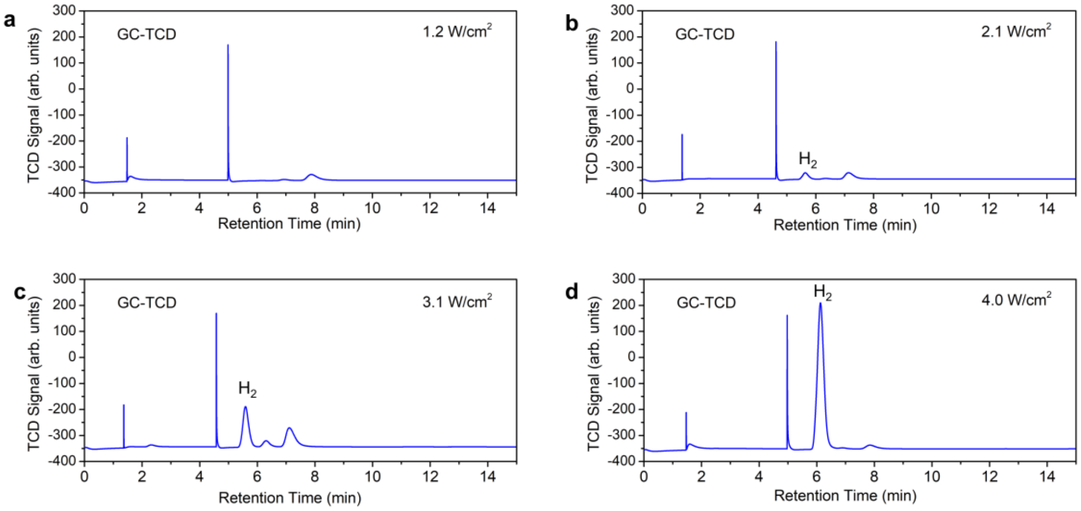

Supplementary Fig. 13 GC-TCD spectra for gas products obtained after 3 h solar wax refining reaction under different light intensities. (a) 1.2, (b) 2.1, (c) 3.1, and (d) 4.0 W/cm^2.

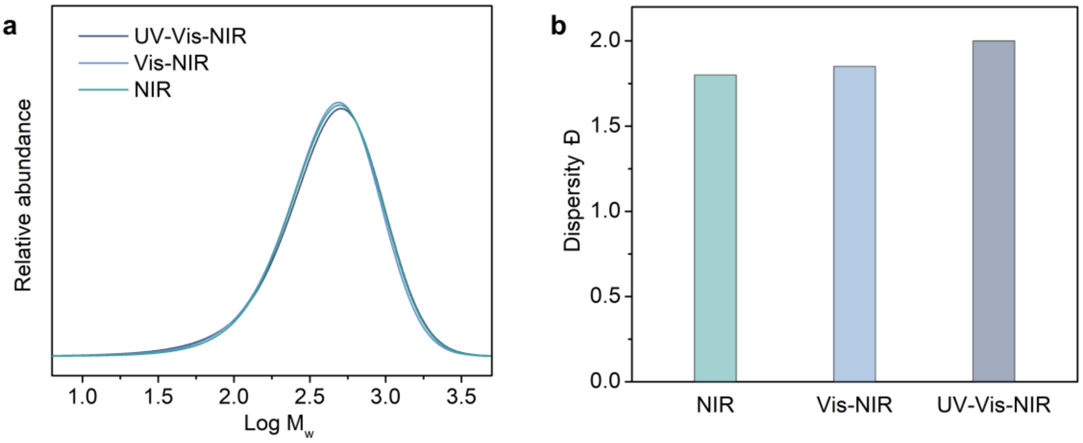

Supplementary Fig. 14 Wax products obtained under different light wavelengths. (a) Molecular weight distribution and (b) dispersity of product wax under UV-Vis-NIR, Vis-NIR and NIR irradiation, 280 °C after 3 h reaction, according to HT-GPC measurements using 1,2-dichlorobenzene as the solvent. Under the same temperature, though photons with higher energy activated more C-C and C-H bonds in the raw wax, refined waxes obtained under different light wavelengths possessed similar molecular weight and dispersity, suggesting that the cracking and evaporative separation processes were dominated by photothermal heating.

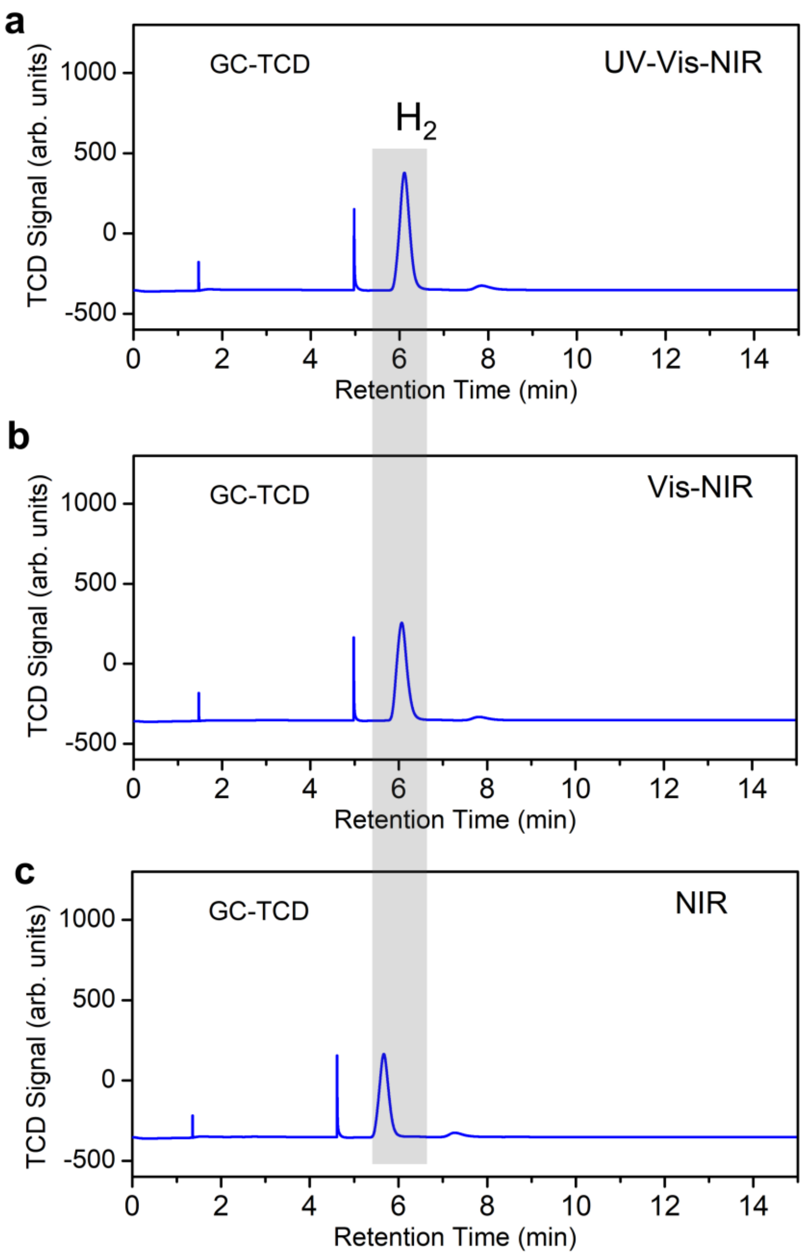

Supplementary Fig. 15 GC-TCD spectra of gas products obtained after 3 h reaction at 280 °C under different light wavelengths. (a) UV-Vis-NIR, (b) Vis-NIR, and (c) NIR irradiation.

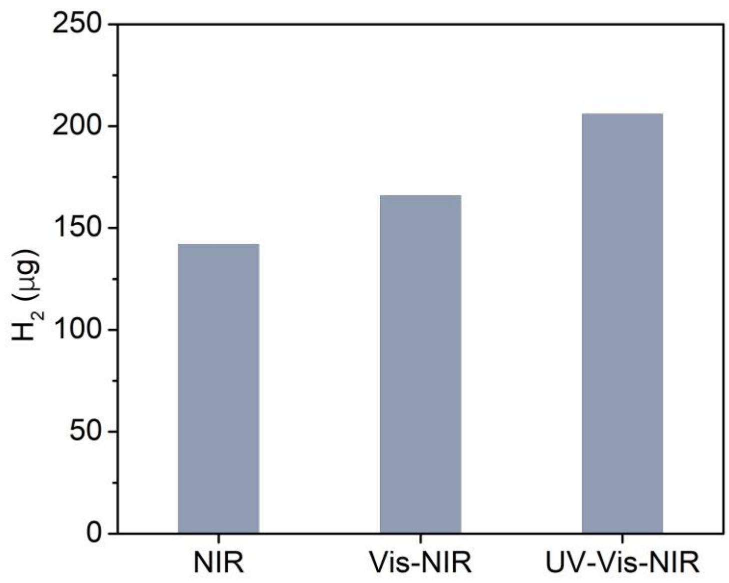

Supplementary Fig. 16 H_2 generation during wax refining under UV-Vis-NIR, Vis-NIR and NIR irradiation at 280 °C after 3 h reaction.

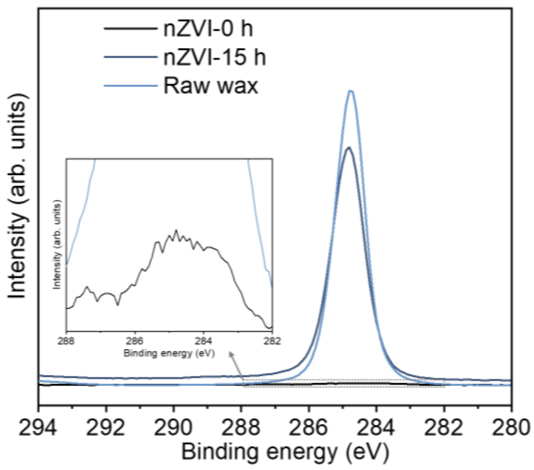

Supplementary Fig. 17 C *1s* XPS spectra of nZVI after 0 and 15 h reaction and raw wax.

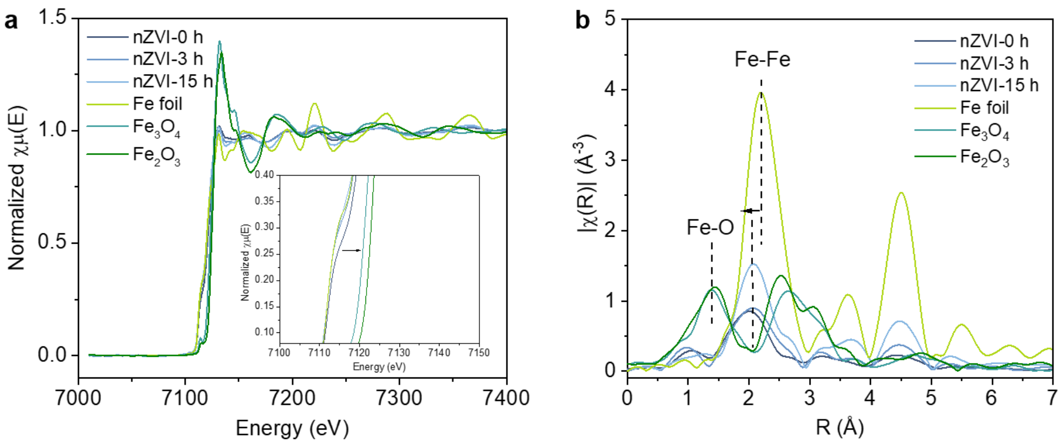

Supplementary Fig. 18 XAS measurements for nZVI catalysts. (a) Fe K-edge XANES, and (b) EXAFS spectra for as-prepared (nZVI-0 h) and spent catalyst after reaction 3 (nZVI-3 h) and 15 h (nZVI-15 h) reaction. Within 15 h reaction, the bulk Fe(0) composition of nZVI was maintained, as evidenced by the close X-ray absorption near edge structure (XANES) spectra for nZVI-0 h, 3 h, 5 h, with only a slight shift of the white line to lower enegies as the reaction proceeded. This result agreed well with our XPS result that the thin iron oxide shell within nZVI was partially reduced from Fe(III) to Fe(II). Consistently, the XAFS spectra of nZVI-0 h, 3 h, 5 h showed main peak at 2.1 Å assignable to low crystallinity-shortened Fe-Fe bond. As the reaction proceeded, the intensity of this peak increased, signifying the increased crystallinity of nZVI under photothermal heating.

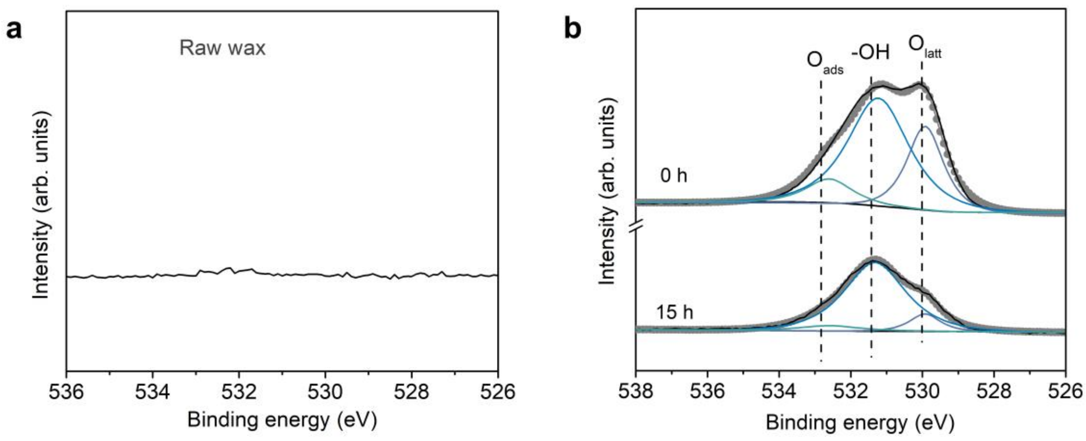

Supplementary Fig. 19 O *1s* XPS spectra. (a) raw wax, and (b) as-prepared nZVI and spent nZVI after 15 h reaction.

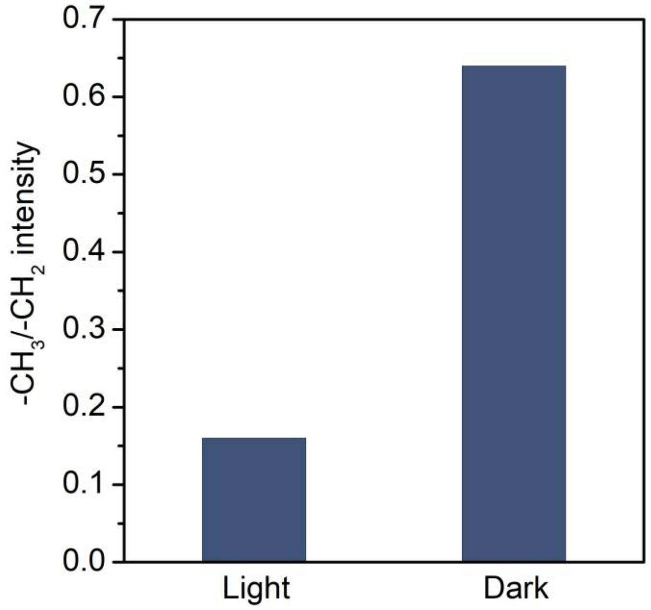

Supplementary Fig. 20 Comparison of $-CH_3/-CH_2$ intensity ratio from in-situ DRIFTS spectra of wax refining driven under light and dark.

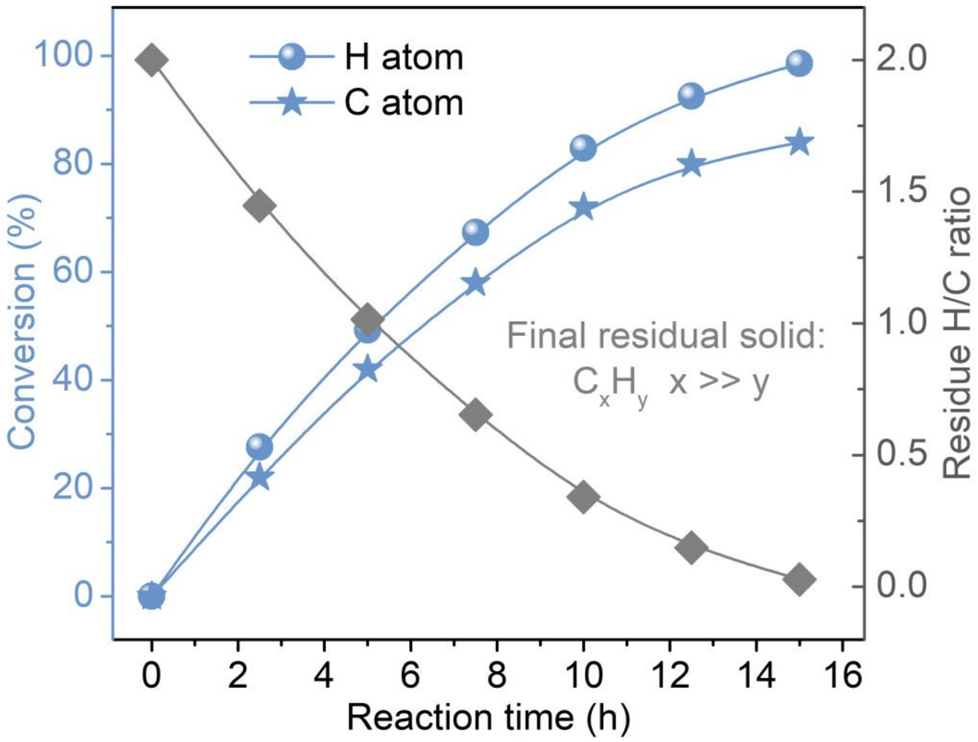

Supplementary Fig. 21 Time profile of C and H conversions of raw wax and the H/C ratio of the residue.

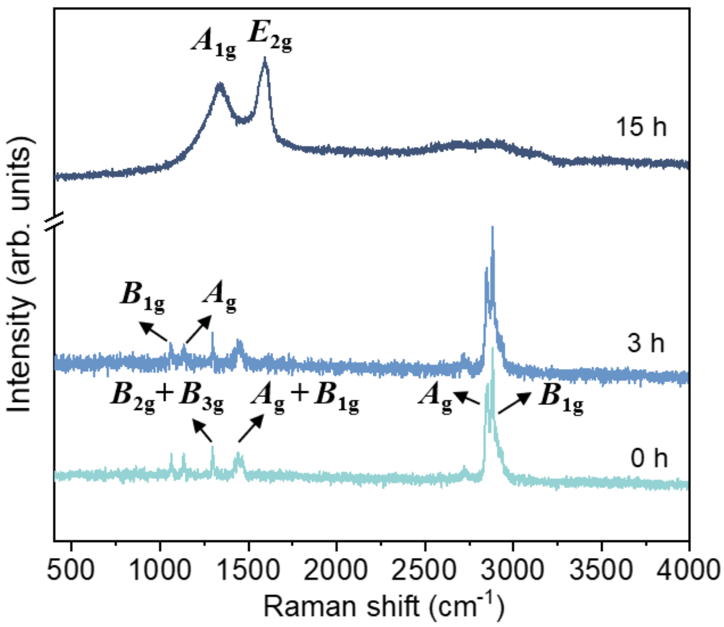

Supplementary Fig. 22 Raman spectra of PE wax-nZVI mixture after 0, 3, and 15 h reaction. PE wax's B_{1g} (1065 cm^{-1}) and A_g (1133 cm^{-1}) C-C stretching modes, B_{2g}+B_{3g} (1298 cm^{-1}) and A_g+B_{1g} (1436 cm^{-1}) -CH_2 twisting modes, and A_g (2849 cm^{-1}) and B_{1g} (2882 cm^{-1}) -CH_2 stretching modes were identified for the PE wax-nZVI mixture after 0 and 3 h reactions. Notably, only solid carbon's A_{1g} (1335 cm^{-1}) breathing and E_{2g} (1595 cm^{-1}) phonon modes were observed after 15 h reaction.

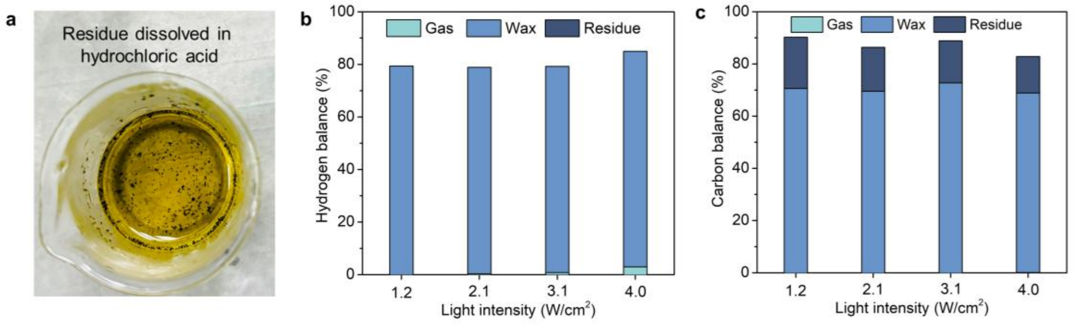

Supplementary Fig. 23 Coke isolation from the spent catalyst and the atom balance of solar wax refining. (a) Photo of the spent catalyst dissolved in the hydrochloric acid solution. The black colored precipitates are coke and the solid nZVI catalyst was dissolved demonstrating a typical yellow color of the Fe^{3+} solution. (b) Hydrogen, and (c) carbon balance during wax refining under light intensities of 1.2, 2.1, 3.1, 4.0 W/cm^2 after 15 h reaction.

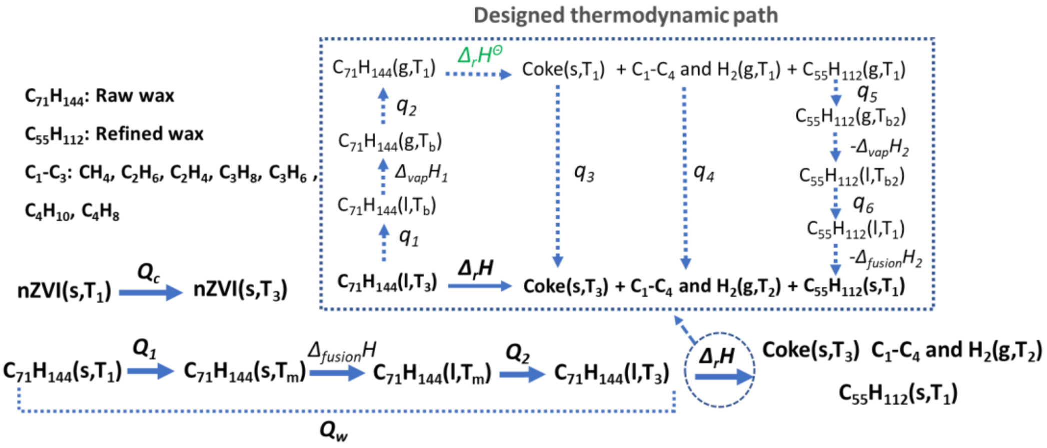

Supplementary Fig. 24 Thermodynamics for the energy efficiency calculation. Δ_rH represents the enthalpy change, calculated by the thermodynamic path indicated in the botted box, while Q$_c$, Q$_i$, and q$_i$ (i = 1-6) represent the absorbed or released heat.

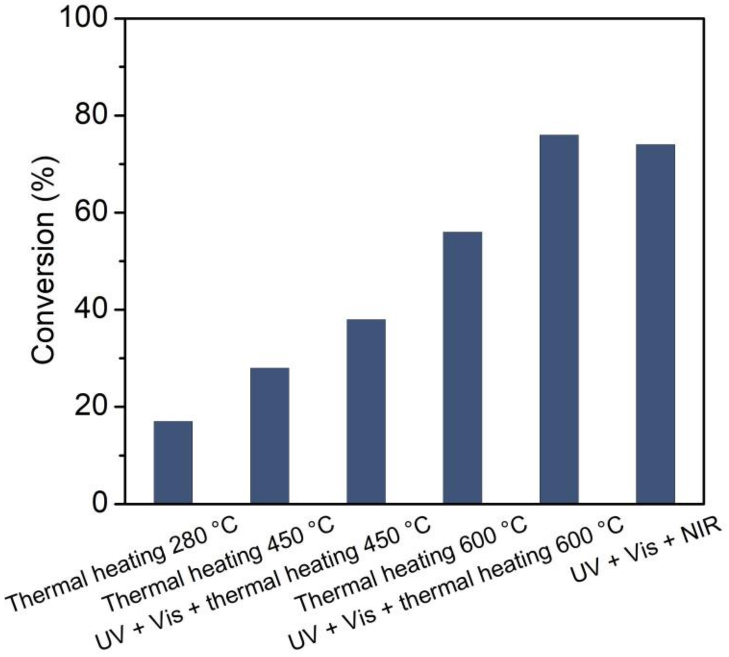

Supplementary Fig. 25 Wax conversion of thermochemical controls. Reaction condition: 100 mg nZVI, 100 mg raw wax, 1 bar Ar, 3 h thermal heating.

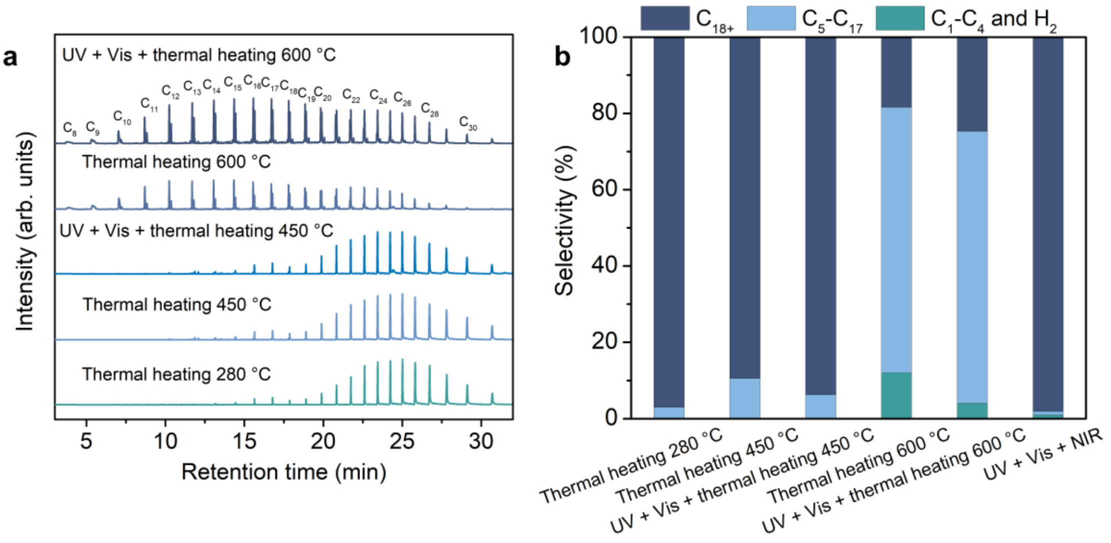

Supplementary Fig. 26 Products distribution in thermochemical control experiment. (a) GC-MS spectra of the liquid and wax product dissolved in n-hexane. (b) Products selectivity in dark at 280 °C, dark at 450 °C, 450 °C with UV-Vis light irradiation, dark at 600 °C, 600 °C with UV-Vis light irradiation, and full-spectrum light irradiation. Reaction condition: 100 mg nZVI, 100 mg raw wax, 3 hours heating time, 1 bar Ar, illuminated by light of certain wavelengths if specified.

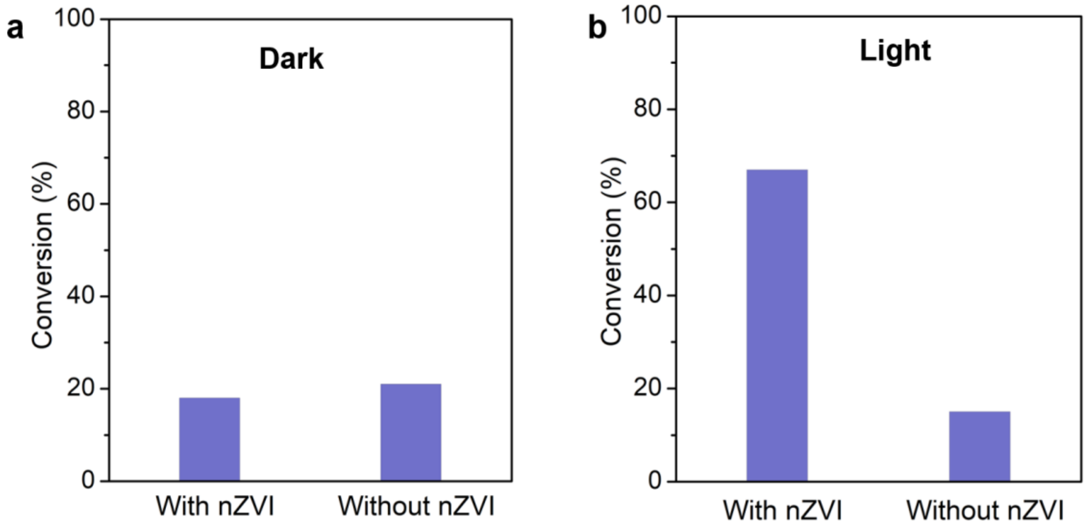

Supplementary Fig. 27 Comparison of wax refining under light and dark. Raw wax conversion ($m_{residue}/m_0$) in solar-driven photothermal (a) and dark heating (b) with and without nZVI. Reaction condition: 1 bar Ar, 100 mg raw wax, 100 mg nZVI, 3 h reaction, and 280 °C.

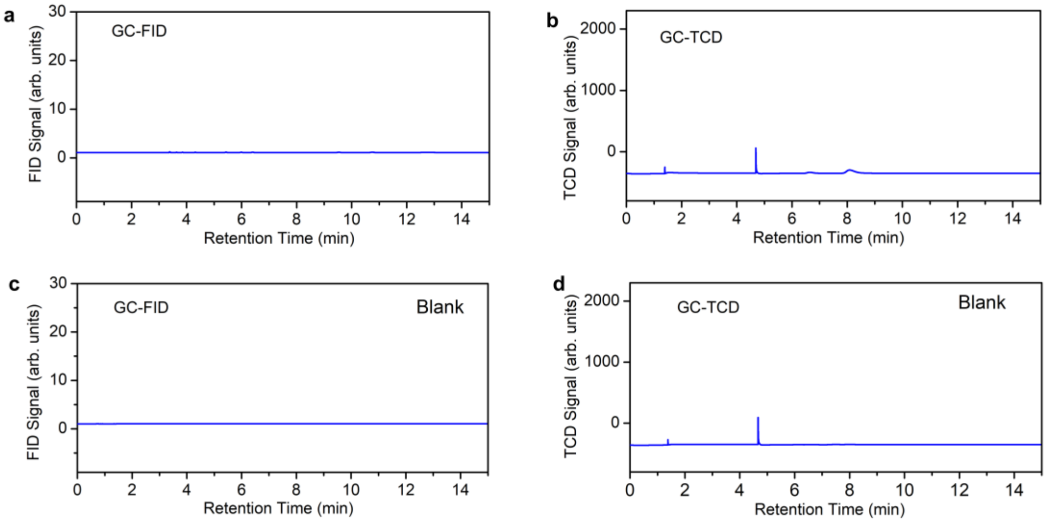

Supplementary Fig. 28 GC detection of gasous products of solar wax refining. (a) GC-FID, (b) GC-TCD spectra of gas products produced under dark heating reaction at 280 °C, and corresponding blank GC-FID (c) and GC-TCD (d) spectra. Notably, no gaseous hydrocarbon products were detected under dark (280 ± 5 °C), suggesting that the dark heating under this temperature is insufficient to activate raw wax for catalytic refining.

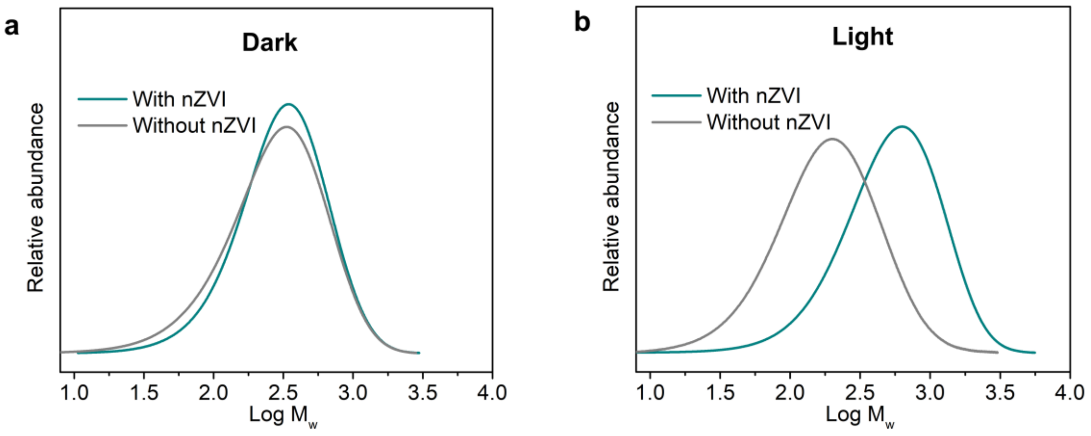

Supplementary Fig. 29 Comparison of molecular weight distributions of refined wax obtained under dark and like, with or without nZVI catalyst. (a) Molecular weight distribution of the product under dark heating reaction, according to HT-GPC measurements using 1,2-dichlorobenzene as the solvent. Similar low-carbon hydrocarbons were obtained, irrespective of the presence of the nZVI catalyst. (b) Molecular weight distribution of the product under light. Reaction condition: 1 bar Ar, 100 mg raw wax, 5 h reaction, and 280 °C.

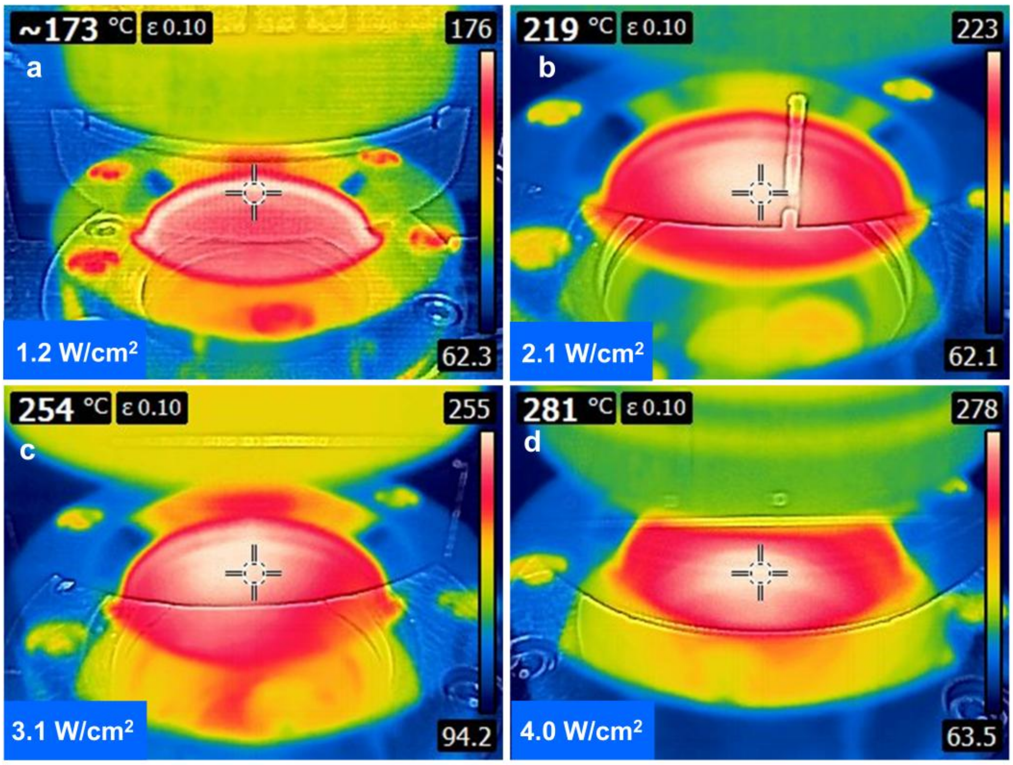

Supplementary Fig. 30 Digital photos of temperature mapping via an IR camera. Light intensities were 1.2 (a), 2.1 (b), 3.1 (c), 4.0 W/cm^2 (d), and the calibrated emissivity was 0.10 for the IR camera.

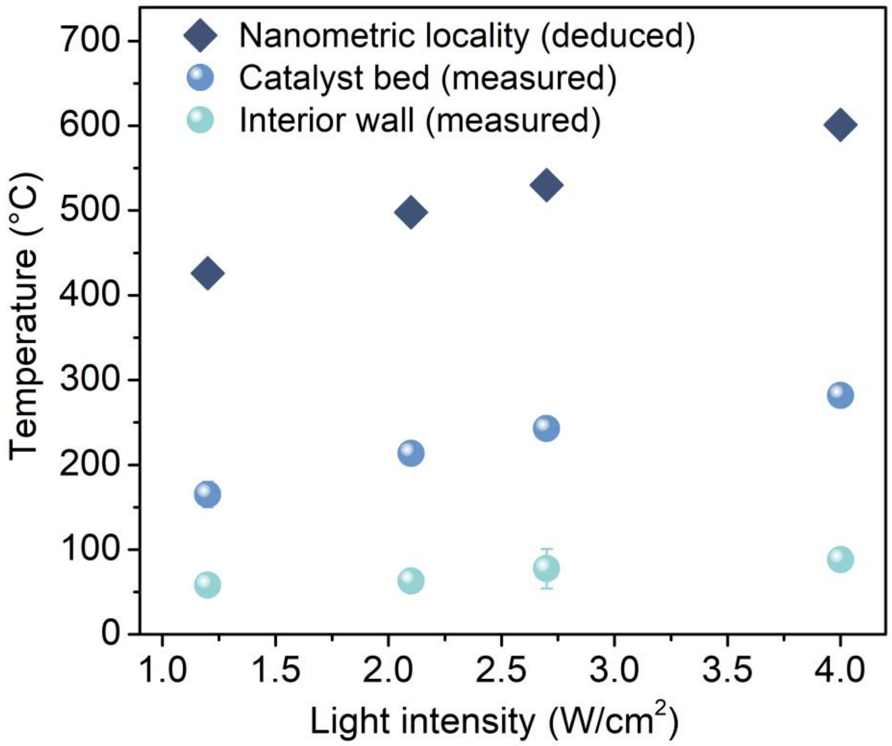

Supplementary Fig. 31 Temperatures of different positions inside the reactor under light. The temperature of interior walls inside the reactor and the temperature of the catalyst bed were measured through thermocouple under Xe lamp irradiation, and the local tempeature was deduced by the wax molecular length-boiling point relationship.

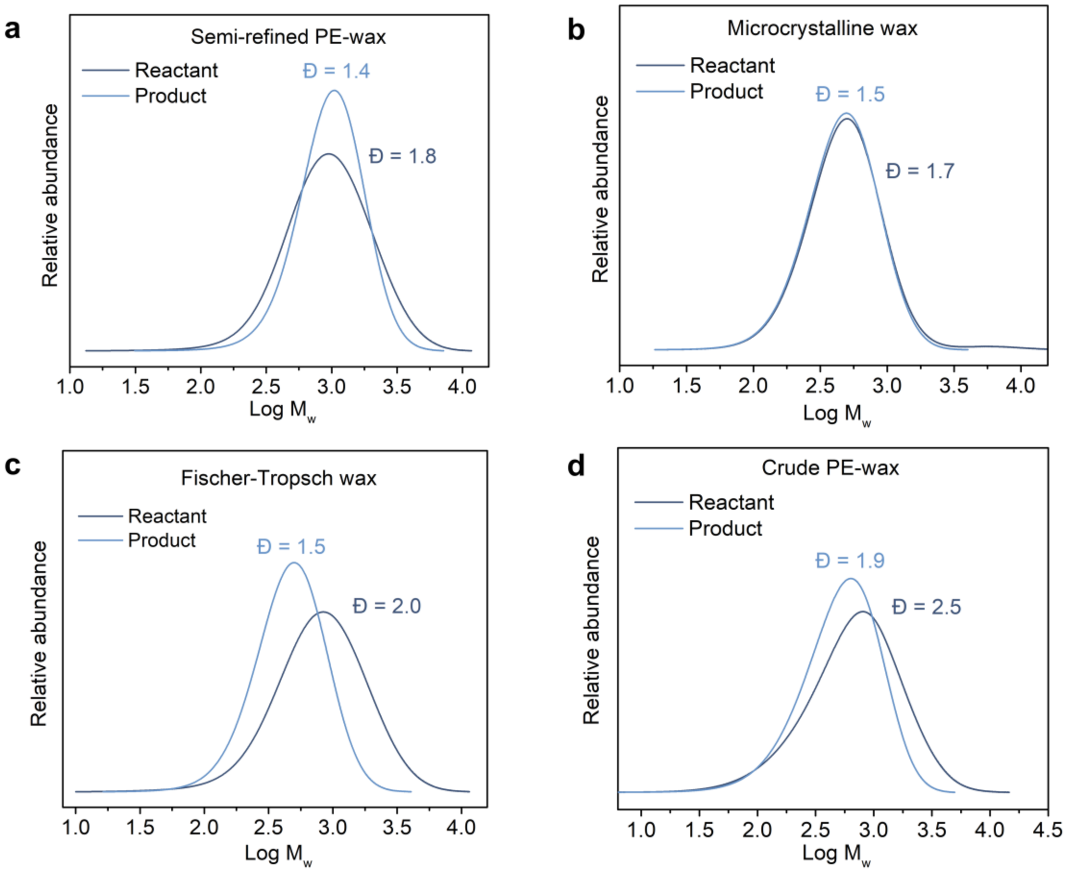

Supplementary Fig. 32 GPC measurements for raw and refined waxes of different types. Molecular weight distribution of semi-refined PE-wax (a), microcrystalline wax (b), Fischer-Tropsch wax (c), Crude PE-wax (d), and corresponding refined wax products, according to HT-GPC measurements using 1,2-dichlorobenzene as the solvent. Reaction condition: light intensity 4.0 W/cm^2, 1 bar Ar, 100 mg raw wax, 50 mg nZVI, and 5 h reaction.

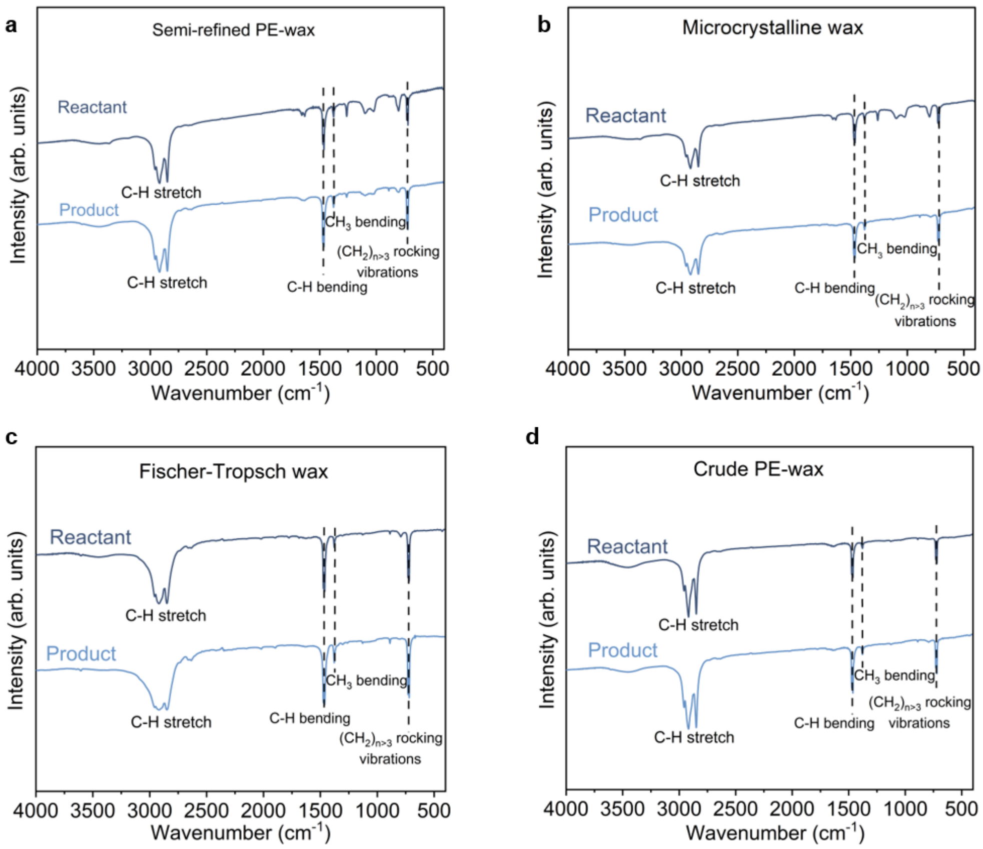

Supplementary Fig. 33 FT-IR characterization of raw and refined waxes. FT-IR spectra of (a) semi-refined PE-wax, (b) microcrystalline wax, (c) Fischer-Tropsch wax, (d) Crude PE-wax and corresponding wax products after refining. FT-IR analysis demonstrated that the refined waxes contained only linear saturated hydrocarbons, as evidenced by the characteristic bands at 2800-3000 cm^{-1} (C-H stretching), 1400-1500 cm^{-1} (C-H bending), and 700-750 cm^{-1} (CH_2 rocking). The complete absence of aromatic signatures (typically appearing above 3000 cm^{-1} and between 1650-1450 cm^{-1}) further confirmed the purely aliphatic nature and proved the quality of refined waxes.

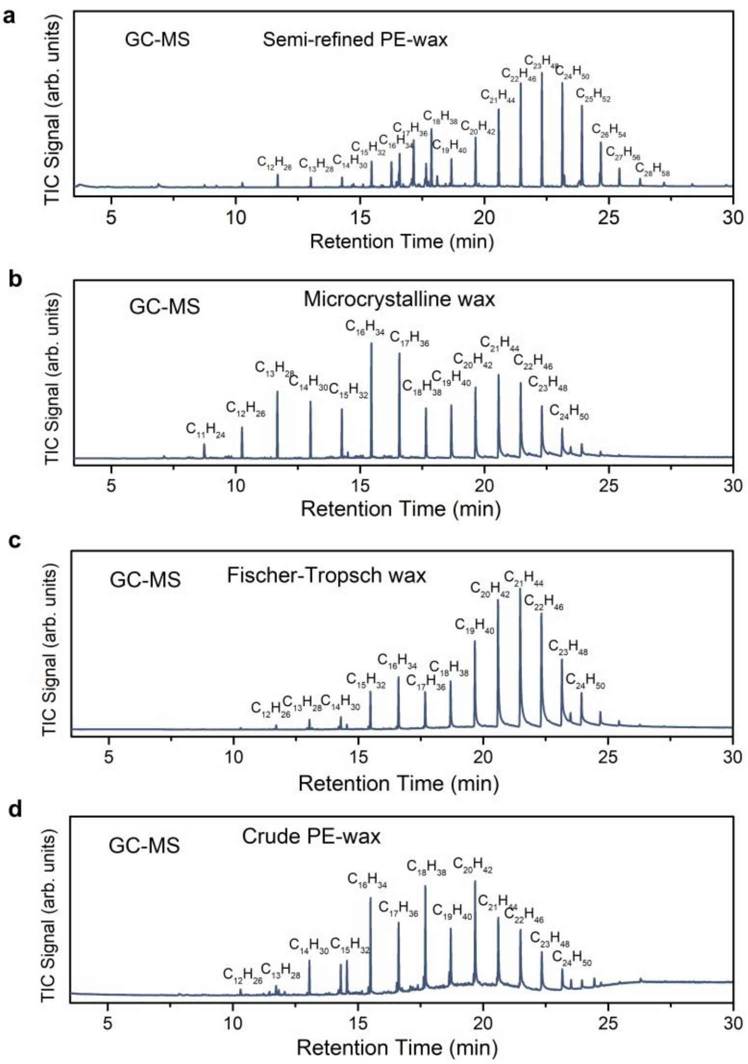

Supplementary Fig. 34 GC-MS characterization of refined waxes from different feedstocks. GC-MS characterization of wax products after the refining of (a) semi-refined PE-wax, (b) microcrystalline wax, (c) Fischer-Tropsch wax, and (d) Crude PE-wax. The GC-MS analysis of the refined waxes revealed prominent peaks corresponding to linear alkanes (C_{12}-C_{28}) without cyclized and aromatic compounds.

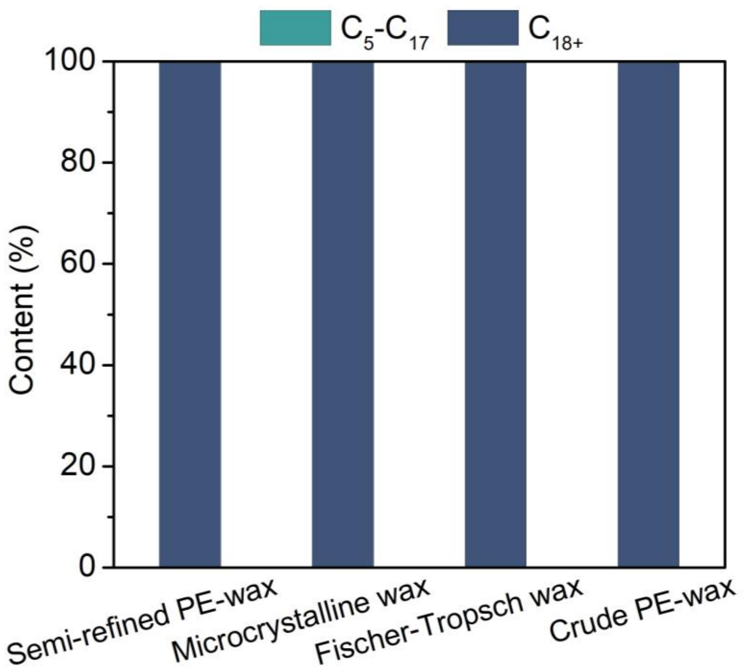

Supplementary Fig. 35 Non-wax (C_5-C_{17}) contents in semi-refined PE-wax, microcrystalline wax, Fischer-Tropsch wax and crude PE-wax. Quantified by GC-FID, non-wax components (C_5-C_{17}) in semi-refined PE-wax, microcrystalline wax, Fischer-Tropsch wax and crude PE-wax were only 0%, 0%, 0% and 0.08%, respectively.

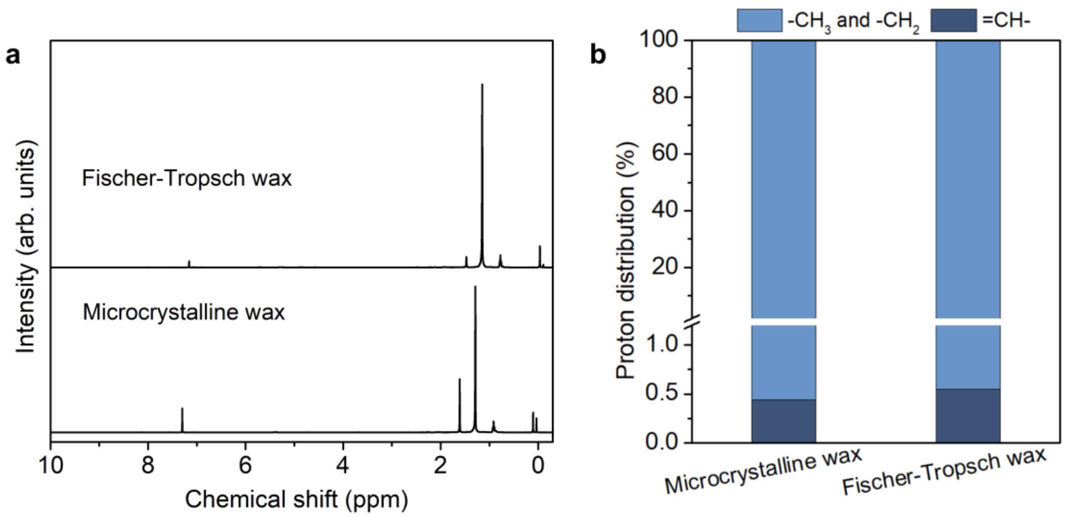

Supplementary Fig. 36 Quantifications for protons of different chemical environments in Fischer-Tropsch and microcrystalline waxes. (a) ^{1}H NMR spectra and (b) corresponding proton distribution in refining products obtained from Fischer-Tropsch wax and microcrystalline wax. Solvent: $CDCl_3$.

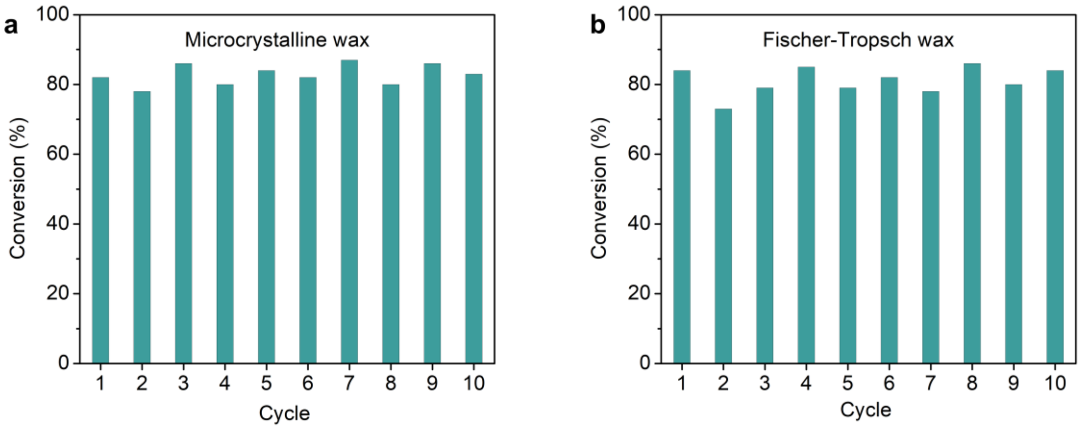

Supplementary Fig. 37 Stability tests. nZVI-enabled solar refining for Fischer-Tropsch wax (a) and microcrystalline wax (b).

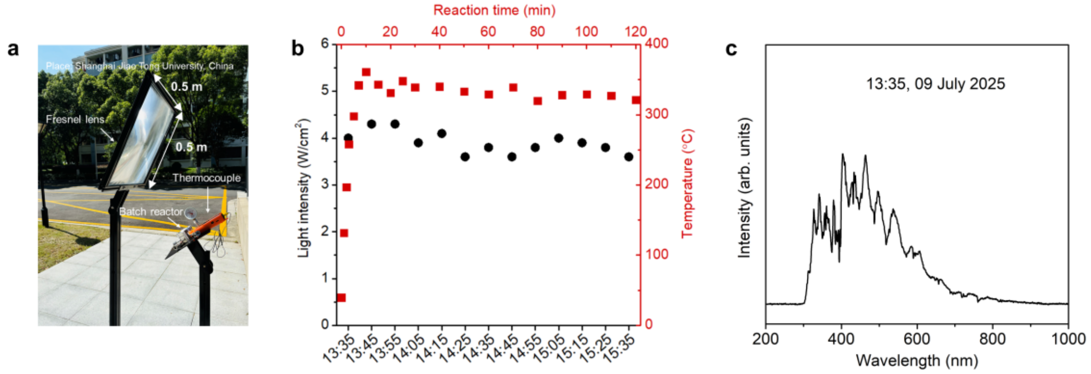

Supplementary Fig. 38 The upscaled outdoor solar wax refining driven by sole sunlight. (a) Photo of the outdoor reactor equipped with a Fresnel lens. (b) Temperature profiles of the mixture consisting 1 g nZVI and 5 g raw wax in the designed photo reactor under sunlight, and (c) corresponding solar spectrum on 9 July 2025, detected by a fiber optic spectrometer.

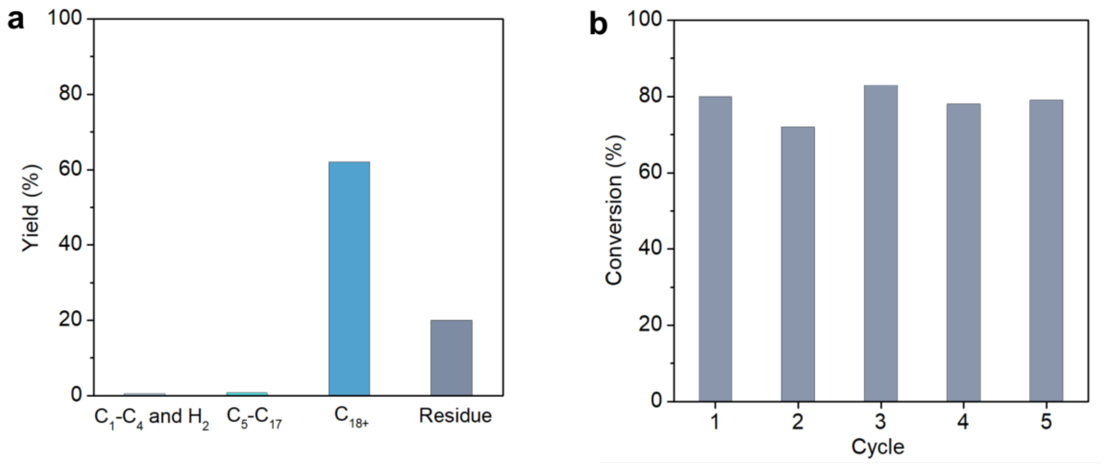

Supplementary Fig. 39 The performance upscaled outdoor solar wax refining driven by sole sunlight. (a) Mass yield of the gas products (C_1-C_4 and H_2), liquid component (C_5-C_{17}), wax component (C_{18+}) and solid residue after reaction 2 h. (b) Reusability of the nZVI catalyst for outdoor solar-driven wax refining.

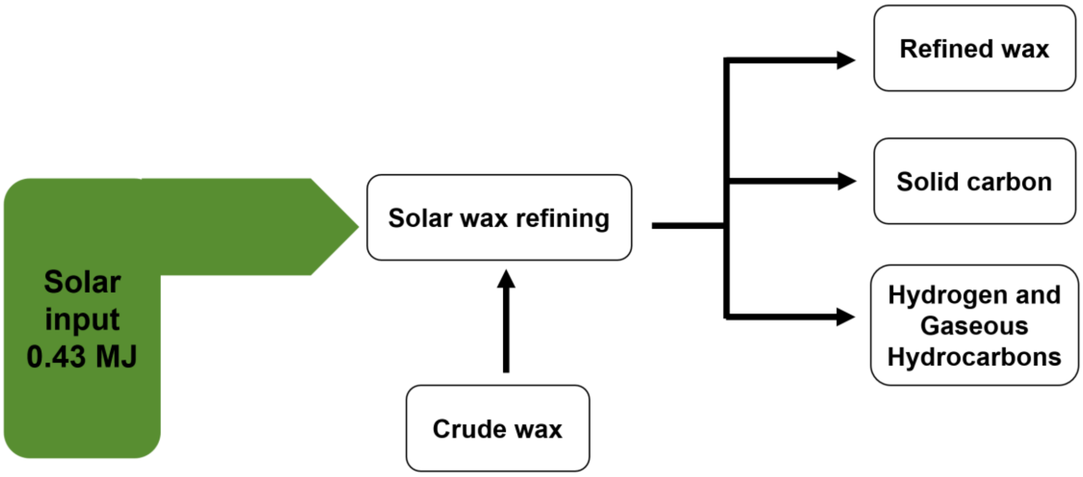

Supplementary Fig. 40. Process flowsheet for solar wax refining with 10 kt/year processing intake.

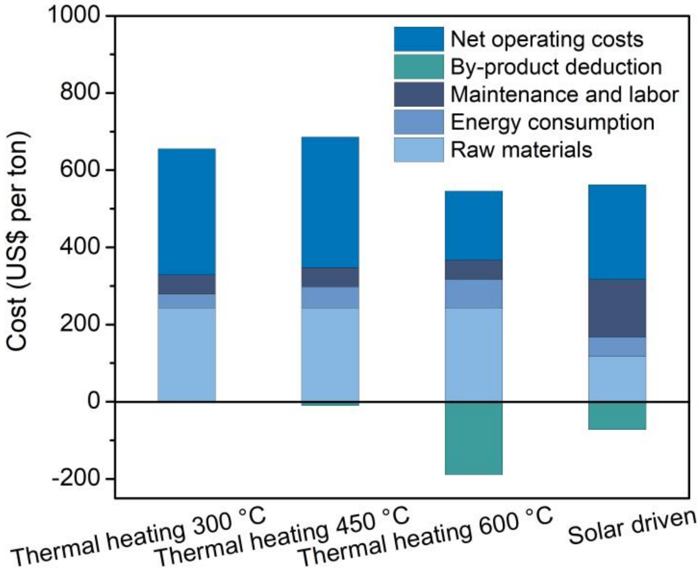

Supplementary Fig. 41 TEA of the solar-driven wax refining process. Costs of the thermal heating under 300, 450, and 600 °C and solar driven wax refining strategies. Product distributions were obtained from Supplementary Fig. 26.

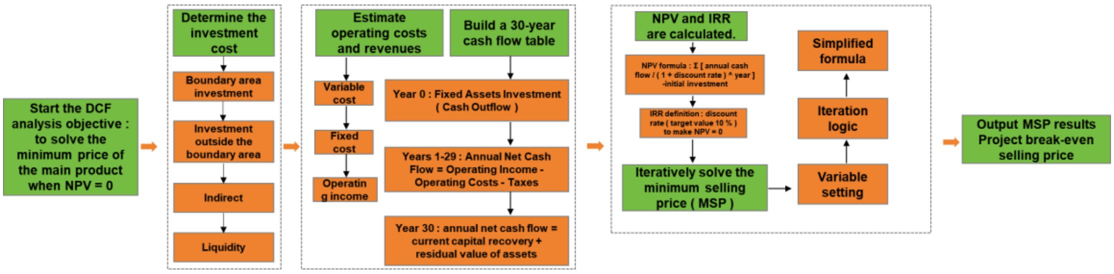

Supplementary Fig. 42 EES Finance. The core goal of financial feasibility analysis is to calculate the Minimum Selling Price (MSP) of main products using the Discounted Cash Flow (DCF) model. MSP refers to the break-even price that ensures the project's lifecycle Net Present Value (NPV) equals 0 while meeting the 10% target Internal Rate of Return (IRR). The analysis follows five key steps: first, define full-scope investment costs including equipment and supporting facilities; second, break down variable costs (such as feedstock and catalysts) and fixed costs (such as labor and maintenance) during the operation period, and integrate the revenue structure of main and by-products; third, compile a 30-year cash flow statement that distinguishes between construction, operation and end-of-project recovery phases; fourth, anchor the financial targets of "NPV=0, IRR=10%" by calculating NPV and IRR; finally, iteratively adjust the main product price to solve for the MSP that meets the targets. The supporting parameter table provides quantitative backing, which not only clarifies basic boundaries such as plant lifespan and operating rate, defines financial rules such as tax rate and discount rate, and details the composition of investment and operating costs—for example, feedstock costs account for 60%-80% of variable costs—but also differentiates the revenue roles of main and by-products. Taking specific parameters as examples, a 90% operating rate determines actual production capacity and unit costs, by-product revenue offsetting total costs can lower MSP, and the MSP sensitivity to feedstock prices

(approximately 12%-15%) directly identifies the core risk to project profitability. In summary, the flowchart serves as the logical framework of the analysis, while the parameter table acts as the quantitative foundation; together, they support the calculation of the minimum price threshold for chemical projects and provide a core tool for evaluating commercial viability.

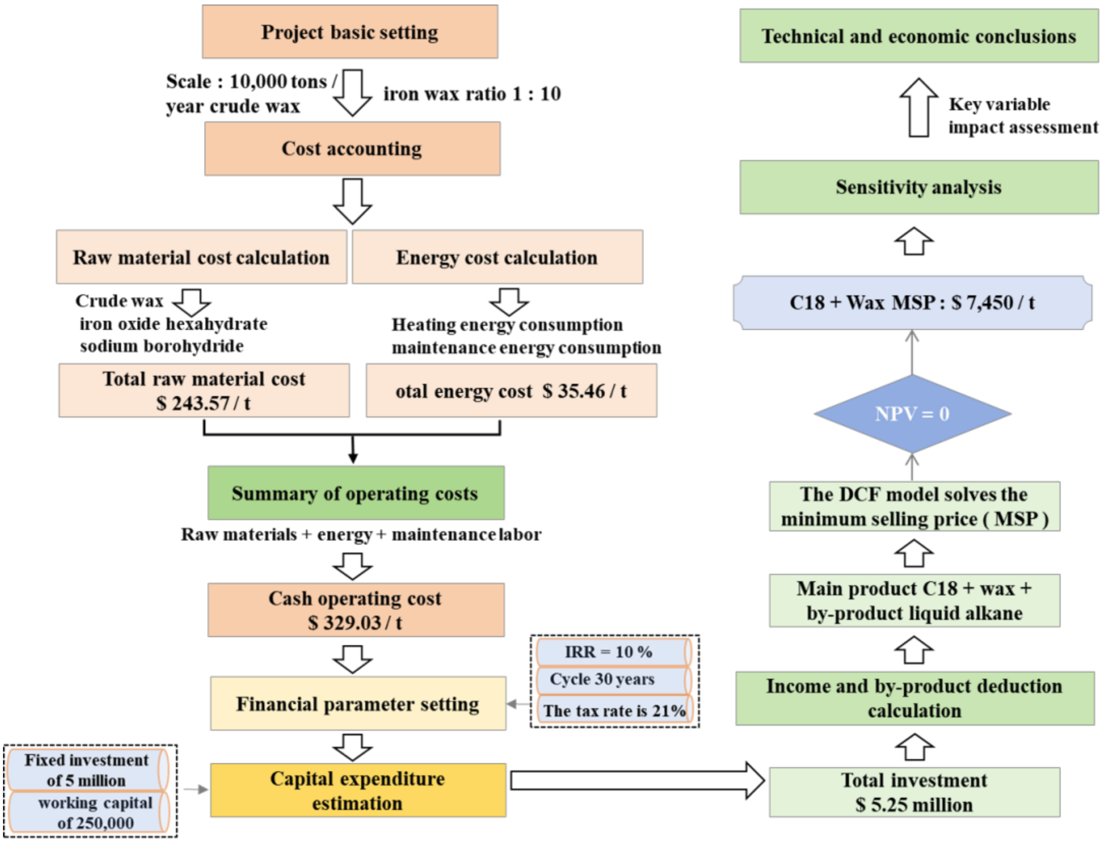

Supplementary Fig. 43 Core cost & investment parameters. This flowchart starts with project baseline setup such as defining scale and process parameters, and then the calculation of raw material/energy costs. Combined with financial assumptions (e.g., 10% IRR) and total investment (USD 5.25 million), the DCF model derives the minimum selling price (MSP) of the main product (C_{18+} wax) at USD 7,450/ton. Finally, sensitivity analysis evaluates how key variables affect economic performance. The tables distill core data (costs, investment, pricing) with remarks, while comparing the economics of different process pathways-the 300 °C low-temperature pathway is the least economical due to low product yield.

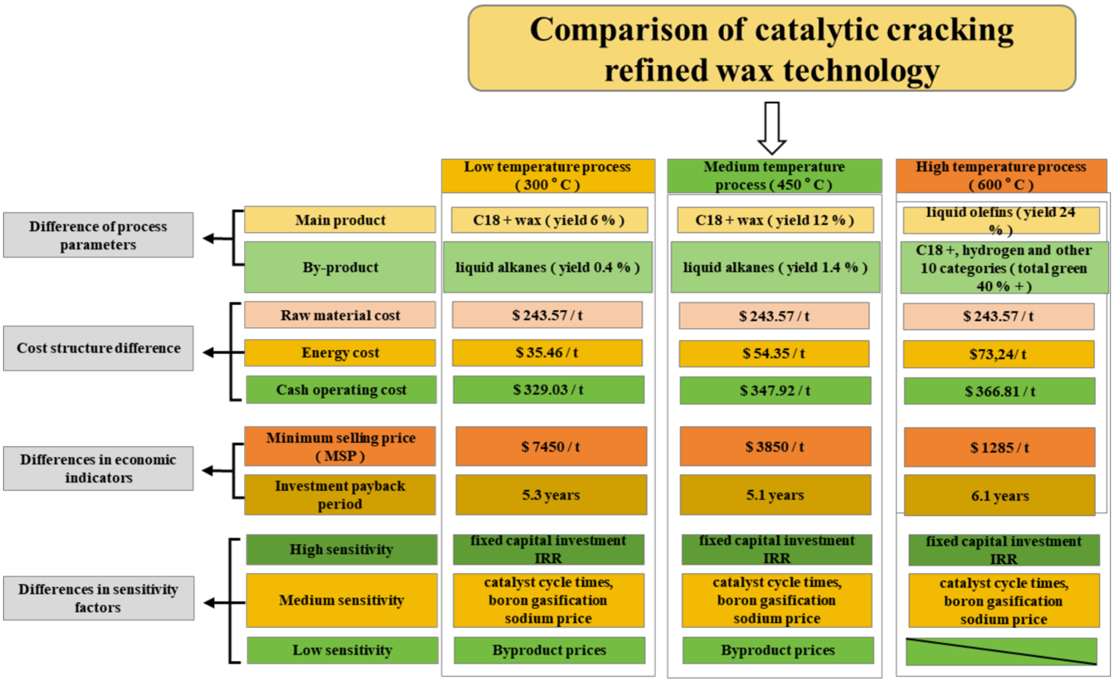

Supplementary Fig. 44 Comparison of catalytic cracking refined wax technology. The flowchart systematically compares three catalytic cracking refined wax technologies (low-temperature: 300 °C, medium-temperature: 450 °C, high-temperature: 600 °C) around four core dimensions: process parameter differences, cost structure differences, economic indicator differences, and sensitivity factor differences. Two supporting consolidated tables integrate key information with concise “Key Difference Explanations” to facilitate quick grasp of core gaps.

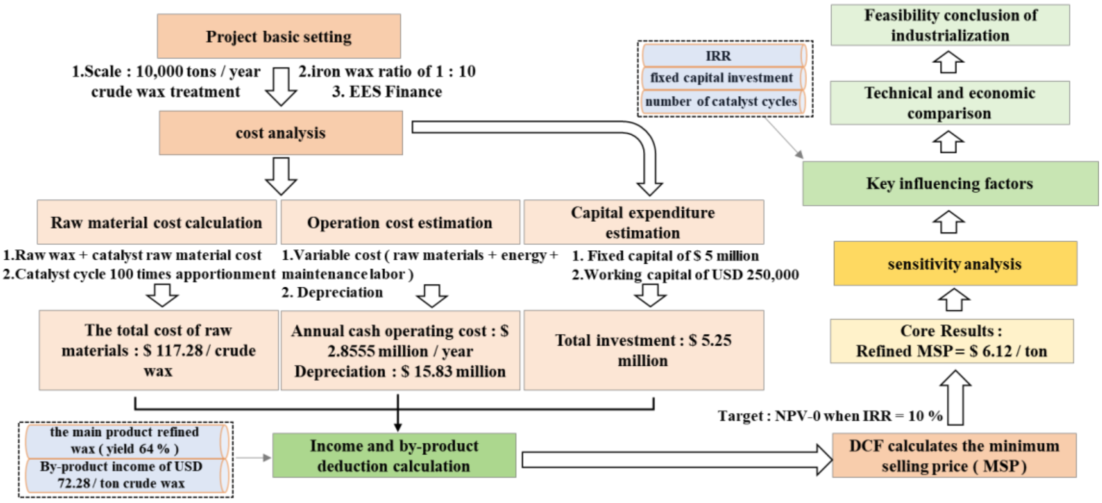

Supplementary Fig. 45 Technoeconomic analysis of solar wax refining. The flow chart starts with core premises such as processing scale, iron-wax ratio, financial assumptions, followed by "cost analysis → capital expenditure → revenue calculation → pricing calculation → sensitivity analysis → economic comparison" based on the Discounted Cash Flow (DCF) analysis. Finally, the industrialization feasibility is evaluated.

Supplementary Tables

Supplementary Table 1. Market value of different waxes.

Types	Prices ($/ton) †
Raw wax¶	600
Waste polyethylene wax*	850
Virgin LDPE pyrolysis waxes*	1700-1550
Synthetic LDPE wax*	2800
Synthetic HDPE wax*	5400
High-end grade wax¶	6300

† International or China domestic prices (1 Yuan = 0.14 USD) obtained from ECHEMI, retrieved Oct. 2025.

*Pricing information for waxes as per quotation from Nanjing Tianshi Wax Co., Ltd.

¶Wax pricing based on Sinopec internal quotation.

Supplementary Table 2. Comparison of this work with reported wax refining systems.

Catalyst	Reaction condition	Products and performance	Ref.
Fe@Fe_2O_{3-x}	Xe lamp illumination 3 h; Ar atmosphere	Conversion > 70%; Refined wax selectivity 99%	***This work***
Pd/Hβ	380 °C; WHSV 1.77 h^{-1}, H_2 180 mL/min	64.8% conversion; 48.5% naphtha-ranged liquid yield	*J. Anal. Appl. Pyrol.* **161**,105424 (2022)
Zeolite Y	450 °C; N_2 atmosphere	60% liquid yield	*Energy Fuels* **35**, 9450-9461 (2021)
HZSM-5	FCC conditions; 500-550 °C	About 50% gasoline yield; About 70% conversion	*Chem. Eng. J.* **132**, 17-26 (2007)
FCC Equilibrium Catalyst	FCC conditions; 500-560 °C	Conversion 41.4-62.9 wt %; 23.8-31.1 wt % naphtha and 13.7-18.1wt % LPG fractions	*Energy Fuels* **33**, 5191-9199 (2019)
Pt/Al_2O_3	6-8 h; 350 °C; H_2 180 bar	Saturated hydrocarbon	*Energy Fuels* **37**, 16181-16185 (2023)

Supplementary Table 3. The integrated ^1H NMR peak area for protons in wax products obtained under different light intensities (1.2, 2.1, 3.1 and 4.0 W/cm^2).

Group	-CH_3	-CH_2-	-CH_2-CH=	-CH=CH-	-CH=CH_2	-CH=CH_2
**1.2 W/cm^2**	24925.8	203542	1269.5	428.2	0	0
**2.1 W/cm^2**	2459.7	20209.7	231.6	80.1	0	0
**3.1 W/cm^2**	23832.5	195781	1386.7	585.5	138.9	251.8
**4.0 W/cm^2**	349057.6	2277334	27957.9	6874.1	2483.3	6374.3

Supplementary Table 4. The integrated ^{1}H NMR peak areas of protons in wax products obtained under different light wavelengths (NIR, Vis-NIR, UV-Vis-NIR).

**Group**	**-CH_3**	**-CH_2-**	**-CH_2-**CH=	**-CH=CH-**	**-CH**=CH_2	-CH=**CH_2**
**NIR**	35260.3	225825.2	1347.5	568.5	0	0
**Vis-NIR**	27767.8	174508.6	1458.6	864.0	0	0
**UV-Vis-NIR**	30559.9	189240.9	3118.1	1585.5	0	0

Supplementary Table 5. Atomic ratio of Fe, C and O in the nZVI catalyst based on XPS results.

Sample	Fe atomic ratio	O atomic ratio	C atomic ratio
**nZVI-0 h**	19.6%	52.0%	28.4%
**nZVI-15 h**	1.2%	9.9%	89.9%

Supplementary Table 6. XPS fitting results for the Fe 2p spectrum.

Sample	Fe^{0}/Fe_{total}	$Fe(II)/Fe_{total}$	$Fe(III)/Fe_{total}$
**nZVI-0 h**	10.4%	46.5%	43.1%
**nZVI-15 h**	0	63.9%	36.1%

Supplementary Table 7. Thermodynamic parameters, $\Delta_f H$ and $S^{\theta}_{gas,\ est}$, for sub-groups based on the Benson group increments method.

**Group**	**$-CH_3$**	**$-CH_2-$**	**$-CH=$**	**$=CH_2$**
**$\Delta_f H^{\theta}_{gas,\ est}$** kJ/mol	-42.7	-21.0	36.0	26.0
**$S^{\theta}_{gas,\ est}$** J/(mol·K)	127.2	39.4	33.3	115.5

Supplementary Table 8. Thermodynamic parameter $C_{p\text{-}g}$ [J/(mol·K)] for sub-groups based on the Benson group increments method.

**Group** / **T(K)**	**-CH_3**	**-CH_2-**	**-CH=**	**=CH_2**
**300**	25.9	23.0	17.4	21.3
**400**	32.8	29.1	21.0	26.6
**500**	39.3	34.5	24.3	31.4
**600**	45.2	39.1	27.2	35.6
**800**	54.5	46.3	32.0	42.1
**1000**	61.8	51.6	35.4	47.2
**1500**	73.6	59.4	40.3	55.2

Supplementary Table 9. Thermodynamic parameter $C_{p\text{-}g}/T$ [J/(mol·K^2)] for sub-groups based on the Benson group increments method.

Group / T(K)	$-CH_3$	$-CH_2-$	$-CH=$	$=CH_2$
**300**	0.077	0.086	0.058	0.071
**400**	0.073	0.082	0.053	0.067
**500**	0.069	0.079	0.049	0.063
**600**	0.065	0.075	0.045	0.059
**800**	0.058	0.068	0.040	0.053
**1000**	0.052	0.062	0.035	0.047

Supplementary Table 10. Quantitative statistics of chemical group.

	Chemical formula	$-CH_3$ (mmol/g)	$-CH_2-$ (mmol/g)	$-CH=$ (mmol/g)	$=CH_2$ (mmol/g)
**Raw wax**	$n\text{-}C_{71}H_{144}$	2.0	69	0	0
**Refined wax**	$n\text{-}C_{55}H_{112}$	2.6	69	0	0

Supplementary Table 11. Entropy change ($\Delta_{l\text{-}g}S$) and phase transition enthalpy ($\Delta_{\mathrm{vap}}H$, $\Delta_{\mathrm{vap}}H_1$, $\Delta_{\mathrm{vap}}H_2$) for the thermodynamic path.

	S_l (J/K)	S_g (J/K)	$\Delta S_{l\text{-}g}$ (J/K)	Heat of phase transition
Raw wax (at T_b, 939 K)	0.13	0.60	0.47	$\Delta_{\mathrm{vap}}H_1$ = 440.3 J
Refined wax (at T_b, 873 K)	0.09	0.44	0.39	$\Delta_{\mathrm{vap}}H_2$ = 305.3 J

Supplementary Table 12. The heat absorbed by the raw wax (Q_w) during the wax melting process, with the temperature increased from T_1 to T_3.

**Solid, $T_1 \rightarrow T_m$**	**$\Delta_{fusion}H$**	**Liquid, $T_m \rightarrow T_3$**	**Q_w**
8.8 J	41.3 J	147.5 J	197.7 J

Supplementary Table 13. The heat absorbed (+) or released (-) by raw wax and refined wax in designed thermodynamic path ($q_{1\text{-}6}$).

**q_1**	**q_2**	**q_3**	**q_4**	**q_5**	**q_6**
24.0 J	-179.7 J	14.6 J	2.0 J	118.8 J	-147.3 J

Supplementary Table 14. The standard reaction enthalpy ($\Delta_r H^{\Theta}$) calculated by $\Delta_f H$, the actual reaction enthalpy ($\Delta_r H$) calculated by S11, and the fusion enthalpy ($\Delta_{\mathrm{fusion}} H_2$) for refined wax in the designed thermodynamic path.

$\Delta_r H^{\Theta}$	$\Delta_{\mathrm{fusion}} H_2$	$\Delta_r H$
43.0 J	29.1 J	-21.0 J

Supplementary Table 15. The heat absorbed by the catalyst (Q_c), the enthalpy changes of the chemical reaction ($\Delta_r H$), the energy required for the overall reaction (E_{re}), and the energy efficiency (η).

Q_c	Q_w	$\Delta_r H$	E_{re}	E_{input}	η
34.1 J	197.7 J	-21.0 J	210.7 J	203472 J	0.1%

Supplementary Table 16. Molecular weights of wax products originating from different reactants.

Reactant	Wax products		
	M_w	M_n	$Đ$
Semi-refined PE-wax	1143	803	1.4
Fischer-Tropsch wax	557	368	1.5
Microcrystalline wax	549	372	1.5
Crude PE-wax	672	359	1.9

Supplementary Table 17. Molecular weight for different wax types.

	M_w (g/mol)	M_n (g/mol)	$Đ$
Semi-refined PE-wax	1236	687	1.8
Fischer-Tropsch wax	1085	543	2.0
Microcrystalline wax	638	384	1.7
Crude PE-wax	988	393	2.5

Supplementary Table 18. Products, Price & Sensitivity Analysis.

Category	Item	Value/parameter	Unit	Remarks
**Raw Material Cost**	Crude Wax	85.71	USD/ton	Direct raw material cost
	$FeCl_3 \cdot 6H_2O$	3.60	USD/ton	100kg catalyst consumption ÷ 100 cycles
	$NaBH_4$	154.26	USD/ton	100kg catalyst consumption ÷ 100 cycles
	Total Raw Material	243.57	USD/ton	Sum of crude wax, $FeCl_3 \cdot 6H_2O$, and $NaBH_4$ costs
**Energy Cost**	Heating Energy	25.46	USD/ton	25 °C→300 °C, thermal efficiency 60%
	Maintenance Energy	10.00	USD/ton	Heat loss at 300°C for 2 hours
	Total Energy	35.46	USD/ton	Sum of heating and maintenance energy costs
**Operating Cost**	Cash Operating Cost	329.03	USD/ton	Raw materials + energy + maintenance labor
**Investment**	Fixed Investment	5,000,000	USD	Estimated for continuous plant (EES-based)
	Working Capital	250,000	USD	5% of fixed investment
	Total Investment	5,250,000	USD	Fixed investment + working capital
**Financials**	IRR	10%	-	Target return rate for MSP calculation
	Tax Rate	21%	-	U.S. federal income tax rate
	Project Lifecycle	30	Years	Straight-line depreciation, 5% residual value
**Products & Price**	C_{18+} Wax MSP	7,450	USD/ton	Minimum selling price to achieve NPV=0
	Liquid Alkanes (By-product)	2.80	USD/ton	Naphtha spot price, by-product revenue
**Sensitivity Analysis**	Fixed Investment (+20%)	8,050	USD/ton	High impact on MSP
	Fixed Investment (-20%)	6,850	USD/ton	
	Catalyst Cycles (80x)	7,650	USD/ton	Medium impact on MSP
	Catalyst Cycles (120x)	7,300	USD/ton	
**Process Comparison**	Solar Pathway (Refined Wax)	612	USD/ton	Most economical pathway
	300°C Low-Temp Pathway	7,450	USD/ton	Least economical due to low C_{18+} wax yield (6%)

Supplementary Table 19. Comparison of process parameters and cost structure.

Dimension	Low-temperature Process (300 °C)	Medium-temperature Process (450 °C)	High-temperature Process (600 °C)	Key difference explanation
**Core Process Parameters**				Higher temp boosts reaction efficiency, increasing product variety/yield but raising energy use
**- Main Product**	C_{18+} Wax	C_{18+} Wax	Liquid Olefins	Low/medium temp makes long-chain waxes; high temp cracks waxes into high-value olefins
**- Main Product Yield**	60 kg/ton Crude Wax (6%)	120 kg/ton Crude Wax (12%)	240 kg/ton Crude Wax (24%)	Yield rises with temp—high-temp yield is 4x that of low-temp
**- By-Product Types**	1 Type (Liquid Alkanes)	1 Type (Liquid Alkanes)	10 Types (C_{18+} Wax, Hydrogen, Methane, etc.)	High-temp cracks more thoroughly; low/medium temp only makes small short-chain alkanes
**- Total By-Product Yield**	4 kg/ton Crude Wax (0.4%)	14 kg/ton Crude Wax (1.4%)	400 kg/ton Crude Wax (over 40%)	High-temp by-product yield is 28-100x that of low/medium temp
**- Actual Heating Energy Consumption**	254.63 kWh/ton	393.52 kWh/ton	532.4 kWh/ton	Energy use correlates with temp—high-temp use is 2.1x that of low-temp
**- Energy for Temp Maintenance**	100 kWh/ton	150 kWh/ton	200 kWh/ton	Higher temp = more heat loss = higher maintenance energy
**Cost Structure (USD/ton Crude Wax)**				Raw material costs are same; energy costs rise with temp—high-temp uses by-product income to balance
**- Crude Wax Cost**	85.71	85.71	85.71	Uniform user-specified price, no difference
**- Catalyst Cost**	157.86 (Ferric Chloride 3.60 + Sodium Borohydride 154.26)	157.86 (Ferric Chloride 3.60 + Sodium Borohydride 154.26)	157.86 (Ferric Chloride 3.60 + Sodium Borohydride 154.26)	Same 1:10 iron-chloride ratio & 100 cycles—dosage-price consistent
**- Total Raw Material Cost**	243.57	243.57	243.57	No raw material difference; gaps in energy/income
**- Energy Cost**	35.46 (Heating 25.46 +	54.35 (Heating 39.35 +	73.24 (Heating 53.24 +	High-temp energy cost is \$37.78/ton higher than low-temp (20% of cash op cost)

	Maintenance 10.00)	Maintenance 15.00)	Maintenance 20.00)	
**- Maintenance & Labor Cost**	50.00	50.00	50.00	Uniform industry estimate, no difference
**- Total Cash Operating Cost**	329.03	347.92	366.81	High-temp has highest op cost, covered by high-value products
**- Annual Cash Operating Cost**	2,961,270 (9,000 tons/year)	3,131,280 (9,000 tons/year)	3,301,290 (9,000 tons/year)	High-temp annual cost is $339,900 higher than low-temp

In terms of process parameters, low/medium-temperature processes mainly produce C_{18+} wax with low by-product variety and yield, while the high-temperature process generates liquid olefins as the main product along with 10 types of by-products, showing significant advantages in product diversity and total yield. Regarding costs, raw material costs are consistent across the three processes (all 243.57 USD/ton crude wax), while energy costs increase with temperature (35.46 USD/ton for low-temperature, 54.35 USD/ton for medium-temperature, 73.24 USD/ton for high-temperature), leading to a gradual rise in total cash operating costs.

Supplementary Table 20. Comparison of economic indicators and sensitivity factors.

Dimension	Low-temperature Process (300 °C)	Medium-temperature Process (450 °C)	High-temperature Process (600 °C)	Key difference explanation
**Basic Economic Assumptions**				Unified financial assumptions; differences from product value-cost balance
**- Total Investment**	5.25M USD (Fixed 5M + Working Capital 250K)	5.25M USD (Fixed 5M + Working Capital 250K)	5.25M USD (Fixed 5M + Working Capital 250K)	Same EES continuous plant estimate—no investment difference
**- Project Cycle**	30 Years	30 Years	30 Years	Standard long cycle for consistent depreciation/cash flow
**- Tax Rate**	21% (U.S. Federal Income Tax)	21% (U.S. Federal Income Tax)	21% (U.S. Federal Income Tax)	Unified rate to avoid tax policy impact on process economics
**- Operating Rate**	90% (9,000 tons/year)	90% (9,000 tons/year)	90% (9,000 tons/year)	Same rate for consistent output—focus on process balance
**Core Economic Indicators**				High-temp has lower MSP (more feasible); medium-temp has shortest payback but unachievable MSP
**- Main Product MSP**	7,450 USD/ton (C_{18+} Wax)	3,850 USD/ton (C_{18+} Wax)	1,285 USD/ton (Liquid Olefins)	High-temp MSP is 17.2% of low-temp, 33.4% of medium-temp—most feasible
**- Annual Income (Benchmark)**	4,066,020 USD	4,253,820 USD	4,168,440 USD	Medium-temp has slightly higher income; high-temp has more by-product income
**- Annual After-Tax Income**	747,669 USD	761,724 USD	559,965 USD	Medium-temp has highest after-tax income but MSP far above market
**- Annual Operating Cash Flow**	906,002 USD	920,057 USD	718,298 USD	High-temp cash flow is more stable (by-product risk diversification)
**- Payback Period**	5.3 Years	5.1 Years	6.1 Years	High-temp payback is longer but MSP is achievable
**- IRR**	10% (Target)	10% (Target)	10% (Target)	MSP adjusted to hit NPV=0—core is market acceptability of MSP
**Sensitivity Impact**				Low/medium temp sensitive to investment/IRR; high-temp sensitive to by-product prices
**- Fixed Investment +20%**	MSP +$600 (to $8,050/ton)	MSP +$300 (to $4,150/ton)	MSP +$90 (to $1,375/ton)	Low-temp most sensitive—needs big MSP hike to keep IRR

**- Catalyst Cycles -20%**	MSP +$20 (to $7,670/ton)	MSP +$100 (to $3,950/ton)	MSP +$50 (to $1,315/ton)	Low-temp catalyst cost share higher—cycle cuts bit MSP most
**- $NaBH_4$ Price +20%**	MSP +$150 (to $7,600/ton)	MSP +$70 (to $3,920/ton)	MSP +$50 (to $1,315/ton)	Low-temp less product value-more sensitive to catalyst cost
**- By-Product Price +10% (+100% Low)**	MSP -$20 (to $7,430/ton)	MSP -$30 (to $3,820/ton)	MSP -$50 (to $1,235/ton)	High-temp by-product income (45%) drives MSP; low/medium 1% impact
**- IRR +2% (to 12%)**	MSP +$700 (to $8,150/ton)	MSP +$330 (to $4,180/ton)	MSP +$105 (to $1,390/ton)	Low-temp most IRR-sensitive—higher returns need unachievable MSP

For economic indicators, the high-temperature process has the lowest Minimum Selling Price (MSP) (1,285 USD/ton), which is feasible in the market; in contrast, the low/medium-temperature processes have extremely high MSPs (7,450 USD/ton and 3,850 USD/ton respectively) that are difficult to achieve practically, and their investment payback periods also lack advantages. In terms of sensitivity factors, low/medium-temperature processes are highly sensitive to fixed capital investment and Internal Rate of Return (IRR), while the high-temperature process is additionally highly sensitive to by-product prices due to its high proportion of by-product income.

Supplementary Table 21. Cost and Financial Assumptions Summary.

Category	Sub-item	Specification/value	Unit	Remarks
**Raw Material Cost**	Crude Wax	1000 (Usage), 85.71 (Unit Cost), 85.71 (Total Cost)	kg, USD/kg, USD/ton	Converted from 600 CNY/ton (exchange rate 1:7)
	Iron(III) Chloride Hexahydrate	90 (Usage), 0.8 (Unit Cost), 72.00 (Total Cost)	kg, USD/kg, USD/ton	Industrial grade (Sigma - Aldrich price)
	Sodium Borohydride ($NaBH_4$)	180 (Usage), 17.14 (Unit Cost), 3085.20 (Total Cost)	kg, USD/kg, USD/ton	Industrial grade (converted from 120,000 CNY/ton)
	Total Catalyst Raw Material Cost	3157.20	USD/ton	Unallocated
	Allocated Catalyst Cost	31.57	USD/ton	Allocated over 100 cycles (3157.2/100)
	Total Raw Material Cost	117.28	USD/ton	Crude wax cost + allocated catalyst cost
**Financial Assumptions**	Internal Rate of Return (IRR)	10	%	Target value
	Tax Rate	30	%	US federal income tax rate
	Project Lifespan	21	Years	
	Depreciation Method	Straight - line (5% residual value)	-	30 - year depreciation period
	Working Capital Ratio	5% of fixed capital investment	%	
	Operating Rate	90	%	Annual actual processing capacity: 9,000 tons (10000×90%)
**Capital Expenditure**	Fixed Capital Investment	5,000,000	USD	Adjusted based on EES case scale
	Working Capital	250,000	USD	5% of 5,000,000 USD
	Total Investment	5,250,000	USD	Fixed capital + working capital
**Operating Cost**	Cash Operating Cost (Variable)	317.28 (Raw material: 117.28; Energy: 50; Maintenance & Labor: 150)	USD/ton	317.28 USD/ton × 9,000 tons
	Annual Cash Operating Cost	2,855,520	USD	5,000,000 × (1 - 5%) / 30
	Annual Depreciation	158,333	USD	
**Product Revenue**	Refined Wax (Main Product)	640 (Yield), 612 (Unit Price), 391.68 (Revenue)	kg/ton, USD/ton, USD/ton	Main product

	Solid Carbon (By - product)	140 (Yield), 500 (Unit Price), 70.00 (Revenue)	kg/ton, USD/ton, USD/ton	By - product
	Gaseous Hydrocarbons (By - product)	7.6 (Yield), 300 (Unit Price), 2.28 (Revenue)	kg/ton, USD/ton, USD/ton	By - product
	Total By - product Revenue	72.28	USD/ton	Excluding main product

Supplementary References

1. Maloney, James O. Conversion factors and mathematical symbols. *Perry's Chemical Engineers' Handbook (8th Edition), McGraw-Hill Professional Publishing.* (2007).
2. Broadhurst, Martin G. Thermodynamic Properties of Polyethylene Predicted from Paraffin Data. *J. Res. Natl. Bur. Stand. A Phys. Chem.* **67A**, 233 (1963).
3. Stein, R. Brown, Structures and properties group additivity model. *NIST Chemistry WebBook, NIST Standard Reference Database.* 20899 (2009).
