# Supplementary material for: Selective solar wax refining with nanoscale zero-valent iron
Source: Nat Commun. 2026 Mar 30;17:7555. doi: 10.1038/s41467-026-71010-0 (PMC13415555; doi:10.1038/s41467-026-71010-0)
Supplement: Supplementary file 2 — Transparent Peer Review file [file 41467_2026_71010_MOESM2_ESM.pdf]

nature portfolio

Peer Review File

Selective Solar Wax Refining with Nanoscale Zero-Valent Iron

Corresponding Author: Professor Lizhi Zhang

This file contains all reviewer reports in order by version, followed by all author rebuttals in order by version.

Version 0:

Reviewer comments:

Reviewer #1

(Remarks to the Author)
This manuscript introduced a photon-phonon coupling method using nanoscale zero-valent iron (nZVI) and sunlight to refine polyethylene wax, reducing its carbon-chain dispersity (Đ) from 2.5 to 2.0 in one step with > 80% selectivity. Unlike conventional thermochemical methods, which non-selectively break C-C bonds, this approach combines photon-activated C-H bond cleavage and phonon-mediated C-C bond scission via hydrogen atom transfer, enabling precise hydrocracking and evaporative desorption of fragmented wax. The process offers energy-efficient, single-step refinement, highlighting the potential of interfacial photon-phonon coupling for sustainable solar-driven wax upgrading. This work is of broad interest to solar-driven wax refining and selective conversion of polyolefins, and we would recommend its publication after revision. Our major concern is related to the lack of comparison with the conventional thermochemical method. Comments are listed below.
1. To establish a rigorous structure-activity relationship, the electronic and geometric structure and coordination environment of the catalyst should be characterized by advanced techniques such as X-ray absorption spectroscopy. Furthermore, this study highlights the importance of nanoparticles in photothermal conversion. Therefore, size distribution of the nZVI catalyst should be provided.
2. To verify the proposed mechanism and distinguish the role of photon-phonon coupling from pure phonon, a control experiment of thermal wax refining is essential.
3. The universality of the proposed photon-phonon coupled refining strategy is testified by various types of raw waxes, including microcrystalline wax and Fischer-Tropsch wax. However, to assess the robustness of the strategy, the stability of the nZVI catalyst should be tested. Furthermore, to reveal the advantages and limitations of the method, the authors should investigate the influence of reaction parameters on the performance, such as reactant-to-catalyst ratios, reaction temperature, and time.
4. To correctly quantify the selectivity of solar-driven wax refining, quantitative analysis for non-wax components (C5-C17) is recommended for both raw wax and refined wax.
5. This solar-refining strategy effectively utilizes sunlight and nZVI, achieving an impressive PE wax purity up to 99%. Whether this approach achieve comparable refining performance for microcrystalline wax and Fischer-Tropsch wax?
6. The authors claimed that the raw wax was selectively transformed into refined wax without generating toxic aromatic hydrocarbons. To support this claim, UV-Vis absorption spectra of the products should be provided.
7. Commercial trademark is included in Supplementary Fig. 23, which should be removed or blurred to comply with the journal's ethical guidelines.

Reviewer #2

(Remarks to the Author)
The authors claim to have developed a catalyst (Fe@Fe2O3-x) that selectively transforms low-quality commercial waxes into high-quality waxes with a narrow carbon chain length distribution, powered exclusively by solar energy. The study presents a detailed characterization and proposes a mechanism that details the possible reactions involved in the wax refining process. The article presents important advances and may be published after a minor revision.
Row 149 – Please enter references on wax market values.
Row 387 - Methods – Please insert the complete characterization of the raw waxes used.
Row 394 - Please provide details on how the different fractions (wax products, wax residue, spent catalyst) were collected and separated. Please provide details on the procedure for separating the catalyst from the processed waxes.
Row 409 – Please review the information “i include gas product”.
Row 375 – Discussion- Please include in the discussion section how the process could be scaled up. Detail how the raw

materials would be fed, products and waste removed, and how the catalyst regeneration would be handled. Also, include a brief comparison of the results with traditional processes.

Reviewer #3

(Remarks to the Author)
In the manuscript, the authors present a conceptually appealing strategy for solar-assisted wax upgrading using nZVI catalysts. The paper is written in polished English, and the figures are carefully prepared with a professional quality that makes the results visually clear and easy to follow. However, despite these strengths, key confusions exist in the experimental details and mechanistic interpretation that require substantial additional evidence and clarification. These issues significantly weaken the reliability of the conclusions and must be resolved before the work can be considered for publication.

1. In the title, the term “Photon-Phonon Coupling” was claimed. However, the photon- and phonon-driven contributions are not convincingly clarified. One key piece of evidence is shown in Figure 3, where only insignificant differences in performance could be observed between NIR, VIS+NIR, and UV+VIS+NIR, where NIR photons are always present. Merely heating the catalyst bed to 280 °C is not a fair comparison to localized photothermal hotspots (~600 °C as claimed). What will happen if the temperature is just raised to 600 °C? What if the system is heated to 300 or 600 °C when the NIR photons are removed from the light?

2. Following my first question, what exactly are the roles of the thermal effect and the light in the reaction pathway? In Figures 5a and 5b, the positions of the signals are quite similar, although their intensities may differ (as indicated by the arbitrary units on the y-axis). The EPR results show the absence of carbon radicals, but this does not validate the claimed carbocation-mediated β-scission pathway. There is no reference for Figure 5f to explain the radicals detected.

3. In the Methods, 100 mg of catalyst was mixed with 100 mg of wax. I understand that the high ratio between catalyst and wax is to improve the performance, but I am quite curious about how this was achieved to ensure satisfactory contact and avoid burying the catalysts in the wax. If the catalyst cannot absorb light and deliver the charges conveniently, the photocatalytic contribution will be very limited. Also, how was the product quantified if the wax was mixed with the catalyst? Is the catalyst separated from the mixture for the quantification? What procedures were followed to mitigate the mass loss during the product analysis? Such experimental details are not provided in the current manuscript.

4. In the stability test, it is evident that both selectivity and conversion increase during the four cycles. Then why was the test not continued to, e.g., 10 cycles, to demonstrate how the performance behaves? Was the catalyst separated and mixed with fresh wax during the stability measurements?

5. Four light intensities were compared throughout the manuscript. But in Figure 2e, the 4.0 W/cm2, which is the working condition, was not shown. Is there any reason for that?

6. There are not many optimizations on the catalyst. How will the performance change when the synthetic parameters change? Control experiments should also be provided, for example, using commercial iron oxide or iron metal.

7. Comparing the present study with other reported wax conversion technologies and their performance will better demonstrate the progress made in this study.

8. The economic claim of achieving $2,500/ton added value is misleading because it neglects the cost of the photosystems. The high light intensity of 4 W/cm^2 is roughly equivalent to 40 suns, not real sunlight. In other words, there is a need for a strong electricity-powered light source to drive the reaction. The claim ignores issues such as scalability, reactor design, catalyst stability, and separation costs. Without a realistic techno-economic or life-cycle analysis, the manuscript risks exaggerating its practical significance.

9. Some typos: “phonons can be are controlled”, “unfeasible”, “which subsequently accelerate C-C cleavage”.

Version 1:

Reviewer comments:

Reviewer #1

(Remarks to the Author)
The authors addressed my concerns adequately, and I recommend this manuscript forpublication in Nature Communications.

Reviewer #2

(Remarks to the Author)
The article has been carefully revised. All my suggestions have been duly considered. The article can be accepted in its

current form.

Reviewer #3

(Remarks to the Author)
The authors have made a substantial effort to address the concerns raised in my initial review, demonstrating that uniform thermal heating alone cannot account for the observed selectivity and that light irradiation plays an active role beyond bulk heating. The data support a sequential picture in which photons promote non-equilibrium C–H activation at the catalyst–wax interface, while phonons generated by carrier relaxation provide localized thermal energy that enables controlled C–C scission and rapid evaporative product removal.

However, since NIR photons are always present in Figure 3, and the conversion and selectivity of pure NIR appear best among the experiments under the irradiation conditions of NIR,
VIS+NIR, and UV+VIS+NIR, although the differences in performance are only minor.
I still wonder: where is pure NIR, excluding UV+VIS, the key condition for the “photon” part? I guess pure UV-Vis plus heating will not deliver similar performance.

Therefore, the term “photon–phonon coupling” overstates the level of mechanistic evidence achieved. The authors should moderate this terminology and more carefully frame the mechanism as light-assisted localized thermochemistry. With this clarification, the work may be suitable for publication.

Open Access This Peer Review File is licensed under a Creative Commons Attribution 4.0 International License, which permits use, sharing, adaptation, distribution and reproduction in any medium or format, as long as you give appropriate credit to the original author(s) and the source, provide a link to the Creative Commons license, and indicate if changes were made.
In cases where reviewers are anonymous, credit should be given to 'Anonymous Referee' and the source.
The images or other third party material in this Peer Review File are included in the article’s Creative Commons license, unless indicated otherwise in a credit line to the material. If material is not included in the article’s Creative Commons license and your intended use is not permitted by statutory regulation or exceeds the permitted use, you will need to obtain permission directly from the copyright holder.
To view a copy of this license, visit https://creativecommons.org/licenses/by/4.0/

Point-by-point responses to the comments

Notes:

- Our point-by-point responses and revisions of editing in the revised manuscript and Supporting Information are highlighted in color blue.
- The line and page numbers referred to those in the revised manuscript and Supplementary information.

Reviewers' comments:

Reviewer #1: This manuscript introduced a photon-phonon coupling method using nanoscale zero-valent iron (nZVI) and sunlight to refine polyethylene wax, reducing its carbon-chain dispersity (Đ) from 2.5 to 2.0 in one step with > 80% selectivity. Unlike conventional thermochemical methods, which non-selectively break C-C bonds, this approach combines photon-activated C-H bond cleavage and phonon-mediated C-C bond scission via hydrogen atom transfer, enabling precise hydrocracking and evaporative desorption of fragmented wax. The process offers energy-efficient, single-step refinement, highlighting the potential of interfacial photon-phonon coupling for sustainable solar-driven wax upgrading. This work is of broad interest to solar-driven wax refining and selective conversion of polyolefins, and we would recommend its publication after revision. Our major concern is related to the lack of comparison with the conventional thermochemical method. Comments are listed below.

Response: We appreciate the critical comments raised by Reviewer 1 to further improve the quality of our manuscript. We have provided more supplement experimental data, XAFS results, Substrate and product characterization, revised discussions as well as related references to support the conclusion and universality.

Comment 1. To establish a rigorous structure-activity relationship, the electronic and geometric structure and coordination environment of the catalyst should be characterized by advanced techniques such as X-ray absorption spectroscopy. Furthermore, this study highlights the importance of nanoparticles in photothermal conversion. Therefore, size distribution of the nZVI catalyst should be provided.

Response: Thanks for your kind suggestions. We provided the X-ray absorption spectra of the pure catalyst (0 h), spent catalyst obtained after 3 h and 15 h reaction in **Supplementary Fig. 18.** During

the reaction within 15 h, the bulk Fe(0) composition of nZVI was maintained, as evidenced by the close X-ray absorption near edge structure (XANES) spectra for nZVI-0 h, 3 h, 5 h, only with a slight shift of the white line as the reaction proceeded, suggesting the slight reduction of nZVI. Consistently, the XAFS spectra of nZVI-0 h, 3 h, 5 h showed a common main peak at 2.1 Å that is assignable to low-crystallinity Fe(0) with shortened Fe-Fe bond length. As the reaction proceeded, the intensity of the Fe-Fe peak increased, signifying the increased crystallinity of nZVI under photothermal heating. Above results agreed well with our XPS result that the thin iron oxide shell within core-shell structured Fe@Fe_2O_{3-x} nZVI was partially reduced from Fe(III) to Fe(II). The related discussion was added in the revised Supporting Information as follows:

"Furthermore, X-ray absorption near edge structure (XANES) and fine structure (XAFS) spectra also evidenced the reduction of nZVI during the reaction, with the bulk material maintained near Fe(0) (Supplementary Fig. 18)." **(Page 14, line 251-253)**

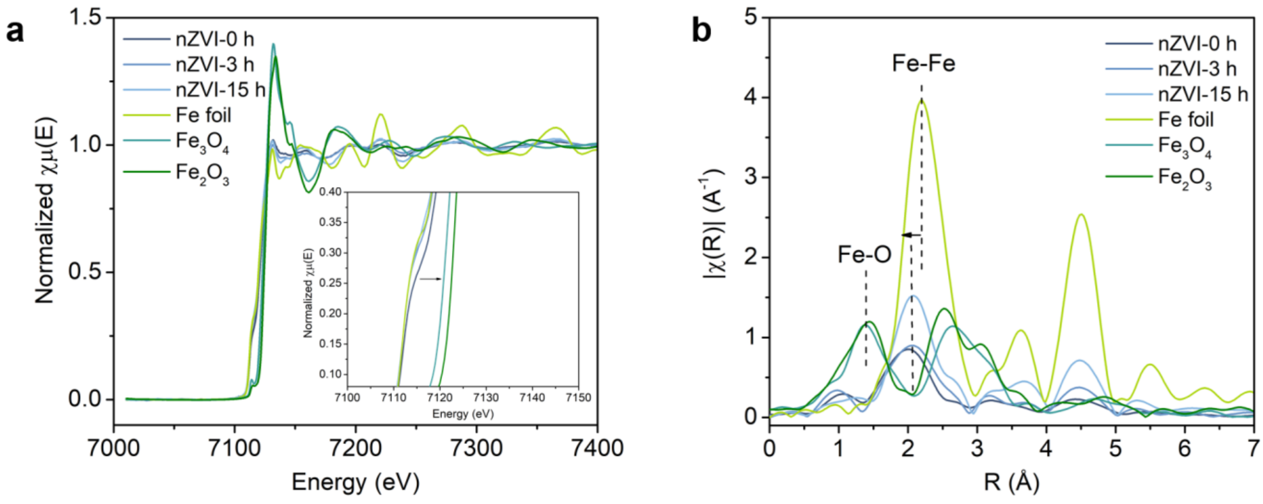

Supplementary Fig. 18 (a) Fe K-edge XANES spectra and (b) EXAFS spectra in R-space for pure catalyst (nZVI-0 h), residue after reaction 3 h (nZVI-3 h) and 15 h (nZVI-15 h).

We supplemented the statistical size distribution of nZVI in **Supplementary Fig. 1.** The diameter of nZVI is between 40-240 nm, with an average size of 120 nm.

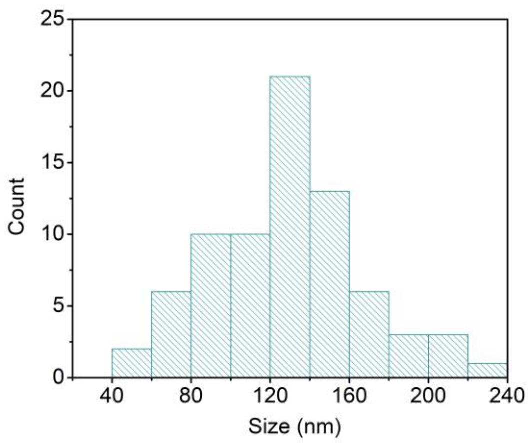

Supplementary Fig. 1 Statistical size distribution of nanospheres in nZVI.

Comment 2. To verify the proposed mechanism and distinguish the role of photon-phonon coupling from pure phonon, a control experiment of thermal wax refining is essential.

Response: Thanks for your kind suggestions. We supplemented the data of thermal wax refining in **Fig. 2b, Fig. 3a and Supplementary Fig. 25-26**. This study demonstrates that the localized hotspot temperature at nZVI under 4.0 W/cm^2 irradiation (inferred to be ~600 °C) significantly exceeds the bulk temperature measured by the thermocouple (280 °C). To elucidate the underlying mechanism and differentiate the contribution of photon-phonon coupling from pure phonon effects, we conducted control experiments under three conditions: dark at 280 °C, dark at 450 °C, 450 °C with ultraviolet and visible light (UV-Vis) irradiation, dark at 600 °C, and 600 °C with UV-Vis light irradiation. The raw wax conversion ratios were 17%, 28% and 56% under dark heating at 280 °C, 450 °C and 600 °C, respectively. In comparison, wax conversion surged to 76% under UV-Vis irradiation at 600 °C and 74% under UV-Vis-NIR irradiation without external heating, both exceeded that under dark thermal conditions.

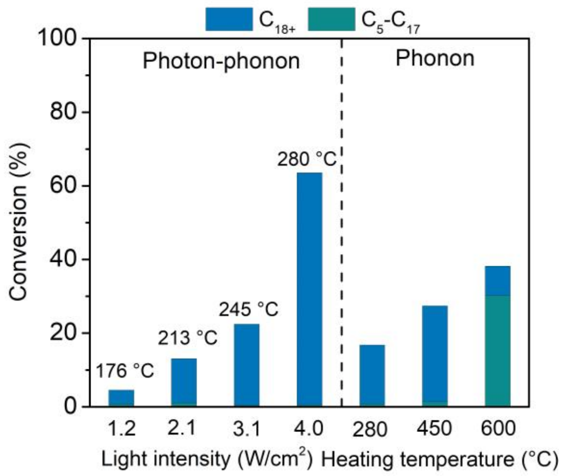

Fig. 2b The C_5-C_{17} and C_{18+} content of wax products obtained under different light intensities and dark heating.

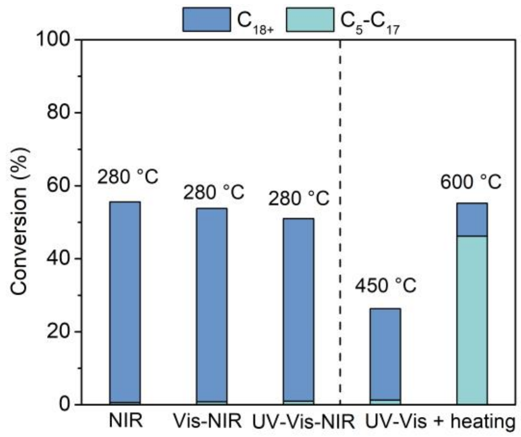

Fig. 3a Content of C_5-C_{17} and C_{18+} in wax product under UV-Vis-NIR, Vis-NIR, NIR irradiation at 280 °C, UV-Vis irradiation with heating at 450 °C and 600 °C.

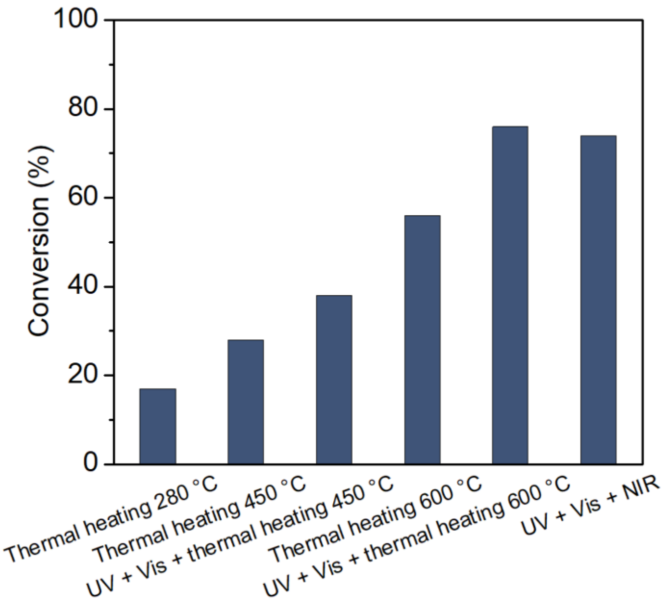

Supplementary Fig. 25 Conversion ratio of raw wax under dark 280 °C, dark 450 °C, 450 °C with UV-Vis light, dark 600 °C, 600 °C with UV-Vis light and UV-Vis-NIR light without heating.

We further analyzed the quantitative products, including gas products (C_1-C_4 and H_2), liquid products (C_5-C_{17}) and wax components (C_{18+}) by GC-MS and GC-FID. Dark heating at 280 °C gave a selectivity of 97% for C_{18+}, but the wax conversion was merely 17%. The increase in heating temperature shifted the carbon number of the product towards lower ones **(Supplementary Fig. 26a)**, exemplified by 12% gas, 70% liquid, and 18% wax products obtained under 600 °C. Product distributions under UV-Vis irradiation and 450 °C and 600 °C thermal heating was close to those under pure dark heating **(Supplementary Fig. 26b)**.

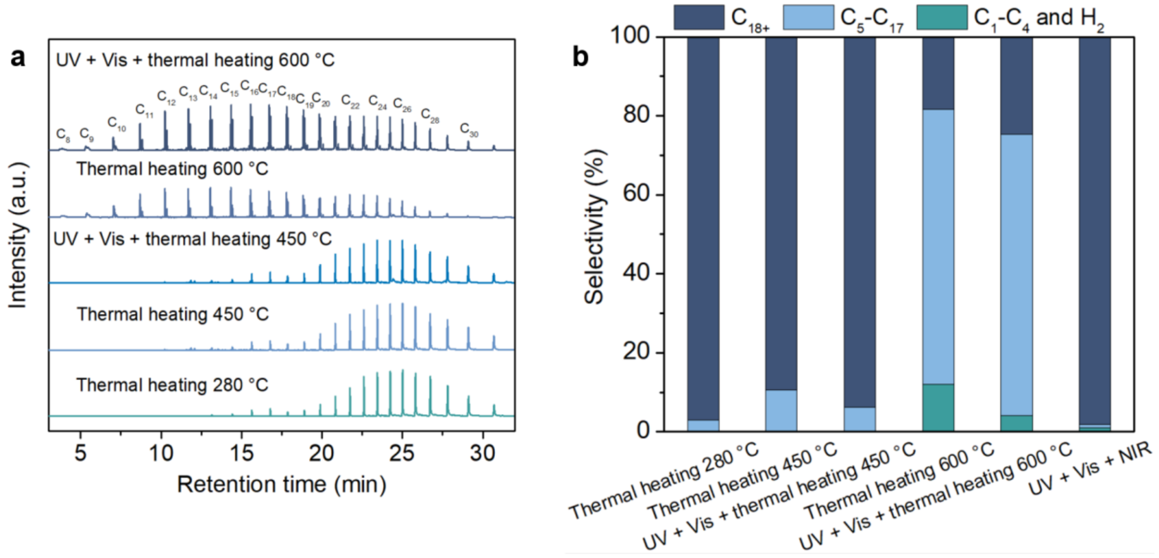

Supplementary Fig. 26 (a) GC-MS spectrum of the liquid and solid product dissolved in n-hexane under dark 280 °C, dark 450 °C, 450 °C with UV-Vis light, dark 600 °C, 600 °C with UV-Vis light. (b) Selectivity of products under dark 280 °C, dark 450 °C, 450 °C with UV-Vis light, dark 600 °C, 600 °C with UV-Vis light and UV-Vis-NIR light without heating.

These findings collectively indicate that traditional thermal heating is insufficient for efficient wax refining. The localized high temperature due to the photon-induced phonon is essential for achieving high raw wax conversion and high selectivity. The related discussion was added in the revised Supporting Information as follows:

"Replacing the local high temperature field with a bulk thermo-heating, the wax product reduced by 8-26% even at same (280 °C) or higher (450 and 600 °C) catalyst-bed temperature (Fig. 2b, Supplementary Fig. 25-29)." **(Page 19, line 355-357)**

Comment 3. The universality of the proposed photon-phonon coupled refining strategy is testified by various types of raw waxes, including microcrystalline wax and Fischer-Tropsch wax. However, to assess the robustness of the strategy, the stability of the nZVI catalyst should be tested. Furthermore, to reveal the advantages and limitations of the method, the authors should investigate the influence of reaction parameters on the performance, such as reactant-to-catalyst ratios, reaction temperature, and time.

Response: Thanks for your kind suggestions. We added the stability of the nZVI catalyst for refining microcrystalline wax and Fischer-Tropsch wax in **Supplementary Fig. 37,** and the related discussion was added in the revised Supporting Information as follows:

"In 10 cycles, the conversion for both Fischer-Tropsch wax and microcrystalline wax were above 70%, suggesting the stability of the nZVI material." **(Page 21, line 396-398)**

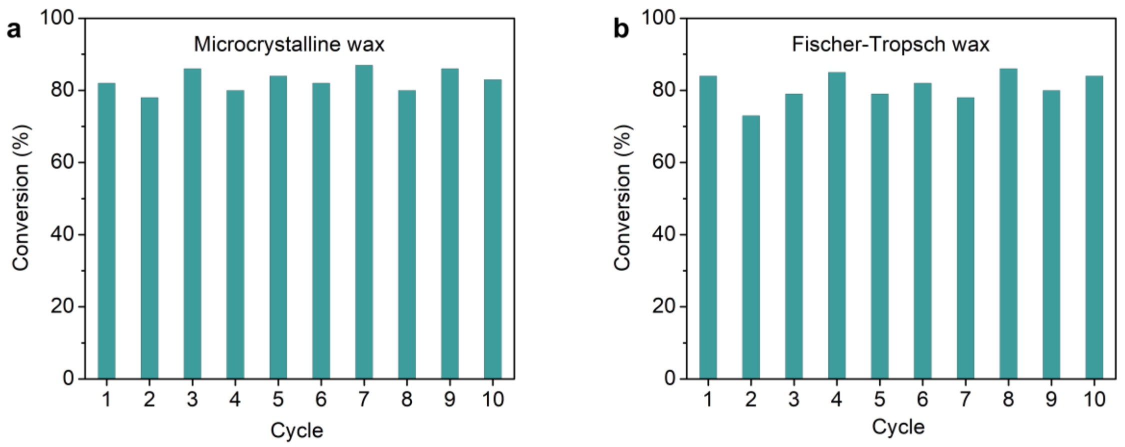

Supplementary Fig. 37 Stability experiments of the nZVI catalyst for photon-phonon coupling driven refining (a) Fischer-Tropsch wax and (b) microcrystalline wax.

We carried out the activity test under reactant-to-catalyst ratios of 1: 0.2, 1: 0.5, 1: 1 and 1: 2, in **Supplementary Fig. 4**, with corresponding conversion of 51%, 65%, 71% and 68%, respectively. Therefore, we selected the optimized reactant-to-catalyst as 1:1 in the following experiment. Please find reply to comment 2 for control activity tests under different reaction temperature and the kinetic study in the main text for the influence of reaction time. **(Page 5, line 103-104)**

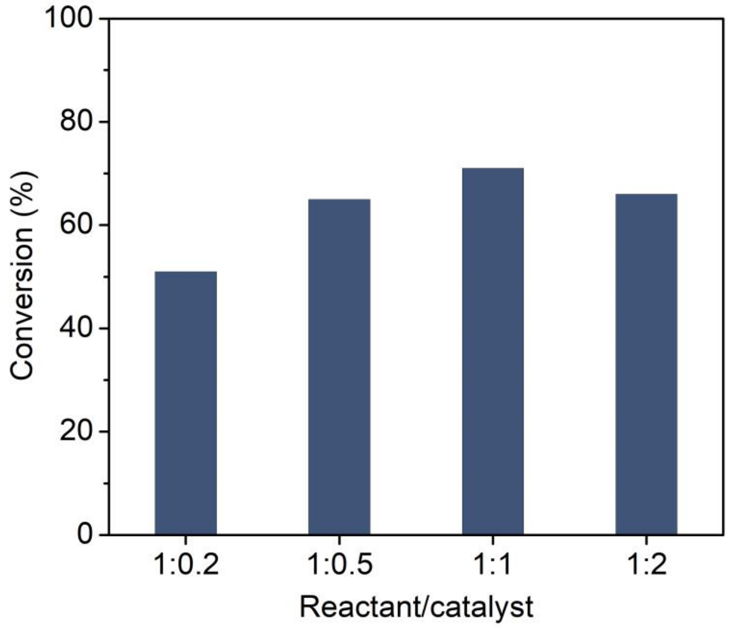

Supplementary Fig. 4 The raw conversion ratio with reactant-to-catalyst ratios of 1:0.2, 1:0.5, 1:1 and 1:2 under 4.0 W/cm^2 light.

Comment 4. To correctly quantify the selectivity of solar-driven wax refining, quantitative analysis for non-wax components (C_5-C_{17}) is recommended for both raw wax and refined wax.

Response: Thanks for your insightful comments. The non-wax components (C_5-C_{17}) in refined wax were quantified by GC-FID in **Fig. 2b and Fig. 3a**. The data of non-wax components (C_5-C_{17}) in raw wax were added in **Supplementary Fig. 35**, where non-wax components (C_5-C_{17}) in semi-refined PE-wax, microcrystalline wax, Fischer-Tropsch wax and crude PE-wax were 0%, 0%, 0% and 0.08%, respectively. These results strongly support the reported selectivity in the main text as non-wax components are negligible.

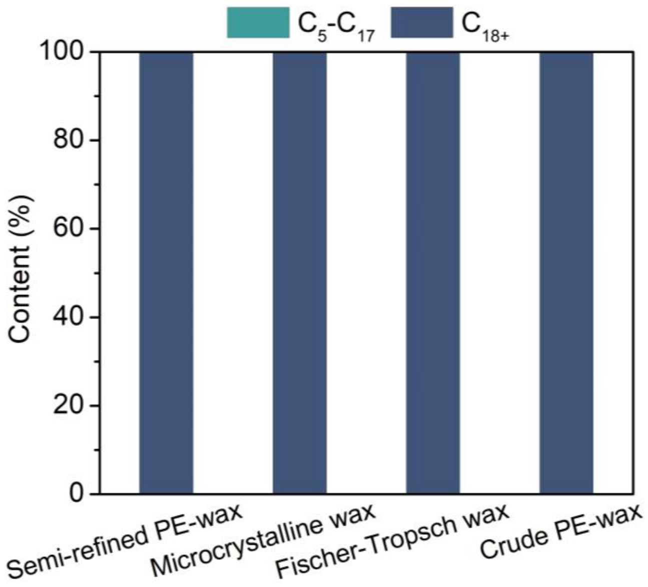

Supplementary Fig. 35 The content of non-wax components (C_5-C_{17}) in semi-refined PE-wax, microcrystalline wax, Fischer-Tropsch wax and crude PE-wax.

**Reactant**	**C_5-C_{17} before reaction**	**C_{18+} before reaction**	**C_5-C_{17} after reaction**	**C_{18+} after reaction**
Semi-refined PE-wax	0	100%	0.54%	99.46%
Microcrystalline wax	0	100%	1.4%	98.6%
Fischer-Tropsch wax	0	100%	0.92%	99.08%
Crude PE-wax	0.08%	99.92%	1.0%	99.0%

Comment 5. This solar-refining strategy effectively utilizes sunlight and nZVI, achieving an impressive PE wax purity up to 99%. Whether this approach achieve comparable refining

performance for microcrystalline wax and Fischer-Tropsch wax?

<u>Response:</u> We provided the proton distribution data of wax refining products for Fischer-Tropsch wax and microcrystalline wax via ^{1}H NMR, as substrates in **Supplementary Fig. 36**. Under light intensity of 4 W/cm^2, no aromatic hydrocarbons were detected in the refined microcrystalline wax and Fischer-Tropsch wax obtained through this solar-driven wax refining strategy. The olefinic proton ratios were 0.44% and 0.55% respectively, indicating that the purity of the refined wax products achieved 99%. **(Page 21, line 394-396)**

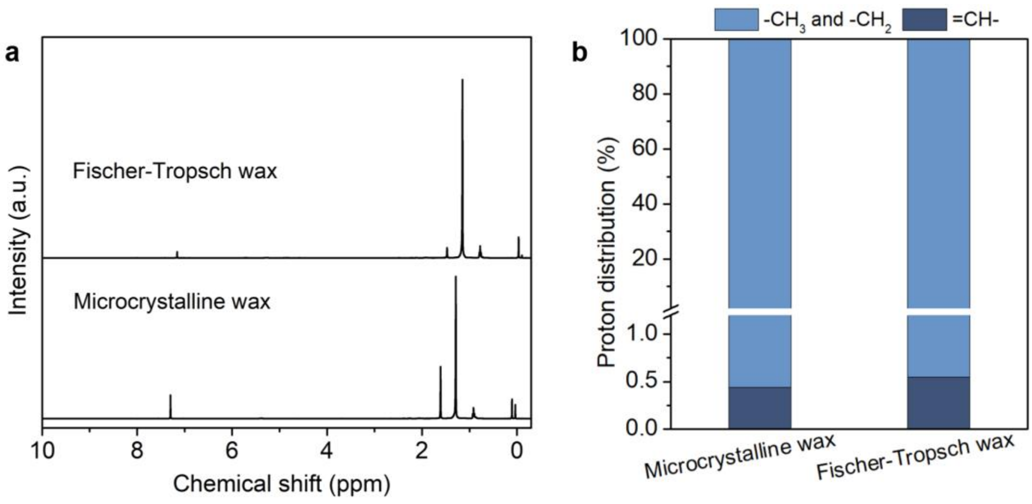

Supplementary Fig. 36 (a) ^{1}H NMR spectra and (b) proton distribution data of wax refining products using Fischer-Tropsch wax and microcrystalline wax as substrates quantified by ^{1}H NMR.

Comment 6. The authors claimed that the raw wax was selectively transformed into refined wax without generating toxic aromatic hydrocarbons. To support this claim, UV-Vis absorption spectra of the products should be provided.

<u>Response:</u> Thanks for your suggestion. UV-Vis absorption spectra of the products dissolved in n-hexane were added in **Supplementary Fig. 8.** The weak absorption peak of UV-Vis spectra at 200 nm was attributed to the π-π* transition of aliphatic olefin (K-band), while the absorption peak at 254 nm, which typically corresponds to the π-π* transition (B-band) of aromatic rings, was not detected.

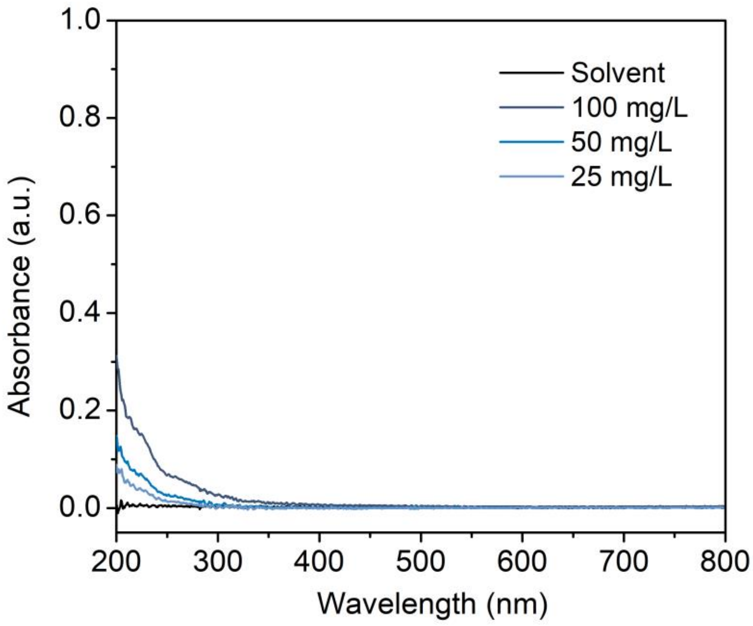

Supplementary Fig. 8 UV-Vis spectra of wax products dissolved in n-hexane.

Our IR and ^{1}H NMR spectra of the wax product indicate an absence of aromatic compounds, and the UV-Vis absorption spectra data is closely consistent with these findings. **(Page 7, line 135-137)**

Comment 7. Commercial trademark is included in Supplementary Fig. 23, which should be removed or blurred to comply with the journal's ethical guidelines.

Response: Thanks for your suggestion. We removed the commercial trademark in **Supplementary Fig. 30** as required.

Reviewer #2: The authors claim to have developed a catalyst ($Fe@Fe_2O_{3-x}$) that selectively transforms low-quality commercial waxes into high-quality waxes with a narrow carbon chain length distribution, powered exclusively by solar energy. The study presents a detailed characterization and proposes a mechanism that details the possible reactions involved in the wax refining process. The article presents important advances and may be published after a minor revision.

Response: We thank the reviewer for the positive comments to our work. We enhanced the quality of our work by supplying the wax market values, separation operation and details.

Comment 1 Row 149 - Please enter references on wax market values.

Response: Thanks for your vital comments. We summarized the wax market values in **Supplementary Table 1.**

**Types**	**Prices ($/ton) †**
Raw wax¶	600
Waste polyethylene wax*	850
Virgin LDPE pyrolysis waxes*	1700-1550
Synthetic LDPE wax*	2800
Synthetic HDPE wax*	5400
High-end grade wax¶	6300

† International or China domestic prices (1 Yuan = 0.14 USD) obtained from ECHEMI, retrieved Oct. 2025.

*Pricing information for waxes as per quotation from Nanjing Tianshi Wax Co., Ltd.

¶Wax pricing based on Sinopec internal quotation.

Comment 2 Row 387 - Methods – Please insert the complete characterization of the raw waxes used.

Response: We thank the reviewer for the comments to our work. Complete characterizations of the raw waxes were added in **Page 29-30, line 524-566.**, including GC-MS, GC-FID, GC-FID/TCD, TH-GPC, ^{1}H NMR, Ex-situ FTIR, XPS and DRS:

GC-FID/TCD: Hydrocarbons (C_1-C_4) and H_2 in the gas products were qualitative and quantitative on a Tet Instrument GC-2030 gas chromatograph with an Al_2O_3 capillary column (30 m × 0.53 mm × 20 μm) and a 5A packed column, equipped with a flame ionization detector (GC-FID) and a thermal conductivity detector (GC-TCD). The injector temperature was 220 °C, and the flame ionization detector and the thermal conductivity detector temperatures were 200 and 70 °C, respectively. The oven temperature was 70 °C and the TCD current was 70 mA.

GC-MS: Hydrocarbons (C_5-C_{28}) in the wax were identified on a Thermo Scientific Trace ISQ 1300 gas chromatograph equipped with a TG-5MS capillary column (30 m × 0.25 mm × 0.25 μm) and a

mass spectrometer (GC-MS). The inlet and ion source temperatures were 200 and 270 °C, respectively. The oven temperature program was: 50 °C holding for 2 min, ramping to 280 °C at a rate of 10 °C /min and holding for 5 min. The carrier gas flow rate was 1.0 mL/min (He) with spitless method. The mass (m/z) range was 40-450 and the solvent delay was 3.5 min.

GC-FID: Hydrocarbons (C_5-C_{28}) in the wax were quantified on a Thermo scientific Trace 1300 gas chromatograph equipped with a TG-5SILMS capillary column (30 m × 0.25 mm × 0.25 μm) and a flame ionization detector (GC-FID). The inlet temperature was 270 °C. The oven temperature program was: 50 °C (hold 2 min), ramping to 280 °C at 10 °C /min (hold 5 min). The peak positions and response factors of linear alkanes were obtained using standard solutions of n-alkanes (C_8-C_{18}) with the 1,3,5-tri-tertbutyl benzene (concentration: 25 mg/L) as an internal standard. All the relative carbon response factors were assumed to be 1.0 in GC-FID analyses.

TH-GPC: Molecular weight distributions of waxes were analyzed on EcoSEC HLC-8321 gel permeation chromatograph. Samples were dissolved in 1,2-dichlorobenzene by heating at 145 °C for at least 1 h. The molecular weight response was calibrated by the polystyrene standards (500-548,0000 g/mol).

^1H NMR: The ^1H NMR spectra were recorded in the Varian NMR Systems 600 MHz spectrometer. Reported chemical shifts (δ, ppm) were calibrated using tetramethylsilane (TMS). NMR specimen was prepared by dissolving wax product in 1 mL of $CDCl_3$ (D 99.8%, TMS 0.003%) and then filtered to remove insolubles. Protons of different chemical environments can be identified within the wax molecule. Furthermore, the integration of proton peak areas and identification of corresponding chemical structures enable detailed quantification.

$$\text{Proton content} = \frac{A_i}{\sum A_i}$$

Where A_i represents the peak area of protons in a certain chemical environment, which include the protons corresponding to $-CH_3$, $-CH_2$ and -CH=CH-.

Ex-situ FTIR spectra: Ex-situ FTIR spectra of wax were recorded on a Tensor 27 spectrometer. Samples (2 mg) were ground with a mortar and pestle, mixed with KBr (200 mg), and pressed into 15 mm diameter pellets using a force of 4 tons.

XPS: Chemical environments of wax and nZVI were analyzed by X-ray photo-electron

spectroscopy (XPS, Thermo Scientific K-Alpha). All reported binding energies were calibrated using the C 1s peak (284.8 eV).

DRS: UV-Vis-NIR diffuse reflectance spectroscopy (DRS) measurement were conducted on a Shimadzu UV-2600 spectrometer. The resolution was 1.0 nm over the range 200-1400 nm and the slit width was 5 nm, with the background spectrum collected based on an optical-grade $BaSO_4$ specimen.

Comment 3 Row 394 - Please provide details on how the different fractions (wax products, wax residue, spent catalyst) were collected and separated. Please provide details on the procedure for separating the catalyst from the processed waxes.

Response: Thanks for your insightful comments. The reaction was carried out in a 50 mL batch reactor filled with Argon under atmospheric pressure. In the optimized experimental procedure, 100 mg of catalyst powder and 100 mg of raw wax powder were physically mixed by shaking and then the mixture was loaded into the batch reactor. Because of nZVI's black color, mixed powder appears black as shown in **Supplementary Fig. 3a**. The reaction proceeds without requiring an external heating. Reaction timing started when the 300 W xenon lamp equipped with focus lens (Microsolar 300, Beijing Perfectlight Technology) was turned on. When the Xe lamp was turned on, the raw wax rapidly melted, forming a solid-liquid interface with the catalyst to trigger the reaction. Driven by localized high temperatures (> 600 °C) generated through the localized surface plasmon resonance (LSPR) of nZVI under light, the product wax vaporizes and separates from the catalyst. Subsequent condensation occurred on the cool reactor interior surfaces (< 100 °C), yielding refined wax **(wax products)** and realizing wax separation simultaneously. As shown in Supplementary Fig. 3c, some waxes condensed on the window of the reactor, forming a "O-ring" pattern and leaving the center where the light penetrate through unaffected. Furthermore, some waxes condensed on the interior wall and the bottom of the reactor (outside the catalyst bed). These condensed waxes were majorly collected via stainless-steel spatula, with residues collected by solvent (n-hexane) washing followed by wax recovery via vaporizing the n-hexane solvent. The catalyst bed after the reaction contains a small portion of **wax residue** (cannot detected by XRD) and dominantly **spent catalyst**, which we did not separate them. The mass of wax residue can be calculated by the difference of the catalyst bed before and after reaction using a analytic balance. Gas products were sampled with GC syringe, and analyzed by GC-FID and GC-TCD (Tet Instrument GC-2030). These

details have been added in Methods, **page 25-26, line 469-488.** The raw photos about raw wax mixed catalyst, wax products and wax residue and spent catalyst have been added in to **Supplementary Fig. 3.**

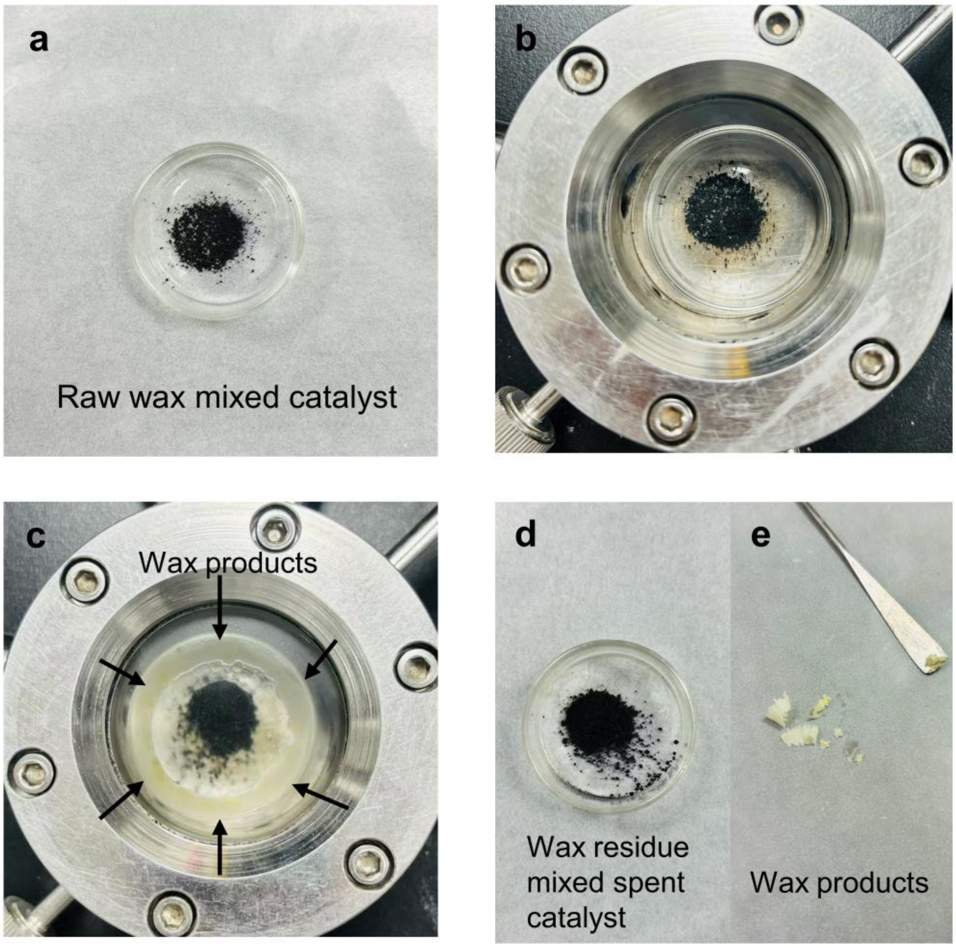

Supplementary Fig. 3 Digital photos for (a) raw wax mixed catalyst, and (b) raw wax mixed catalyst loaded into the reactor before reaction, (c) Product wax condensed on a quartz window, and (d) spent catalyst, and (e) wax products collected from the window and walls using a stainless-steel spatula.

Comment 4 Row 409 – Please review the information "i include gas product".

Response: Thanks for your insightful comments. This sentence was deleted during the revision.

Comment 5 Row 375 – Discussion- Please include in the discussion section how the process could be scaled up. Detail how the raw materials would be fed, products and waste removed, and how

the catalyst regeneration would be handled. Also, include a brief comparison of the results with traditional processes.

Response: We sincerely thank the reviewer for this insightful suggestion. We incorporated a new subsection detailing an upscaled outdoor, natural solar-driven wax refining reaction within the discussion section on **page 22-23, line 412-423**. Crucially, the experimental procedure underscores the inherent simplicity and advantage of our photon-phonon coupling strategy:

Feeding: The 5 g raw wax mixed with 1 g catalyst feedstock is simply loaded directly onto the catalyst bed within the reactor prior to solar irradiation.

Product Removal:

Gas Products (C_1-C_4 and H_2): These low-molecular-weight products are directly collected via an outlet port connected to a gas sampling bag or flow meter as they are generated, requiring no complex separation apparatus.

Wax Product: Upon reaction completion and cooling to room temperature, the desired high-value solid wax product is readily retrieved by manually scraping the interior walls using a stainless-steel spatula. This direct physical removal eliminates the need for any solvent extraction, distillation, or other energy-intensive downstream purification/separation steps, which is a key operational advantage of our approach.

Waste/Solid Residue Handling: The solid residue (approximately 20% mass yield), primarily comprising spent catalyst and minor hydrocarbons, remains on the catalyst bed, which requires no additional separation step and can be directly used for subsequent wax refining process.

The outdoor upscaled experiment was conducted under concentrated natural sunlight (3.6-4.3 W/cm^2) in Shanghai between 13:35 and 15:35 on July 9, 2025. After 2 hours of reaction, the mass yields were: gas products (C_1-C_4 and H_2) ~0.43%, liquid compound (C_5-C_{17}) ~0.7%, target solid wax (C_{18+}) ~62%, and solid residue ~20%. The selectivity towards the valuable wax fraction reached ~98%. These results unequivocally demonstrate that our photon-phonon coupling wax refining strategy is not only effective but also operationally straightforward and readily deployable outdoors under natural sunlight. The elimination of complex separation units and solvent recovery systems, coupled with the simple catalyst regeneration procedure, offers significant scaling and cost advantages over conventional, electricity-intensive processes, presenting highly promising prospects for practical

application. **(Supplementary Fig. 38-39)**

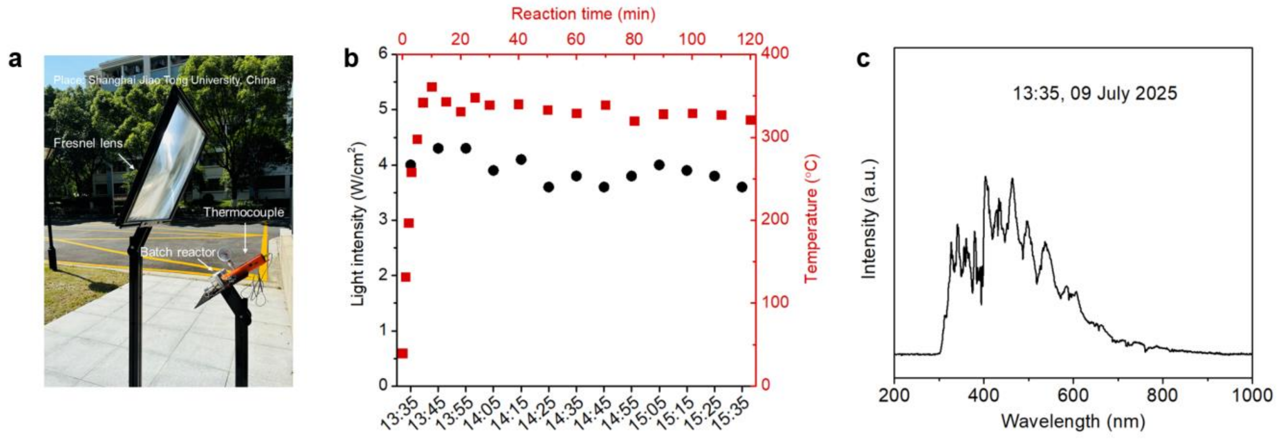

Supplementary Fig. 38 (a) Photograph of reaction device with a Fresnel lens. (b) Temperature profiles of the mixture of 1 g nZVI and 5 g raw wax under designated photothermal reaction. (c) Solar spectrum detected by fiber optic spectrometer at 13:35 on 9 July 2025.

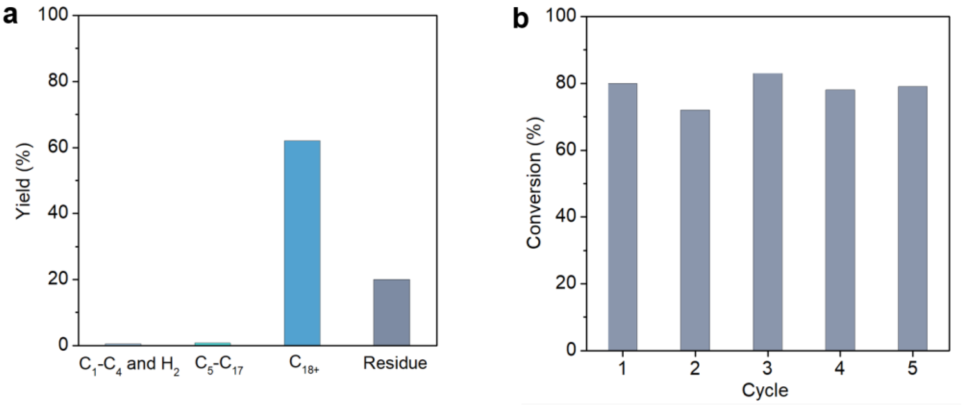

Supplementary Fig. 39 (a) Mass yield of the gas products (C_1-C_4 and H_2), liquid component (C_5-C_{17}), wax component (C_{18+}) and solid residue after reaction 2 h. (b) Reusability of the nZVI catalyst for outdoor solar-driven wax refining.

In addition, it is crucial to compare with the traditional process method. We have added the other reported processes and their performance in **Supplementary Table 2**.

**Catalyst**	**Reaction condition**	**Products and performance**	**Ref.**
$Fe@Fe_2O_{3-x}$	Xe lamp illumination 3 h;<br>Ar atmosphere	Conversion > 70%<br>Refined wax selectivity 99%	***This work***

Pd/Hβ	380 °C; WHSV 1.77 h^{-1}, H_2 180 mL/min	64.8% conversion; 48.5% naphtha-ranged liquid yield	*J. Anal. Appl. Pyrol.* **161**,105424 (2022)
Zeolite Y	450 °C; N_2 atmosphere	60% liquid yield	*Energy Fuels* **35**, 9450-9461 (2021)
HZSM-5	FCC conditions; 500-550 ℃	About 50% gasoline yield; About 70% conversion	*Chem. Eng. J.* **132**, 17-26 (2007)
FCC Equilibrium Catalyst	FCC conditions; 500-560 °C	Conversion 41.4-62.9 wt %; 23.8-31.1 wt % naphtha and 13.7-18.1wt % LPG fractions	*Energy Fuels* **33**, 5191-9199 (2019)
Pt/Al_2O_3	6-8 h; 350 °C; H_2 180 barg	Saturated hydrocarbon	*Energy Fuels* **37**, 16181-16185 (2023)

Current literature on wax conversion remains limited. Research remains predominantly focused on industrial applications, employing the FCC feeding method with zeolite catalysts and pyrolysis temperatures typically exceeding 300 °C. While precious metal-modified catalysts can modestly enhance catalytic performance, the primary products are gasoline- and naphtha-range alkanes, alkenes, and aromatic hydrocarbons. Contrast, our approach enables single-step solar-driven wax refining, achieving >70% conversion with 99% selectivity to refined wax without requiring additional heating and co-reactants. These performance metrics significantly exceed those reported in the existing literature.

Reviewer #3: In the manuscript, the authors present a conceptually appealing strategy for solar-assisted wax upgrading using nZVI catalysts. The paper is written in polished English, and the figures are carefully prepared with a professional quality that makes the results visually clear and easy to follow. However, despite these strengths, key confusions exist in the experimental details and mechanistic interpretation that require substantial additional evidence and clarification. These issues significantly weaken the reliability of the conclusions and must be resolved before the work can be considered for publication.

Response: We thank the reviewer for the positive comments to our work. We enhanced the quality and reliability of our work by supplementing thermal control tests, system optimizations, additional experiments and mechanism analysis during the revision.

Comments 1. In the title, the term "Photon-Phonon Coupling" was claimed. However, the photon- and phonon-driven contributions are not convincingly clarified. One key piece of evidence is shown in Figure 3, where only insignificant differences in performance could be observed between NIR, VIS+NIR, and UV+VIS+NIR, where NIR photons are always present. Merely heating the catalyst bed to 280 °C is not a fair comparison to localized photothermal hotspots (~600 °C as claimed). What will happen if the temperature is just raised to 600 °C? What if the system is heated to 300 or 600 °C when the NIR photons are removed from the light?

Response: Thanks for your insightful comments. We provided the data of thermal wax refining under 450 and 600 °C with and without light irradiation during this revision.

To elucidate the underlying mechanism and differentiate the contribution of photon-phonon coupling from pure phonon effects, we conducted control experiments under 280, 450, and 600 °C with and without ultraviolet and visible light (UV-Vis) irradiation. The raw wax conversion ratios were 17%, 28% and 56% under dark heating at 280, 450 and 600 °C, respectively. In contrast, wax conversions reached 76% under UV+Vis+600 °C thermal heating and 74% under UV-Vis-NIR irradiation, both were substantially higher than under dark conditions.

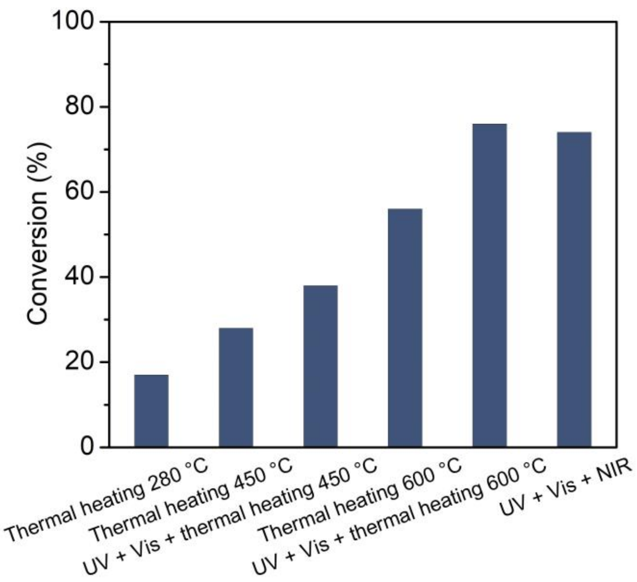

Supplementary Fig. 25 Conversion ratio of raw wax under dark 280 °C, dark 450 °C, 450 °C with UV-Vis light, dark 600 °C, 600 °C with UV-Vis light and UV-Vis-NIR light without heating.

We further analyzed corresponding products, including gas products (C_1-C_4 and H_2), liquid products (C_5-C_{17}) and wax components (C_{18+}) by GC-MS and GC-FID. The results indicate that dark heating at 280 °C yielded 97% of C_{18+}, but the conversion ratio is only 38%. The increase in heating temperature caused the carbon number of the product shifting towards lower ones (Supplementary Fig. 25), as revealed by the wax cracking to generate 12% gas, 70% liquid, and 18% wax products under 600 °C. Product distributions under UV-Vis irradiation with 450 °C and 600 °C thermal heating were comparable to those under pure dark condition (Supplementary Fig. 26).

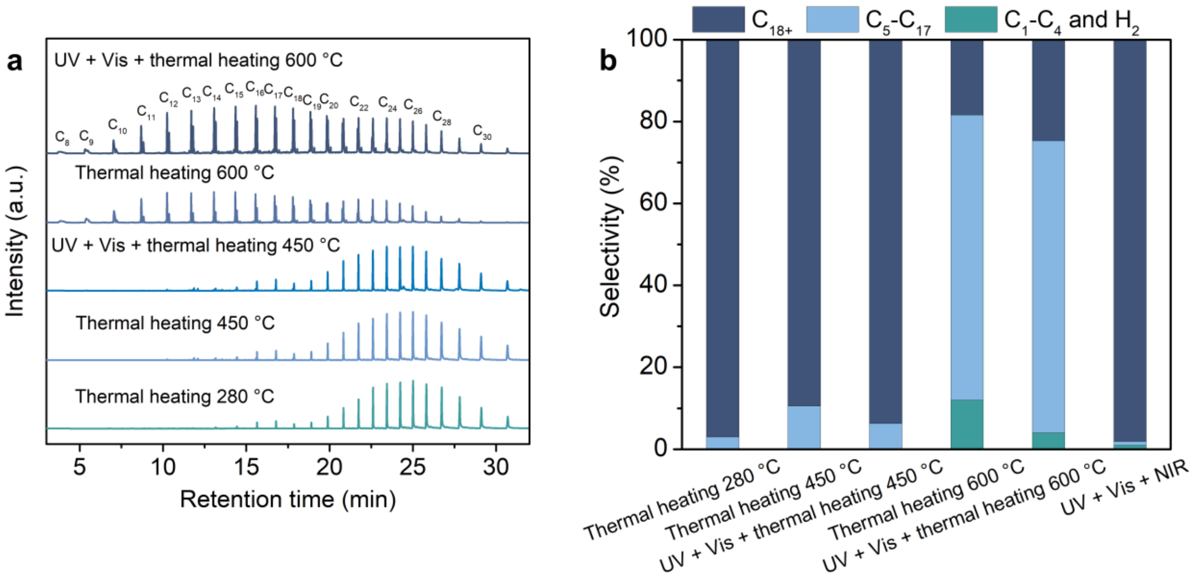

Supplementary Fig. 26 (a) GC-MS spectrum of the liquid and solid product dissolved in n-hexane under dark 280 °C, dark 450 °C, 450 °C with UV-Vis light, dark 600 °C, 600 °C with UV-Vis light. (b) Selectivity of products under dark 280 °C, dark 450 °C, 450 °C with UV-Vis light, dark 600 °C, 600 °C with UV-Vis light and UV-Vis-NIR light without heating.

These findings collectively indicate that traditional thermochemistry is insufficient for efficient wax refining. Critically, the photoexcitation-induced hotspot rather than bulk heat supplement is the crucial factor enabling the high selectivity towards the desired wax product. The related discussion was added in the revised manuscript as follows:

"Replacing the local high temperature field with a bulk thermo-heating, the wax product reduced by 8-26% even at same (280 °C) or higher (450 and 600 °C) catalyst-bed temperature." **(Page 19, line**

355-357)

Comments 2. Following my first question, what exactly are the roles of the thermal effect and the light in the reaction pathway? In Figures 5a and 5b, the positions of the signals are quite similar, although their intensities may differ (as indicated by the arbitrary units on the y-axis). The EPR results show the absence of carbon radicals, but this does not validate the claimed carbocation-mediated β-scission pathway. There is no reference for Figure 5f to explain the radicals detected.

Response: Thanks very much for your insightful suggestions. We adjusted the y-axis of in-situ DRIFTS to the same scale in **Figures 5a and 5b**, and added corresponding reference to the EPR analysis for better understanding.

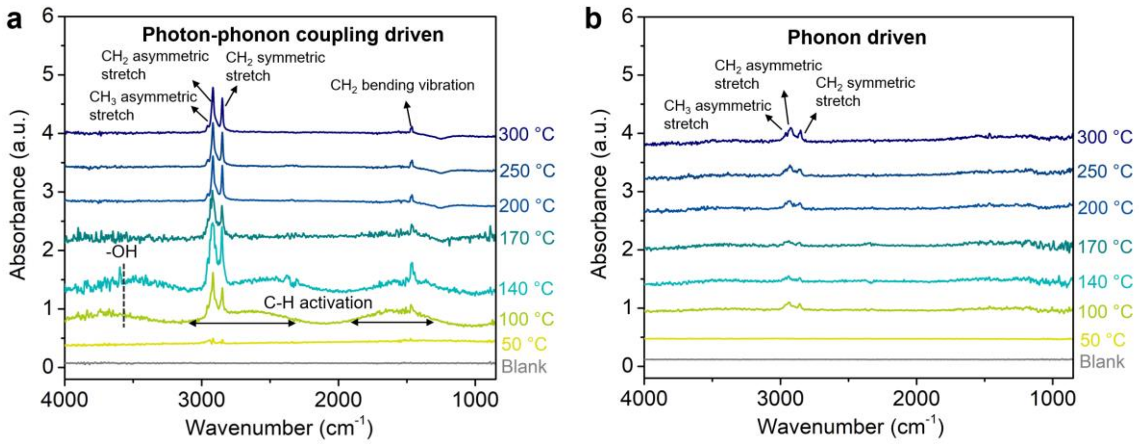

Fig. 5a and 5b In-situ DRIFTS spectra of wax refining driving by (a) photon-phonon coupling and (b) pure phonon.

Through wavelength-dependent measurements and temperature-dependent in-situ DRIFTS measurements, we demonstrate that photons activate C-H bonds (C-H peak broadening and -OH formation) prior to C-C activation (generating small fragments with more $-CH_3$ groups) enabled by phonons, and phonons significantly promoted the C-C cleavage.

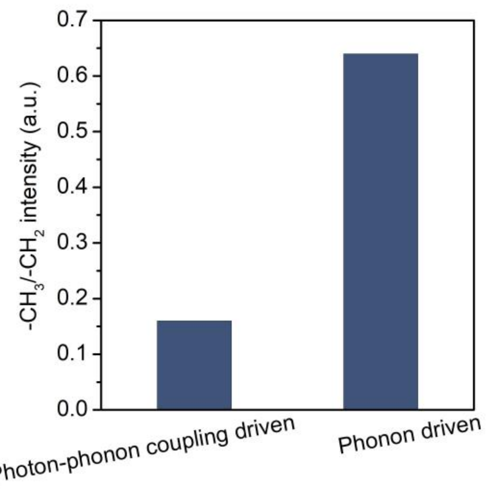

Supplementary Fig. 20 Comparison of $-CH_3/-CH_2$ intensity ratio from in-situ DRIFTS spectra of wax refining driving by photon-phonon coupling and pure phonon.

Comparing DRIFT spectra of photon-phonon coupling and pure phonon processes, the peak intensity under light is larger than that under dark, suggesting the higher reaction degree under light, which agrees well with the activity test.

Furthermore, no C-H activation was observed under dark, and the $-CH_3/-CH_2$ peak intensity ratio under dark was at least 5 times that under light (0.64 vs 0.16), suggesting that phonons preferentially promote C-C cleavage, instead of C-H cleavage, to generate more alkyl fragments with terminal $-CH_3$ group, agreeing well with the result obtained from temperature-dependent DRIFTS under photon-phonon coupling condition **(Supplementary Fig. 20)**. The related discussion was added in the revised manuscript as follows:

"Notably, the $-CH_3/-CH_2$ intensity ratios under light and dark were 0.16 and 0.64, respectively. Given the $-CH_3$ is the terminal moiety of an alkyl fragment, this result indicated that phonon drives more C-C bonds scission in wax and photon-phonon coupling drives the controllable scission C-C bonds." **(Page 17, line 307-310)**

According to the EPR result, termination of the deconstruction reaction was neat over $Fe@Fe_2O_{3-x}$, as evident by a lack of carbon-centered (•R) or oxy-carbon-centered (•OR) radicals in the gas phase when probed by the well-documented spin-trap N-tertiary-butyl nitrone (PBN). All observed EPR triplet signals were from a N-centered O-•N(R)-(R)C=O radical, which was produced by the intermolecular self-oxidation of O^--(R)N^+=(R)C-H. The oxidation of PBN was significant that can be visualized according to the characteristic color change from white to yellow, with EPR

showing consistent enlarged intensity for the triplet signal. This is distinct to the commonly proposed gas-phase radical terminations via mutual combination found in thermal processes.

Having systematically reviewed the fundamentals for the deconstruction of waxy hydrocarbons, all possible reaction intermediates include 1) free radicals, 2) carbanions, and 3) carbocations, corresponding to three different reaction mechanism **(Scheme 1, Reference: 52)**. Since no carbon-centered radicals (•R) were detected by EPR using the spin trap of N-tertiary-butyl nitrone (PBN; Fig. 5f), we can safely exclude the random C-C thermolysis via free radical mechanism **(Reference: 53)**. Carbanion mechanism was also excluded due to the absence of strong bases, reducing agents, or highly polarized chemical environment in our system **(Reference: 54)**. Therefore, the carbocation-mediated C-C scission mechanism is proposed for the nZVI-mediated wax refining. Theoretically, carbocation triggered by nZVI's surface oxides (Fe_2O_{3-x}) is very reasonable as the Lewis acid, Fe^{2+}/Fe^{3+}, dissociates C-C/H bond and accepts electron, while the neighboring lattice oxygen (O^{2-}), a Lewis base, can bind and stabilize the resulting carbocation **(Reference: 55)**. The only question is whether the C-C scission proceeds via a α- or β-scission pathway, with the difference lies in the bond-cleaving site next to or two atoms away from the carbocation site. Experimentally, the difference can be distinguished via the final gaseous product, as the α-scission resulted in majorly CH_4 products while β-scission resulted in less C_{2+} hydrocarbons. As seen from the activity test, conventional thermochemistry produced dominantly gaseous CH_4, while our photon-phonon coupling process generated majorly non-methane gases, suggesting a carbocation-mediated β-scission mechanism over irradiated nZVI. Corresponding revision has been made in the main text, **page 20, line 367-385**.

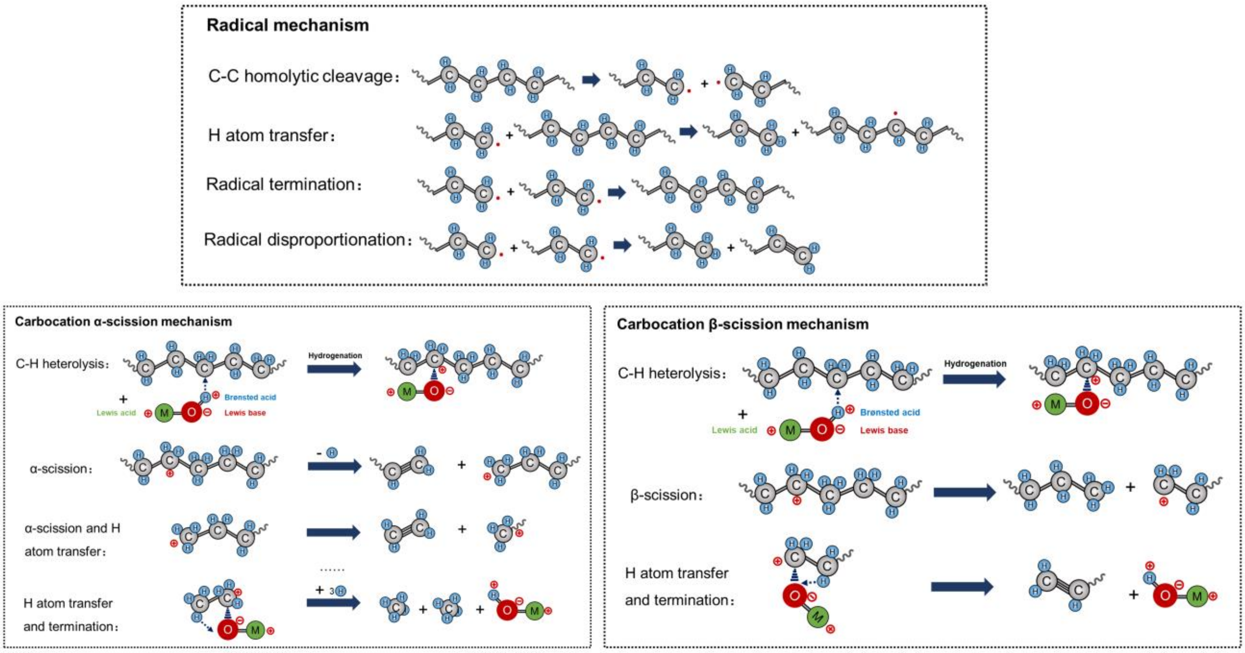

Scheme 1 Polyolefin deconstruction via free radicals, carbocation α-scission and carbocation β-scission mechanism.

Comments 3. In the Methods, 100 mg of catalyst was mixed with 100 mg of wax. I understand that the high ratio between catalyst and wax is to improve the performance, but I am quite curious about how this was achieved to ensure satisfactory contact and avoid burying the catalysts in the wax. If the catalyst cannot absorb light and deliver the charges conveniently, the photocatalytic contribution will be very limited. Also, how was the product quantified if the wax was mixed with the catalyst? Is the catalyst separated from the mixture for the quantification? What procedures were followed to mitigate the mass loss during the product analysis? Such experimental details are not provided in the current manuscript.

Response: Thanks very much for your kind suggestions. The reaction was carried out in a 50 mL batch reactor filled with Argon under atmospheric pressure. In the optimized experimental procedure, 100 mg of catalyst powder and 100 mg of raw wax powder were physically mixed by shaking and then the mixture was loaded into the batch reactor. Because of nZVI's black color, mixed powders appear black color as shown in **Supplementary Fig. 3a**. The reaction proceeds without external heating. Reaction timing started when the 300 W xenon lamp equipped with focus lens (Microsolar 300, Beijing Perfectlight Technology) was turned on. Upon light on, the raw wax rapidly

melted, forming a solid-liquid contact with the catalyst to trigger the interfacial refining reaction. Driven by localized high temperatures (> 600 °C) generated through the localized surface plasmon resonance (LSPR) of nZVI under light, the product wax vaporizes and separates from the catalyst. Subsequent condensation occurred on the cool reactor interior surfaces (< 100 °C), yielding refined wax (wax products) and realizing wax separation simultaneously. As shown in Supplementary Fig. 3c, some waxes condensed on the window of the reactor, forming a "O-ring" pattern and leaving the center where the light penetrate through unaffected. Furthermore, some waxes condensed on the interior wall and the bottom of the reactor (outside the catalyst bed). These condensed waxes were majorly collected via stainless-steel spatula, with residues collected by solvent (n-hexane) washing followed by wax recovery via vaporizing the solvent. The catalyst bed after the reaction contains a small portion of wax residue and dominantly spent catalyst. Instead of separating the residual wax with spent catalyst, we use the mass difference of the catalyst bed before and after reaction to calculate the mass of the residual wax. Furthermore, gas products were sampled with GC syringe, and analysed by GC-FID and GC-TCD (Tet Instrument GC-2030). **Page 25-26, line 469-488.** Digital photos for raw wax mixed with catalyst, wax products, wax residue and spent catalyst have been added in to **Supplementary Fig. 3.**

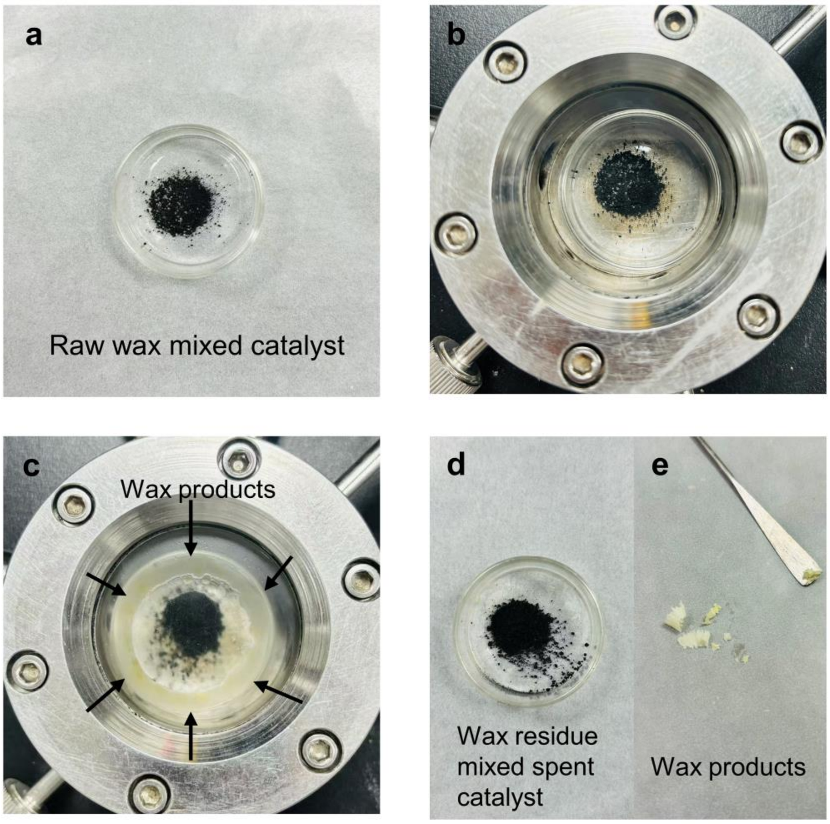

Supplementary Fig. 3 Digital photos about reaction (a) raw wax mixed catalyst, (b) raw wax mixed catalyst loaded into the reactor before reaction, (c) Product wax condensed on a quartz window, and (d) wax residue mixed with spent catalyst after reaction, and (e) wax products collected from the window and walls using a stainless-steel spatula.

Comments 4. In the stability test, it is evident that both selectivity and conversion increase during the four cycles. Then why was the test not continued to, e.g., 10 cycles, to demonstrate how the performance behaves? Was the catalyst separated and mixed with fresh wax during the stability measurements?

<u>Response:</u> Thanks very much for your kind suggestions. We added the data of 11 cycles stability test in **Fig. 5c** during the revision. From the results of the stability test of 11 cycles, the conversion rates of raw wax were all higher than 70%, indicating that coke did not decrease the reactivity of the catalyst. The related discussion was added in the revised manuscript as follows:

"Catalytic longevity test revealed sustained activity of reacted catalyst throughout 11 successive runs, indicating that coke did not decrease the reactivity of the catalyst." **(Page 18, line 339-341)**

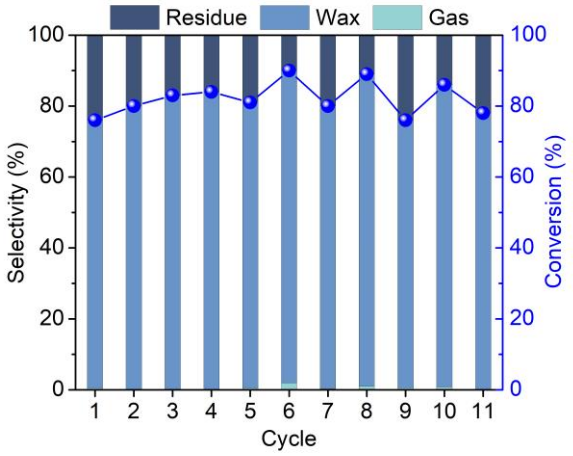

Fig. 5c Reusability of the nZVI catalyst for photon-phonon coupling driven wax refining.

Comments 5. Four light intensities were compared throughout the manuscript. But in Figure 2e, the 4.0 W/cm^2, which is the working condition, was not shown. Is there any reason for that?

<u>Response:</u> Thanks very much for your insightful suggestions. During this revision, we added the result under 4 W/cm^2 in **Fig. 2e**.

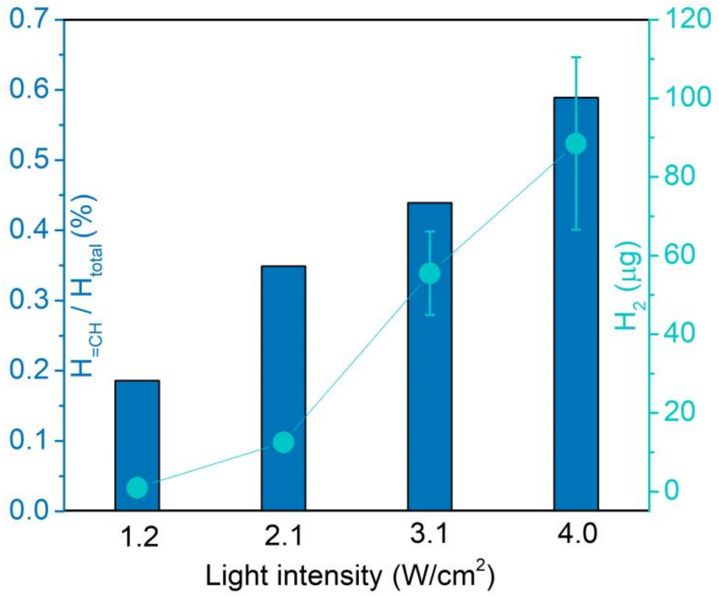

Fig. 2e Proportion of olefinic protons in wax products obtained under different light intensities quantified by ^{1}H NMR and corresponding H_2 production during the refining process.

Comments 6. There are not many optimizations on the catalyst. How will the performance change when the synthetic parameters change? Control experiments should also be provided, for example, using commercial iron oxide or iron metal.

Response: Thanks very much for your insightful suggestions. We supplemented the data of wax refining by commercial Fe powder, Fe_2O_3, Fe_3O_4 in **Supplementary Fig. 11.**

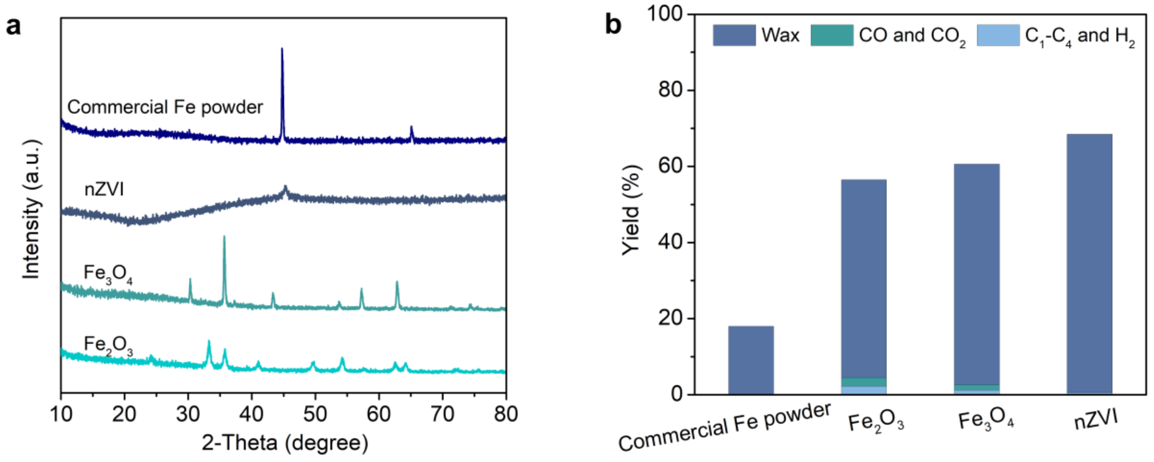

Supplementary Fig. 11 (a) XRD patterns of commercial Fe powder, Fe_2O_3, Fe_3O_4, nZVI-0.5 h, nZVI-1 h and nZVI-2 h. (b) The yield of products after 3 h reaction by commercial Fe powder, Fe_2O_3, Fe_3O_4,

nZVI-0.5 h, nZVI-1 h and nZVI-2 h under 4 W/cm^2.

Results demonstrated that during photon-phonon coupled wax refining, wax constituted the primary product across all tested iron oxides (Fe_2O_3 and Fe_3O_4). Notably, nZVI achieved higher wax yields but less CO_2/CO than commercial Fe powder and iron oxides. The related discussion was added in the revised manuscript as follows:

"In comparison, the wax refining capacity of control samples including commercial Fe powder, Fe_2O_3, and Fe_3O_4, or conventional thermochemical wax were significantly lower (< 60% wax yield; Supplementary Fig. 11). This result highlights not only our highly efficient nZVI material but also the nZVI-enabled photon-phonon coupling strategy, which co-establishes a paradigm for sustainable, high-efficiency wax upgrading (Supplementary Table 2)." **(Page 8, line 155-160)**

Comments 7. Comparing the present study with other reported wax conversion technologies and their performance will better demonstrate the progress made in this study.

Response: Thanks very much for your insightful suggestions. We compared the other reported wax conversion technologies and their performance in **Supplementary Table 2.**

Catalyst	Reaction condition	Products and performance	Ref.
$Fe@Fe_2O_{3-x}$	Xe lamp illumination 3 h; Ar atmosphere	Conversion > 70% Refined wax selectivity 99%	***This work***
Pd/Hβ	380 °C; WHSV 1.77 h^{-1}, H_2 180 mL/min	64.8% conversion; 48.5% naphtha-ranged liquid yield	*J. Anal. Appl. Pyrol.* **161**,105424 (2022)
Zeolite Y	450 °C; N_2 atmosphere	60% liquid yield	*Energy Fuels* **35**, 9450-9461 (2021)
HZSM-5	FCC conditions; 500-550 °C	About 50% gasoline yield; About 70% conversion	*Chem. Eng. J.* **132**, 17-26 (2007)
FCC Equilibrium Catalyst	FCC conditions; 500-560 °C	Conversion 41.4-62.9 wt %; 23.8-31.1 wt % naphtha and 13.7-18.1wt % LPG fractions	*Energy Fuels* **33**, 5191-9199 (2019)
Pt/Al_2O_3	6-8 h; 350 °C; H_2 180 bar	Saturated hydrocarbon	*Energy Fuels* **37**, 16181-16185 (2023)

Studies on wax refining remain limited. Previous work predominantly focused on industrial pyrolysis processes, employing the FCC feeding method with zeolite catalysts and pyrolysis temperatures typically exceeding 300 °C. While precious metal-modified catalysts can modestly enhance catalytic performance, the primary products are gasoline- and naphtha-range alkanes, alkenes, and aromatic hydrocarbons. In contrast, our approach enables single-step solar-driven wax refining, achieving >70% conversion with 99% selectivity to refined high-quality wax without requiring additional heating and co-reactants. These performance metrics significantly exceed those reported in the existing literature.

Comments 8. The economic claim of achieving $2,500/ton added value is misleading because it neglects the cost of the photosystems. The high light intensity of 4 W/cm^2 is roughly equivalent to 40 suns, not real sunlight. In other words, there is a need for a strong electricity-powered light source to drive the reaction. The claim ignores issues such as scalability, reactor design, catalyst stability, and separation costs. Without a realistic techno-economic or life-cycle analysis, the manuscript risks exaggerating its practical significance.

Response: Thanks for your kind suggestions. We agree that the electricity-powered wax refining is not economically competitive, and the real sunlight-powered one can make a difference. To support the economic feasibility of our laboratory prototype, we designed an outdoor solar-driven wax refining experiment using a upscaled reactor, equipped with a 0.5 m * 0.5 m Fresnel lens, as demonstrated in **Supplementary Fig. 38**.

The solar-driven wax refining was conducted in Shanghai, China between 13:35 and 15:35 on July 9, 2025, with 5 g raw wax and 1 g nZVI catalyst loaded. Concentrated via the Fresnel lens, the sunlight intensity reached to the reactor was 3.6-4.3 W/cm^2. After 2 hours of reaction, the mass yields were: gas products (C_1-C_4 and H_2) ~0.43%, liquid compound (C_5-C_{17}) ~0.7%, target solid wax (C_{18+}) ~62%, and solid residue ~20%. The selectivity towards the value-added wax reached ~98%. These results unequivocally demonstrate that our photon-phonon coupling wax refining strategy is efficient and readily deployable under sunlight irradiation. The elimination of complex separation units and solvent recovery systems, coupled with the simple catalyst regeneration procedure, offers significant scaling and cost advantages over conventional, electricity-intensive processes, indicating a promising technology for practical application. **(Page 22-23, line 412-423)**

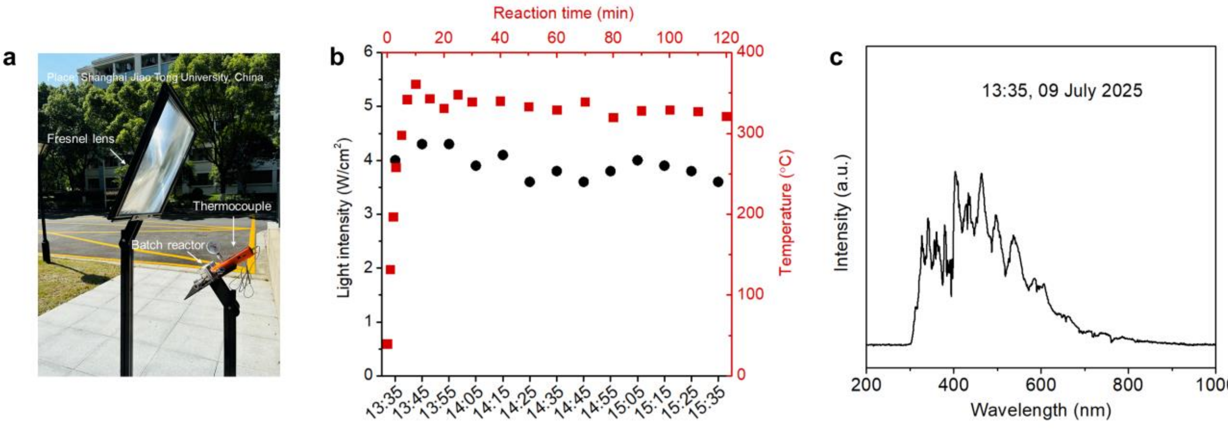

Supplementary Fig. 38 (a) Photograph of reaction device with a Fresnel lens. (b) Temperature profiles of the mixture of 1 g nZVI and 5 g raw wax under designated photothermal reaction. (c) Solar spectrum detected by fiber optic spectrometer at 13:35 on 9 July 2025.

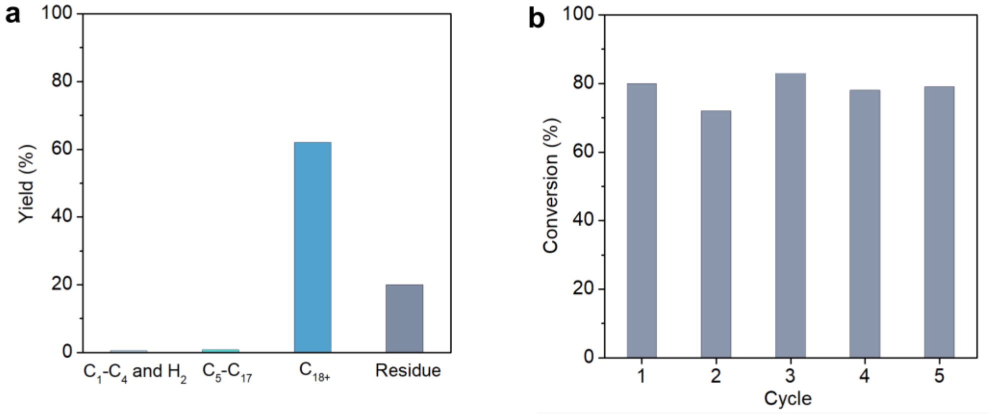

Supplementary Fig. 39 (a) Mass yield of the gas products (C_1-C_4 and H_2), liquid component (C_5-C_{17}), wax component (C_{18+}) and solid residue after reaction 2 h. (b) Reusability of the nZVI catalyst for outdoor solar-driven wax refining.

Based on above results, we conducted rigorous techno-economic and life-cycle analysis to assess the potential of the solar-driven wax refining, as shown in **Fig 6e-6f and Supplementary Fig. 40-45 on page 23-24, line 424-445.** The analysis focuses on the techno-economic feasibility of the chemical projects (with catalytic cracking of refined wax as the core), using the Discounted Cash Flow (DCF) model as the primary tool and centering on the financial objective of "calculating the Minimum Selling Price (MSP) of the main product to achieve NPV = 0 and IRR = 10%". It follows a consistent logic: first, defining the basic project settings (such as general parameters including a 30-year project lifecycle, 90% operating rate, 21% tax rate, and a total investment of USD 5.25 million);

second, breaking down investment costs (fixed investment + working capital) and operating costs (variable costs including raw materials, catalysts, and energy, as well as fixed costs including labor and maintenance); third, compiling a long-term cash flow statement to iteratively solve for the MSP; and finally, identifying risk points through sensitivity analysis and comparing different technical routes.

The TEA and LCA were conducted based on a solar driven wax refining facility capable of processing 10 kt per year of raw wax (Supplementary Fig. 41). The raw wax is refined by the above-mentioned solar-driven system using nZVI catalyst. The minimum selling prices (MSPs) for our solar driven strategy and conventional thermochemical method at 300, 450 and 600 °C are shown in **Supplementary Fig. 42.** Our mild operating conditions enable a minuscule total utility. During the 10-year project life cycle, the net profits of the thermal heating 300 °C, 450 °C, 600 °C and solar driven wax refining strategies were 7450 (C_{18+} wax), 3850 (C_{18+} wax), 1285 (C_5-C_{17} fuel oil) and 612 (C_{18+} wax) US $/ton raw wax, and corresponding global warming potential (GWP) values were 21815, 10901, 16358 and 516 kg CO_2-eq/kg refined wax, respectively. It is noteworthy that all of products from our route exhibit 10 kt CO_2-eq emission reduction per tonne of refined wax compared to that of thermal heating, contributing to the goal of carbon neutrality.

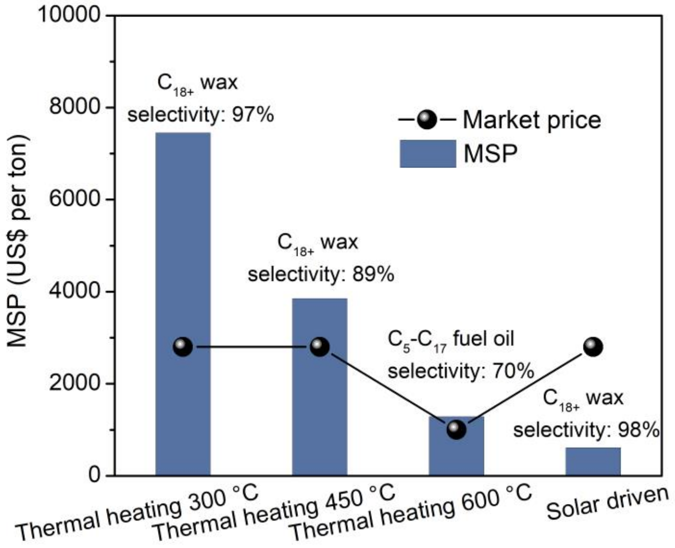

Fig. 6e TEA of a conceptual solar driven wax refining facility capable of processing 10 kt per year of raw wax. MSP of main products (thermal heating 300 °C, 450 °C and solar driven: C_{18+} wax; thermal heating 600 °C: C_5-C_{17} fuel oil) and their average market price.

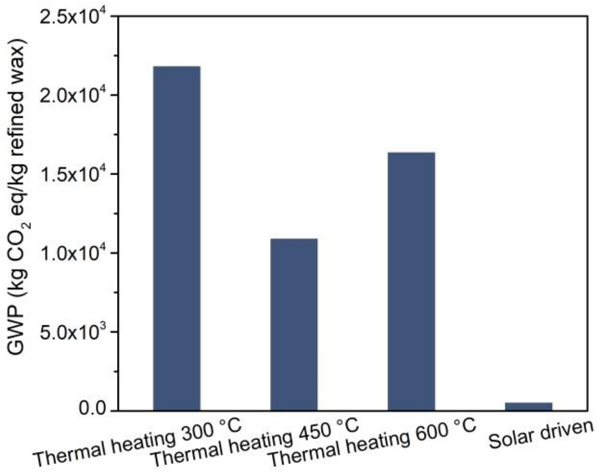

Fig. 6f GWP of the thermal heating 300 °C, 450 °C, 600 °C and solar driven wax refining strategies.

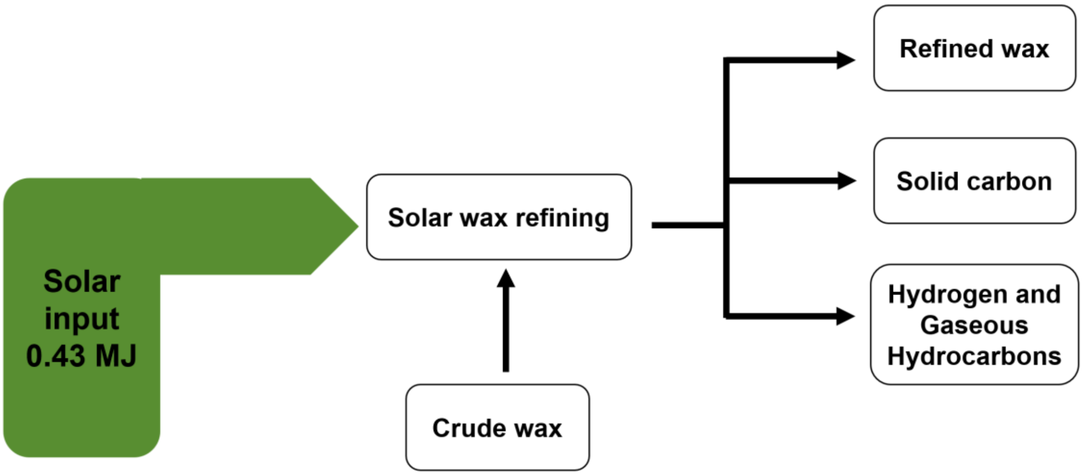

Supplementary Fig. 40 Process flowsheet of solar driven wax refining demonstration plant with 10 kt/year processing intake.

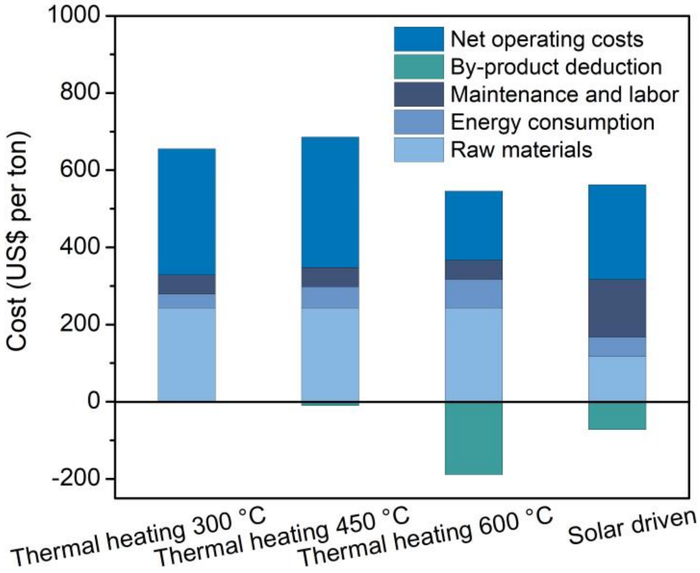

Supplementary Fig. 41 TEA of the solar-driven wax refining process. Costs of the thermal heating 300 °C, 450 °C, 600 °C and solar driven wax refining strategies. The product distribution were obtained from Supplementary Fig. 28.

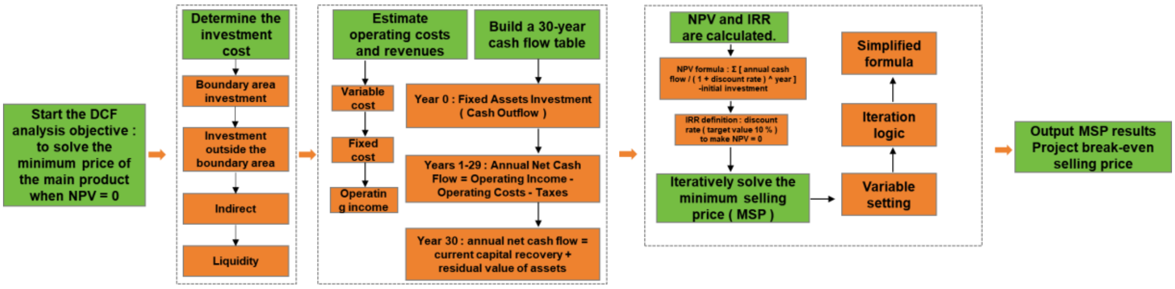

Supplementary Fig. 42 EES Finance.

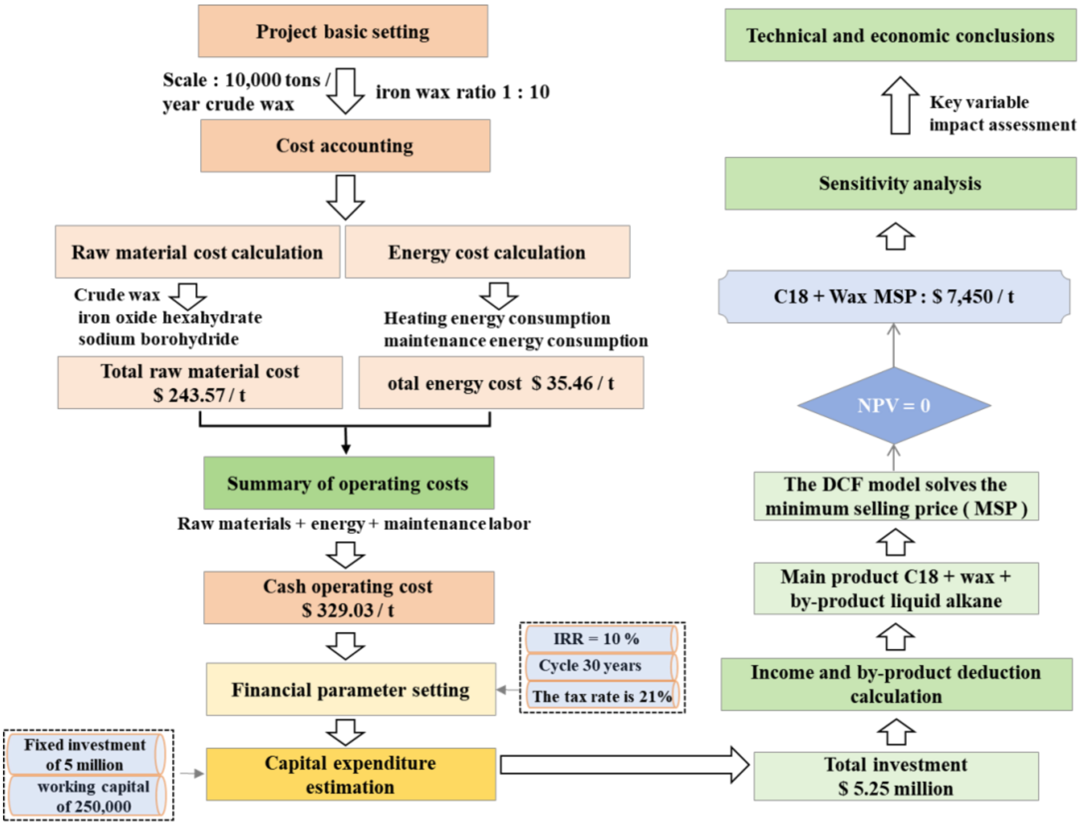

Supplementary Fig. 43 Core Cost & Investment Parameters of low temperature catalytic cracking refined wax.

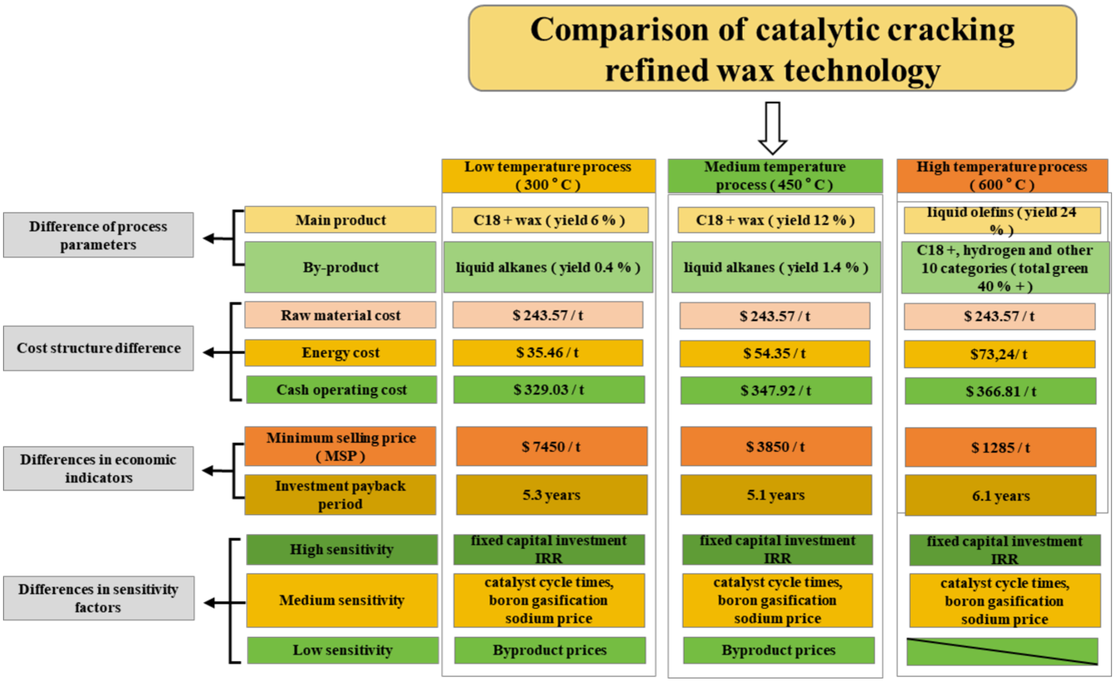

Supplementary Fig. 44 Comparison of catalytic cracking refined wax technology.

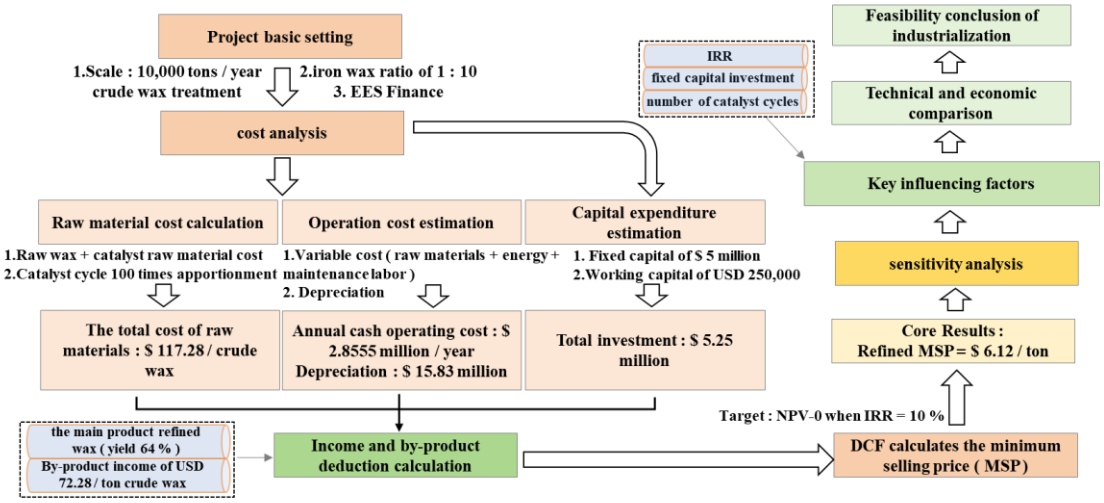

Supplementary Fig. 45 Technical and economic analysis of solar catalytic refining wax.

Comments 9. Some typos: “phonons can be are controlled”, “unfeasible”, “which subsequently accelerate C-C cleavage”.

Response: Thanks for your kind suggestions. These typos have been corrected during the revision.

Point-by-point response

Notes:

- Our point-by-point responses and revisions of editing in the revised manuscript and Supporting Information are highlighted in color blue.
- The line and page numbers referred to those in the revised manuscript and Supplementary information.

Reviewer #3: The authors have made a substantial effort to address the concerns raised in my initial review, demonstrating that uniform thermal heating alone cannot account for the observed selectivity and that light irradiation plays an active role beyond bulk heating. The data support a sequential picture in which photons promote non-equilibrium C-H activation at the catalyst–wax interface, while phonons generated by carrier relaxation provide localized thermal energy that enables controlled C-C scission and rapid evaporative product removal.

However, since NIR photons are always present in Figure 3, and the conversion and selectivity of pure NIR appear best among the experiments under the irradiation conditions of NIR, VIS+NIR, and UV+VIS+NIR, although the differences in performance are only minor.

I still wonder: where is pure NIR, excluding UV+VIS, the key condition for the “photon” part? I guess pure UV-Vis plus heating will not deliver similar performance.

Therefore, the term “photon-phonon coupling” overstates the level of mechanistic evidence achieved. The authors should moderate this terminology and more carefully frame the mechanism as light-assisted localized thermochemistry. With this clarification, the work may be suitable for publication.

Response: We appreciate for your insightful comments. According to your suggestion, we changed the title of the manuscript "**Solar-driven Photon-Phonon Coupling for Efficient Wax Refining with Nanoscale Zero-Valent Iron**" to "**Selective Solar Wax Refining with Nanoscale Zero-Valent Iron**". All the descriptions of "**photon-phonon coupling**" in the main text, supplementary information and figures have been modified to "**light-assisted localized thermochemistry**" or other relative words such as "**thermal/photothermal, dark/light, localized heating**".

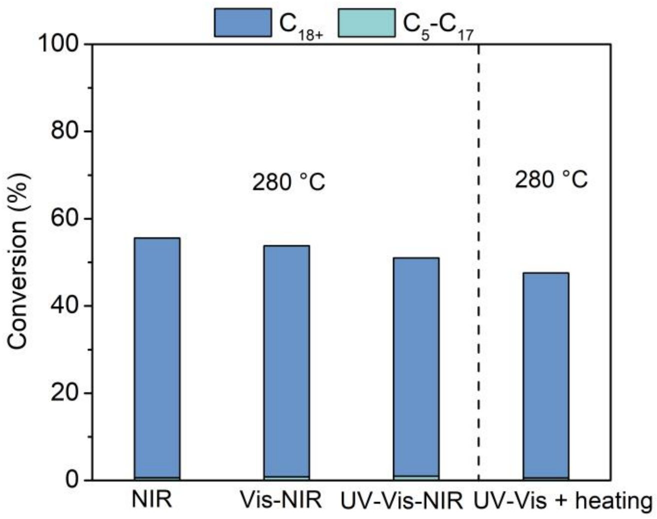

Fig. 3a Content of C_5-C_{17} and C_{18+} in wax product under UV-Vis-NIR, Vis-NIR, NIR irradiation at 280 °C, UV-Vis irradiation with thermal heating at 280 °C.

We supplemented the data of wax refining under UV-Vis plus thermal heating at 280 °C in **Fig. 3a**. In comparison, wax yield decreased from 50% under UV-Vis-NIR to 47% under UV-Vis irradiation, while the wax selectivity 1.1%. This result suggests illustrating the light-assisted localized thermochemistry mechanism. **(Page 11, line 202-204)**
